# Plasticity of mitotic cyclins in promoting the G_2_–M transition

**DOI:** 10.1083/jcb.202409219

**Published:** 2025-04-09

**Authors:** Adrijana Crncec, Ho Wai Lau, Lau Yan Ng, Hoi Tang Ma, Joyce P.Y. Mak, Hon Fung Choi, Tsz Kwan Yeung, Randy Yat Choi Poon

**Affiliations:** 1 Division of Life Science, The Hong Kong University of Science and Technology, Clear Water Bay, Hong Kong; 2 Laboratory of Cancer Biology and Genetics, Center for Cancer Research, National Cancer Institute, Bethesda, MD, USA; 3 Department of Pathology, The University of Hong Kong, Pok Fu Lam, Hong Kong; 4 State Key Laboratory of Liver Research, The University of Hong Kong, Pok Fu Lam, Hong Kong; 5 State Key Laboratory of Molecular Neuroscience, The Hong Kong University of Science and Technology, Clear Water Bay, Hong Kong

## Abstract

Cyclins and cyclin-dependent kinases (CDKs) orchestrate key events in the cell cycle. However, the uniqueness of individual mitotic cyclins has been a long-standing puzzle. By rapidly removing cyclins in G_2_ human cells, we found that deficiency of B-type cyclins attenuates mitotic onset and uncouples the G_2_–M kinase network from mitosis, resulting in sustained activation of PLK1 and cyclin A–CDK1. This culminates in mitotic slippage without completing nuclear envelope breakdown. Remarkably, elevating cyclin A several-fold above its endogenous level is adequate to restore mitosis, allowing cells to survive without B-type cyclins. In contrast, cyclin A is rate-limiting but not essential for G_2_–M due to compensation by endogenous cyclin B1–CDK2, a non-canonical pair. These findings challenge the traditional indispensable roles of different cyclins and highlight their plasticity. Due to the high malleability of the A- and B-type cyclins, cancer cells may be able to place different weights on different cyclins, while maintaining sufficient CDK activities for successful mitosis.

## Teaser

The study reveals that individual mitotic cyclins’ roles are more flexible than previously thought. Deficiency of B-type cyclins inhibits mitotic entry, leading to pre-NEBD mitotic slippage. Remarkably, elevating cyclin A levels can restore mitosis independently of B-type cyclins. These findings challenge traditional views on cyclin functions.

## Introduction

The cell cycle is choreographed by an evolutionarily conserved engine composed of cyclin-dependent kinases (CDKs) and their activating cyclin subunits (reviewed in Poon [2021]). The current paradigm assigns different cyclin–CDK complexes in regulating distinct cell cycle processes: cyclin D–CDK4/6 for G_1_, cyclin E–CDK2 for G_1_–S, cyclin A–CDK1/2 for S and mitosis, and cyclin B–CDK1 for mitosis (Morgan, 2006).

Cyclin B and CDK1 are integral components of the M phase-promoting factor (MPF). Cyclin B accumulates from the S phase, persists through G_2_, and diminishes after mitosis (reviewed in Fung and Poon [2005]). MYT1/WEE1-dependent phosphorylation of CDK1^T14/Y15^ ensures that cyclin B–CDK1 complexes remain inactive in interphase. Activation of cyclin B–CDK1 is initiated by the PLK1 pathway through CDC25 and MYT1/WEE1, leading to autocatalytic activation that drives mitosis (reviewed in Poon [2021]). Destruction of cyclin B at the end of mitosis is mediated by the ubiquitin ligase APC/C (reviewed in Zhou et al. [2016]).

Unlike cyclin B, cyclin A serves dual functions in both the S phase and mitosis (reviewed in Yam et al. [2002]). Cyclin A and cyclin B have distinct evolutionary histories. While cyclin B is conserved in slime mold, fungi, and animals, cyclin A is absent in fungi and slime mold (Cao et al., 2014). Furthermore, cyclin A and cyclin B have different cell cycle expression profiles, subcellular localizations, and CDK partners in human cells (reviewed in Yam et al. [2002]). During the S phase, cyclin A–CDK2 phosphorylates components of the pre-replicative complexes, facilitating replicative origins unwinding and prevention of origin refiring (Coverley et al., 2002). The precise functions of cyclin A during mitosis are less defined, with hypotheses involving cyclin A itself as a component of MPF or as part of the network that facilitates MPF activation (reviewed in Lindqvist et al. [2009]).

The presence of multiple mitotic cyclins in metazoans remains a long-standing puzzle. Cyclin B1, but not cyclin B2, is essential for development in mice (Brandeis et al., 1998). In human cells, RNAi studies indicate that cyclin B2 is either dispensable (Bellanger et al., 2007; Gong et al., 2007; Soni et al., 2008) or is involved in cell proliferation in specific cell lines (Wu et al., 2021; Xiao et al., 2022). More recent CRISPR-based analyses indicate that while cyclin B1 is an essential gene in 962/1,100 (87%) of human cell lines, cyclin B2 is essential in 6/1,100 (0.5%) of cell lines (Tsherniak et al., 2017).

Considering the critical role of cyclin B1 in early mouse development (Brandeis et al., 1998), it is surprising that silencing cyclin B1 in human cell lines produces relatively mild effects. Multiple RNAi-based studies have demonstrated that downregulation of cyclin B1 does not induce G_2_ arrest in HeLa, HCT116, or RPE1 cells (Bellanger et al., 2007; Chen et al., 2008; Gong et al., 2007; Gong and Ferrell, 2010; Soni et al., 2008; Yuan et al., 2006). Depletion of both cyclin B1 and B2 is required to induce a delay in G_2_ (Soni et al., 2008; Yuan et al., 2006). Using a degron strategy that allows more robust silencing of cyclin B1 and B2, Hégarat et al. demonstrated that the loss of cyclin B1 and B2 does not affect mitotic entry in RPE1 cells (Hégarat et al., 2020). The cells are capable of initiating chromosome condensation, nuclear envelope breakdown (NEBD), and spindle formation. However, they exhibit defects in sister chromatid segregation and cytokinesis. It seems hardly surprising that such a wealth of information would also give rise to ambiguities and contradictions, as other reports argue that the knockdown of cyclin B1 alone is sufficient to induce a G_2_ delay and reduce proliferation in HeLa and several breast cancer cell lines (Androic et al., 2008; Xie et al., 2005; Yuan et al., 2004).

Cyclin A (the major somatic isoform, cyclin A2) is an essential gene for early embryonic development in mice (Murphy et al., 1997) and is indispensable in human RPE1 cells (Hégarat et al., 2020). However, experiments with conditional gene ablation indicated that while cyclin A is dispensable in mouse fibroblasts, it is essential in hematopoietic and embryonic stem cells (Kalaszczynska et al., 2009). Given cyclin A’s dual functions in the S phase and mitosis, the exact cause of lethality upon cyclin A disruption remains unclear. For example, the G_2_–M defects observed after cyclin A disruption can potentially be attributed to incomplete DNA replication or replication stress (Mankouri et al., 2013). Furthermore, cyclin A depletion can trigger chromosomal instability during mitosis because of impaired MRE11-dependent resolution of stalled replication forks (Kanakkanthara et al., 2016). On the other hand, the depletion of cyclin A in G_2_ RPE1 cells impedes mitotic entry by hindering cyclin B-CDK1 activation, highlighting cyclin A’s role in triggering mitotic entry independently of S phase (Hégarat et al., 2020).

Although the discoveries of cyclins and CDKs are some of the most pivotal for our understanding of the cell cycle, the unique roles of individual mitotic cyclins are far from settled, particularly in cancer cell lines where cell cycle dynamics can be rewired. Recently, we found that CDK1 can substitute for all functions of CDK2 and that the multiple mitotic defects caused by CDK1 deficiency can be compensated by overexpressing CDK2 (Lau et al., 2021). We propose that the distinctions among different mitotic cyclins in human cells may similarly be quantitative. Using new degron-based tools in this study, we aim to define the capability and sufficiency of individual mitotic cyclins in driving mitosis.

## Results

### B-type cyclin deficiency results in pre-NEBD attenuation of mitosis in cancer cell lines

**Figure S1.**
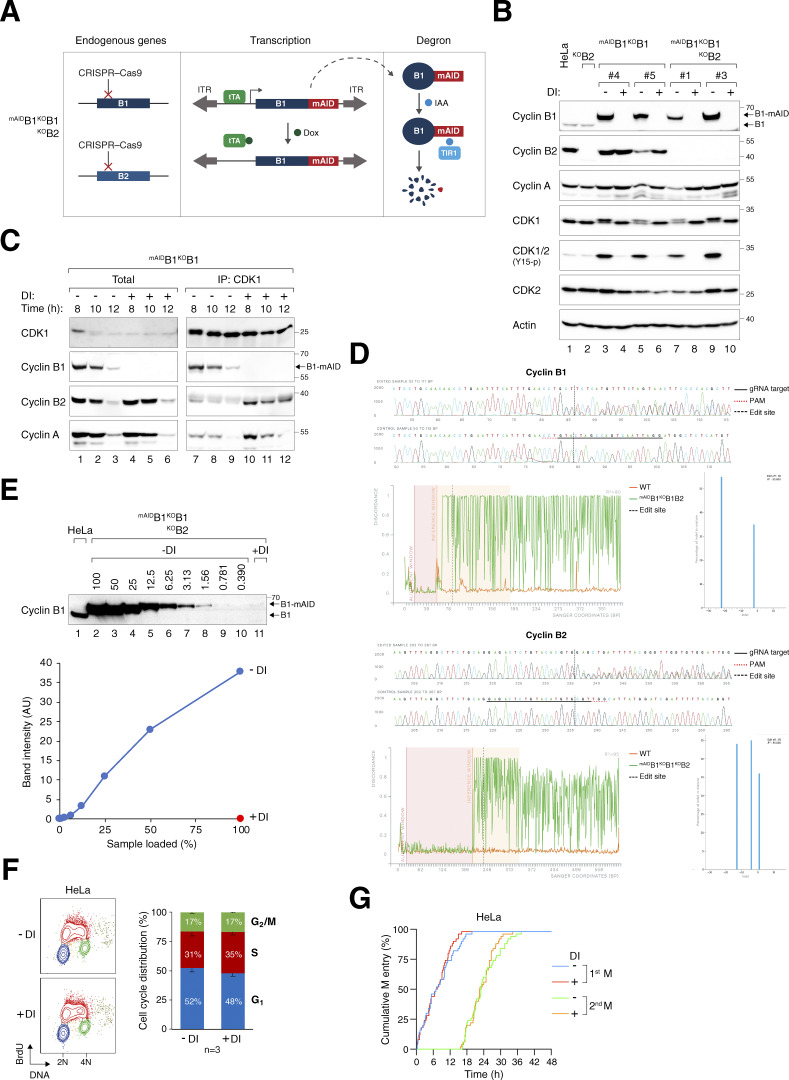
**Gene silencing of cyclin B1 and cyclin B2. (A)** Conditional gene silencing strategy for mitotic cyclin B. CRISPR-Cas9 was used to disrupt the loci of both endogenous cyclin B1 and cyclin B2. The cDNA of cyclin B1 was tagged with mAID and put inside a Sleeping Beauty transposon cassette for genome delivery to rescue the KO effects. Silent mutations were introduced into ^mAID^cyclin B1 to confer resistance to the CRISPR-Cas9. In the presence of Dox, transcription of ^mAID^cyclin B1 is inhibited by blocking the tetracycline-controlled transcriptional activator (tTA) from binding to the TRE in the promoter. The addition of IAA triggers the degradation of residual ^mAID^cyclin B1 in cells expressing the F-box protein TIR1. ITR: inverted terminal repeat. **(B)** Gene silencing of cyclin B1 and/or cyclin B2. HeLa cells were engineered to stably express ^mAID^cyclin B1, tTA, and TIR1. CRISPR–Cas9 was used to disrupt cyclin B1 (in ^mAID^B1^KO^B1) or both cyclin B1 and B2 (in ^mAID^B1^KO^B1B2). Single-colony–derived clones were isolated and cultured with or without DI for 8 h. Lysates were prepared and analyzed with immunoblotting. Lysates from parental HeLa and cyclin B2 KO cells (^KO^B2) were included as controls. Equal loading of lysates was confirmed by immunoblotting for actin. **(C)** Enhanced formation of cyclin B2–CDK1 and cyclin A–CDK1 complexes in the absence of cyclin B1. ^mAID^B1^KO^B1 cells synchronized with a double thymidine block were cultured with or without DI and harvested at the indicated time points. Lysates were prepared and subjected to immunoprecipitation with an antibody against CDK1. Both total lysates and immunoprecipitates were analyzed with immunoblotting. **(D)** Indel analysis of cyclin B1 and cyclin B2. The endogenous cyclin B1 (*CCNB1*) and cyclin B2 (*CCNB2*) loci in ^mAID^B1^KO^B1B2 cells were analyzed with sequencing. Sequencing traces of control (HeLa) and the edited samples were generated for indel analysis. The targeted sequence of the gRNA (solid black line), PAM sequence (dotted red line), and edited site (dotted black line) are indicated. Discordance, calculated by ICE, is shown for the edited (green) and control (orange) traces. The alignment window indicates the region of the traces with high Phred quality scores used for alignment. The inference window indicates the altered sequences around the edited site (dotted black line). Indel and corresponding prevalence were determined using ICE, with editing efficiencies of 90% for cyclin B1 and 95% for cyclin B2. **(E)** Efficiency of cyclin B1 silencing in ^mAID^B1^KO^B1B2 cells. After treatment with DI for 6 h, lysates were prepared and analyzed with immunoblotting. Lysates from HeLa cells were included to serve as a reference for the expression level of endogenous cyclin B1. The signals corresponding to ^mAID^cyclin B1 were quantified using a standard curve based on serial dilutions of ^mAID^B1^KO^B1B2 cell lysates (lanes 2–10), showing that <1% of ^mAID^cyclin B1 remained after DI treatment. **(F)** DI treatment does not affect the overall cell cycle distribution. HeLa cells were treated with DI for 24 h, pulsed with BrdU for 30 min, and analyzed using bivariate flow cytometry. Representative contour plots are shown (red: BrdU-positive; yellow: BrdU-negative S; blue: G_1_; green: G_2_/M). The positions of 2N and 4N DNA content are indicated. The percentage of cells at different cell cycle stage (excluding BrdU-negative S) was quantified. Mean and SEM from three independent experiments. **(G)** DI treatment does not affect cell cycle progression. Parental HeLa cells were treated with DI and analyzed using live-cell imaging for 48 h. The cumulative percentage of cells entering the first and second mitosis over time is shown. Source data are available for this figure: SourceData FS1.

**Figure 1.**
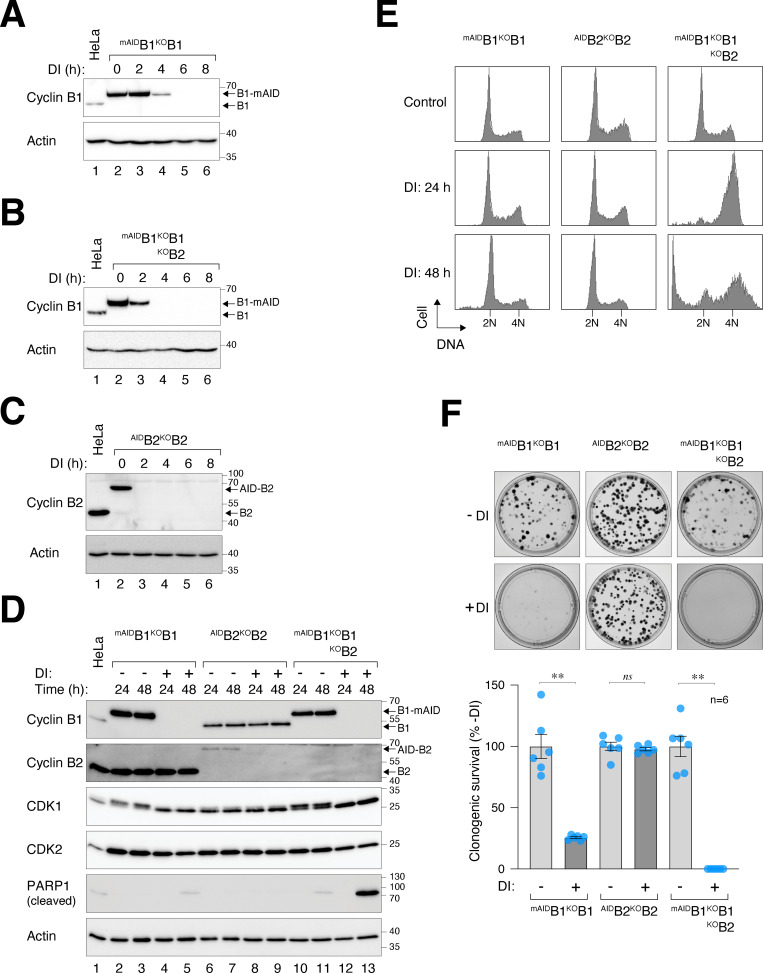
**Essential role of cyclin B1 and B2 in cell proliferation and survival. (A)** Conditional silencing of cyclin B1. HeLa cells were engineered to stably express ^mAID^cyclin B1, tTA, and TIR1, concurrently disrupting the endogenous cyclin B1 with CRISPR-Cas9. Clones of ^mAID^B1^KO^B1 cells were isolated and cultured in the presence of Dox and IAA (DI). The cells were harvested at different time points for immunoblotting analysis. Lysates from control HeLa cells were used to compare endogenous cyclin B1 levels. Equal loading of lysates was confirmed by immunoblotting for actin. **(B)** Simultaneous silencing cyclin B1 and B2. ^mAID^B1^KO^B1B2 cells were generated and treated with DI similarly as in A (see Materials and methods). **(C)** Conditional silencing cyclin B2. ^AID^B2^KO^B2 cells were generated and treated with DI similarly as in A (see Materials and methods). **(D)** Depletion of cyclin B1 and B2 promotes apoptosis. Different cell lines were cultured with or without DI and harvested at the indicated time points for immunoblotting. **(E)** Depletion of cyclin B promotes mitotic block and apoptosis. The indicated cell lines were cultured with or without DI. At different time points, the cells were fixed and analyzed with flow cytometry. Positions of 2N and 4N DNA content are indicated. **(F)** Silencing of cyclin B abrogates clonogenic survival. ^mAID^B1^KO^B1B2 cells were cultured with or without DI for 2 wk. Colonies were fixed, stained, and quantified. Mean ± SEM from six independent experiments. Mann–Whitney test: **P < 0.01; *ns* P > 0.05. Source data are available for this figure: SourceData F1.

Silencing of cyclins at specific cell cycle stages was achieved using a dual transcription–degron system (Ng et al., 2019; Yeung et al., 2021) (Fig. S1 A). Concurrent with the disruption of cyclin B1 with CRISPR-Cas9, a mini auxin-induced degron (mAID)-tagged cyclin B1, under the control of a Tet-Off promoter, was delivered to the genome using Sleeping Beauty transposase. This system allowed us to turn off the transcription of ^mAID^cyclin B1 using doxycycline (Dox) and target pre-existing ^mAID^cyclin B1 for proteolysis using indole-3-acetic acid (IAA). Single-colony-derived clones lacking endogenous cyclin B1 and expressing different levels of ^mAID^cyclin B1 were isolated (^mAID^B1^KO^B1 herein; Fig. S1 B). Upon exposure to Dox and IAA (DI herein), ^mAID^cyclin B1 was reduced to below our detection limit between 4 and 6 h (Fig. 1 A).

Although cyclin B2 is a non-essential gene in most human cell lines (https://depmap.org), the rise in cyclin B2–CDK1 complexes in the absence of cyclin B1 suggests a potential compensatory role of cyclin B2 (Fig. S1 C). We therefore applied CRISPR-Cas9 to target cyclin B2 and generated ^mAID^B1^KO^B1B2 cells (Fig. 1 B and Fig. S1 B). Genome sequencing confirmed the disruption of both *CCNB1* and *CCNB2* genes (Fig. S1 D). We also generated cyclin B2 KO cells (^KO^B2; Fig. S1 B) and those also containing ^AID^cyclin B2 (^AID^B2^KO^B2; Fig. 1 C). As KO of cyclin B2 did not affect mitosis (see later), ^AID^B2^KO^B2 cells were used as controls in this study to ensure that the AID system did not interfere with mitosis.

Degradation of ^mAID^cyclin B1 in ^mAID^B1^KO^B1B2 cells was rapid, dropping to undetectable levels within 4 h of DI treatment (Fig. 1 B). By applying a standard curve generated from serial dilutions of lysates, we estimated that <1% of ^mAID^cyclin B1 remained (Fig. S1 E). Silencing of cyclin B1 and B2 led to the accumulation of cleaved PARP1 (Fig. 1 D), an increase in sub-G_1_ apoptotic cells (Fig. 1 E), and a decrease in clonogenic survival (Fig. 1 F). Interestingly, although the loss of cyclin B1 did not immediately affect cell cycle distribution, it compromised colony size and number. It should be noted that DI treatment did not significantly affect the cell cycle in parental cells, as shown by BrdU incorporation assays and flow cytometry (Fig. S1 F), live-cell imaging (Fig. S1 G), or clonogenic survival (Yeung et al., 2023).

**Figure 2.**
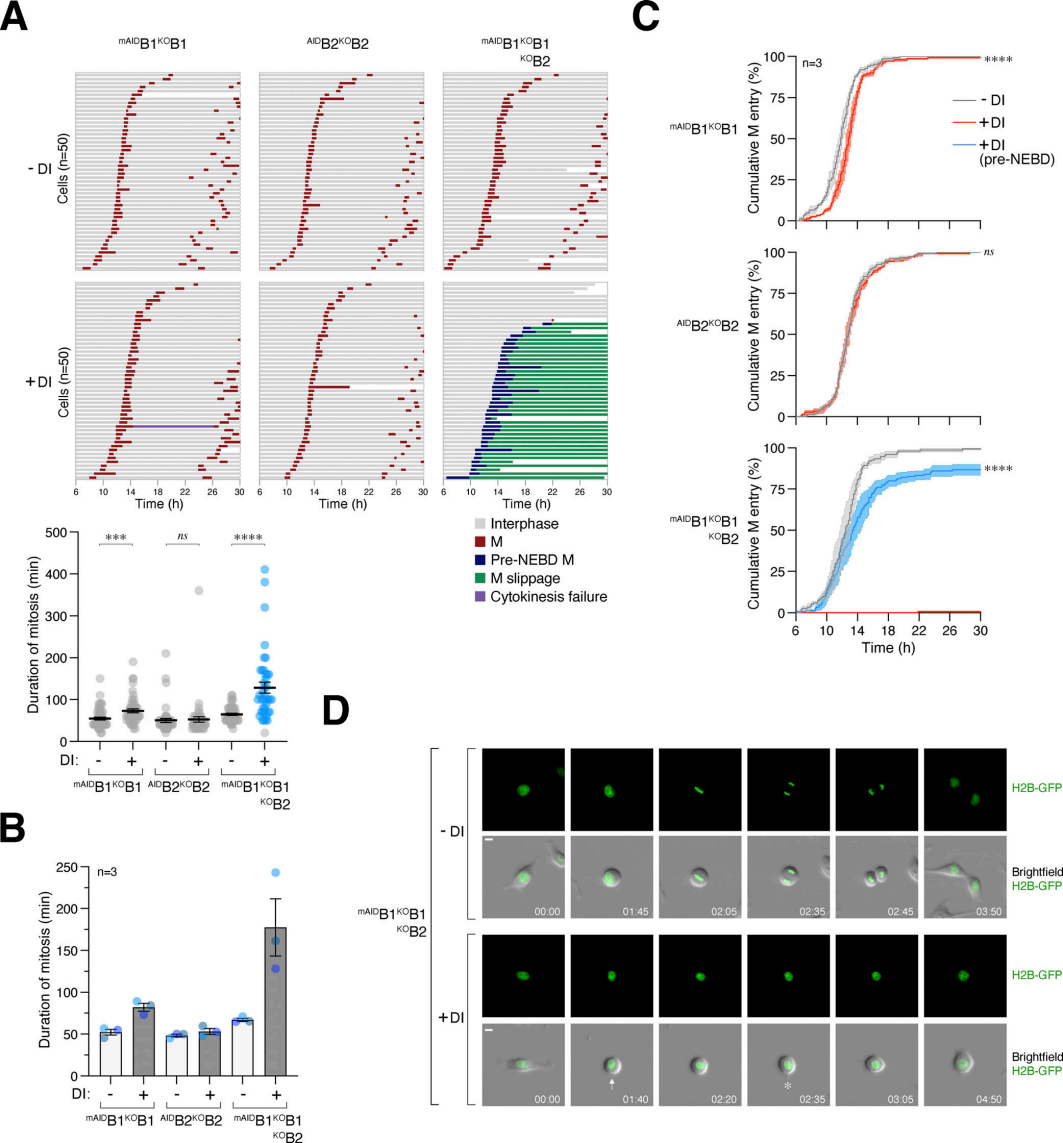
**Conditional depletion of cyclin B induces defective mitotic entry and pre-NEBD slippage. (A)** Pre-NEBD slippage in cyclin B-depleted cells. Different cell lines expressing histone H2B-GFP were synchronized using a double thymidine block and released into drug-free or DI-containing medium for 6 h to turn off cyclin B1 before time-lapse imaging. Time indicates the duration after thymidine release. Key: interphase (grey); mitosis (red); cell death (truncated bars); pre-NEBD mitosis (blue), and interphase after pre-NEBD slippage (green). The plot at the bottom shows the elapsed time between mitotic entry and exit (or cell death). For cells exhibiting pre-NEBD slippage, the time of mitosis was defined as from cell rounding to the appearance of cytoplasmic processes before cell flattening (blue). Mean ± SEM (*n* = 50). Mann–Whitney test: ****P < 0.0001; ***P < 0.001; *ns* P > 0.05. **(B)** Depletion of cyclin B1 lengthens the duration of mitosis. Live-cell imaging was performed to determine mean mitotic duration as described in A. Mean ± SEM from three independent experiments. **(C)** Depletion of cyclin B delays mitotic entry. Cell lines were synchronized using double-thymidine block and released into a drug-free or DI-containing medium. After 6 h, individual cells were tracked using live-cell imaging. The cumulative percentage of cells entering mitosis over time is shown. Note that DI-treated ^mAID^B1^KO^B1B2 cells mainly entered pre-NEBD slippage instead of normal mitosis (shown in blue). Mean ± SEM from three independent experiments. Mann–Whitney test: ****P < 0.0001; *ns* P > 0.05. **(D)** Pre-NEBD slippage induced by cyclin B silencing. Cells were imaged following the procedure outlined in A. Representative images show ^mAID^B1^KO^B1B2 cells undergoing mitosis in the presence or absence of DI. In DI-treated cells, the commencement of cell rounding and pre-NEBD slippage are indicated by the arrow and asterisk, respectively. Time: h:min. Scale bar: 10 µm. See Videos 1 and 2.

**Figure S2.**
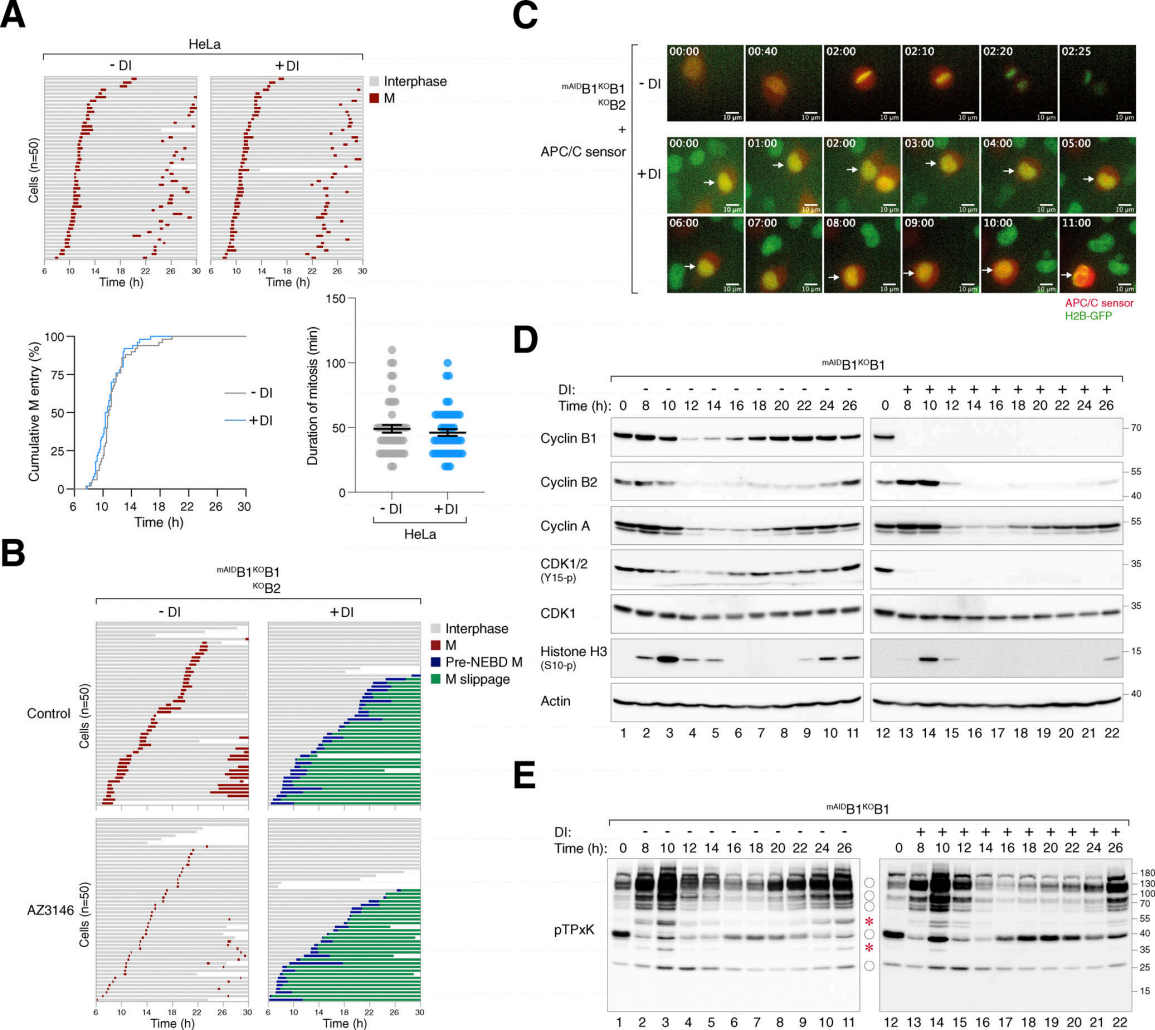
**Conditional depletion of cyclin B induces defective mitotic entry and mitotic slippage. (A)** DI treatment of control HeLa cells. HeLa cells expressing histone H2B-GFP were synchronized using a double thymidine block and released into a drug-free or DI-containing medium for 6 h before time-lapse imaging. Time indicates the duration after thymidine release. Key: interphase (grey); mitosis (red); and cell death (truncated bars). The plots show the cumulative percentage of cells entering mitosis over time and the elapsed time between mitotic entry and exit. **(B)** Defective mitosis in the absence of cyclin B does not involve SAC activation. ^mAID^B1^KO^B1B2 cells were cultured in a drug-free or DI-containing medium with or without the MPS1 inhibitor AZ3146. After 6 h, individual cells were tracked using live-cell imaging. Key: interphase (grey); mitosis (red); cell death (truncated bars); pre-NEBD mitosis (blue), and interphase after pre-NEBD slippage (green). **(C)** Silencing of cyclin B prevents APC/C activation. ^mAID^B1^KO^B1B2 cells expressing histone H2B-GFP were transfected with an mRFP APC/C biosensor plasmid. The cells were cultured in drug-free or DI-containing medium and analyzed using live-cell imaging. Representative images show normal mitosis and abnormal mitosis without APC/C activation. Time: h:min. Scale bar: 10 µm. **(D)** Loss of cyclin B1 alone does not abolish mitotic entry and exit. ^mAID^B1^KO^B1 cells were synchronized using double thymidine block and released into a drug-free or DI-containing medium. The cells were harvested at the indicated time points for immunoblotting analysis. **(E)** CDK1 substrate phosphorylation in the absence of cyclin B1. Samples from D were subjected to immunoblotting using an antibody against phosphorylated CDK1 substrates (pTPxK). The positions of bands affected by cyclin B depletion are as described in Fig. 4 B. Source data are available for this figure: SourceData FS2.

To determine the cell cycle defects associated with cyclin B deficiency, we synchronized various ^mAID^cyclin B-expressing cells with a double thymidine block procedure and released them into a normal or DI-containing medium. Live-cell imaging was then conducted to monitor individual cells (Fig. 2 A). DI-treated parental HeLa cells were used as controls (Fig. S2 A). Depletion of cyclin B1 (but not B2) in G_2_ slightly delayed mitotic entry (Fig. 2 C) and prolonged the duration of mitosis (Fig. 2, A and B), suggesting that the remaining mitotic cyclins (A and B2) could perform most functions of cyclin B1 in these cells. Intriguingly, cells lacking both cyclin B1 and B2 exhibited cell rounding concurrently with normal cells (Fig. 2 C). However, mitosis was severely curtailed, without clear signs of chromosomal condensation. Following a prolonged period of cell rounding (>160 min; compared with ∼65 min of mitosis in control cells, Fig. 2 B), the cyclin B1- and B2-deficient cells reverted to interphase (an example is shown in Fig. 2 D). This phenomenon was termed pre-NEBD slippage in this study (Videos 1, 2, 3, and 4).

**Video 1.** **Normal mitosis in control cells.** ^mAID^B1^KO^B1B2 cells expressing histone H2B-GFP were synchronized using a double thymidine block and released into a drug-free medium before time-lapse imaging. A representative cell undergoing normal mitosis is shown. Time: h:min.

**Video 2.** **Pre-NEBD slippage in cyclin B-deficient cells.** ^mAID^B1^KO^B1B2 cells expressing histone H2B-GFP were synchronized using a double thymidine block and released into a DI-containing medium before time-lapse imaging. A representative cell undergoing pre-NEBD mitosis and slippage is shown. Time: h:min.

**Video 3.** **NEBD during normal mitosis.** ^mAID^B1^KO^B1B2 cells were transfected with a plasmid expressing mRFP-lamin A and cultured in a drug-free medium. A representative cell undergoing normal mitosis is shown. Time: h:min.

**Video 4.** **Absence of NEBD in cyclin B-depleted cells.** ^mAID^B1^KO^B1B2 cells were transfected with a plasmid expressing mRFP-lamin A and cultured in a DI-containing medium. A representative cell undergoing pre-NEBD mitosis and slippage is shown. Time: h:min.

**Figure 3.**
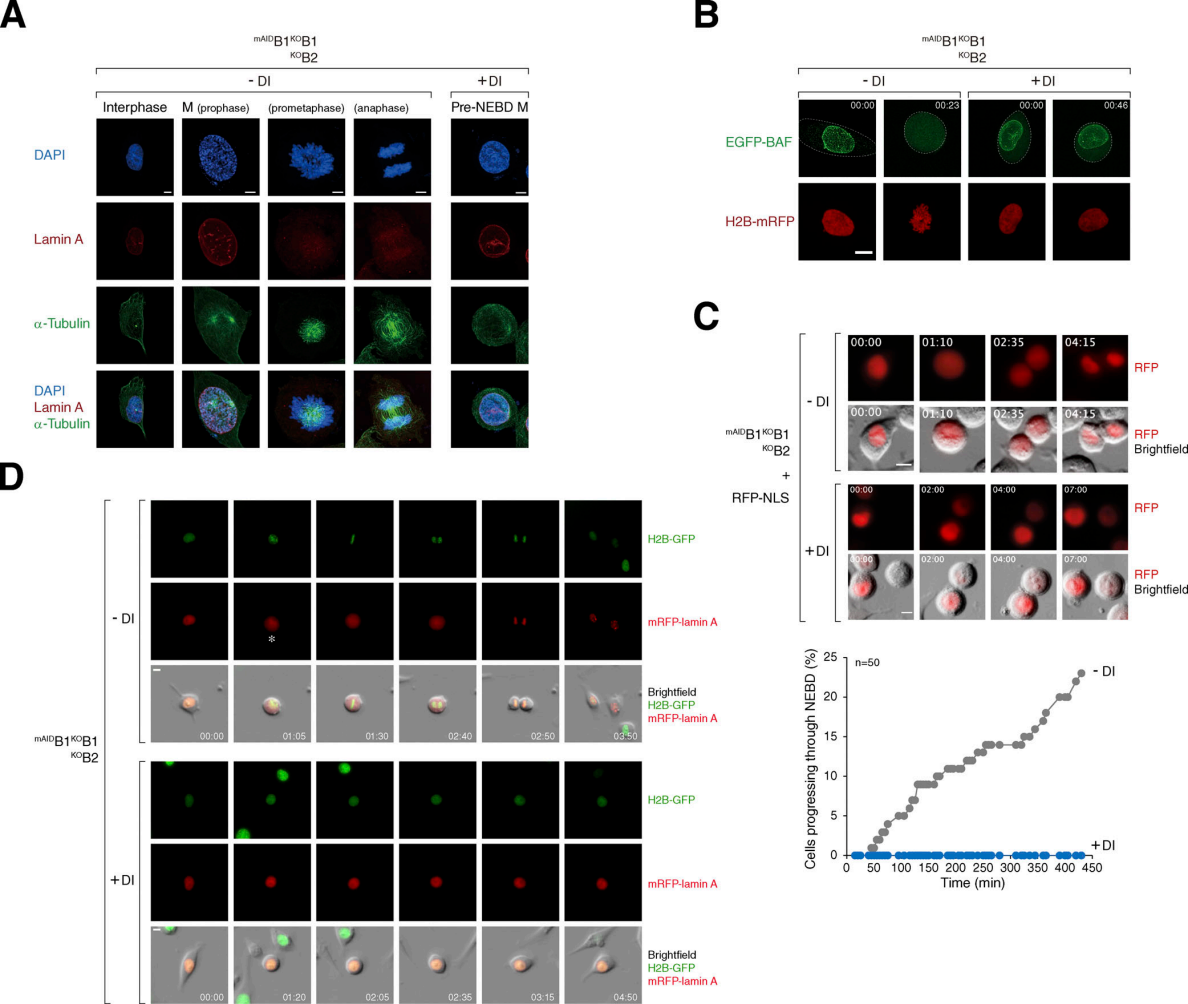
**Depletion of cyclin B induces pre-NEBD slippage. (A)** Abnormal mitosis with intact lamin A coupled with cytoskeleton rearrangement in cyclin B-depleted cells. ^mAID^B1^KO^B1B2 cells were synchronized using a double thymidine block and released into a drug-free or DI-containing medium. After 12 h, cells were fixed and imaged using Airyscan confocal microscopy. Representative images show untreated cells in interphase or mitosis and a DI-treated cell undergoing aberrant mitosis. Scale bar: 5 µm. **(B)** Depletion of cyclin B leads to mitosis devoid of NEBD. ^mAID^B1^KO^B1B2 cells were transfected with plasmids expressing EGFP-BAF and histone H2B-EGFP. After 36 h, the cells were synchronized using a single thymidine block and released into either a drug-free or DI-containing medium. After 10 h, live-cell imaging was performed using Airyscan confocal microscopy. Representative images of a control cell entering mitosis (note the DNA condensation and the redistribution of EGFP-BAF) and a cyclin B-depleted cell lacking breakdown of EGFP-BAF-containing nuclear lamina are shown. Cell outlines are indicated by white dotted lines. Time: h:min. Scale bar: 10 µm. **(C)** Silencing of cyclin B results in mitosis without loss of nuclear membrane integrity. ^mAID^B1^KO^B1B2 cells were transfected with an RFP-NLS expression plasmid, cultured in drug-free or DI-containing medium, and analyzed using live-cell imaging. Representative images show normal mitosis and aberrant mitosis without NEBD. Time: h:min. Scale bar: 10 µm. The graph represents the cumulative percentage of cells that have progressed past NEBD, as judged by the flooding of RFP-NLS signal (*n* = 50). **(D)** Absence of NEBD in cyclin B-depleted cells. ^mAID^B1^KO^B1B2 cells were transfected with a plasmid expressing mRFP-lamin A and cultured in a drug-free or DI-containing medium. Representative images from live-cell imaging analysis of normal mitosis and abnormal mitosis are shown (NEBD denoted by an asterisk). Time: h:min. Scale bar: 10 µm. See Videos 3 and 4.

Cell rounding in cyclin B-deficient cells occurred without NEBD, as indicated by lamin A staining of the nuclear lamina (Fig. 3 A). Live-cell confocal imaging revealed the lack of DNA-binding barrier-to-autointegration factor (BAF) (Jamin and Wiebe, 2015) hyperaccumulation in the nuclei of rounded-up cells, confirming that cyclin B-deficient cells maintained an intact nuclear envelope (Fig. 3 B). The absence of NEBD in cyclin B-deficient cells was further supported by the exclusive nuclear signal of a nuclear localization sequence (NLS)-tagged RFP reporter (Fig. 3 C). To further validate the absence of NEBD, ^mAID^B1^KO^B1B2 cells were transfected with a plasmid expressing mRFP-tagged lamin A. During normal mitosis, lamin A redistributed from the nucleus to the entire cell, whereas in rounded-up cyclin B-deficient cells, lamin A remained confined to the nucleus (Fig. 3 D).

Silencing of the spindle assembly checkpoint (SAC) with an MPS1 inhibitor (AZ3146) shortened the duration of unperturbed mitosis in normal cells (Hewitt et al., 2010). However, AZ3146 did not affect the timing of pre-NEBD slippage in cyclin B-deficient cells (Fig. S2 B), indicating the absence of SAC activation. We also used an APC/C reporter (mRFP fused to cyclin B1’s D-box) to confirm that while APC/C was activated during anaphase in normal cells, it remained inactive throughout the cell rounding and pre-NEBD slippage in cyclin B-deficient cells (Fig. S2 C).

These results demonstrate that cyclin B1 and B2 deficiency results in defective mitosis characterized by cell rounding and the absence of NEBD, DNA condensation, or complete APC/C activation.

### Depletion of cyclin B1 and B2 uncouples the G_2_–M kinase network from mitosis

**Figure 4.**
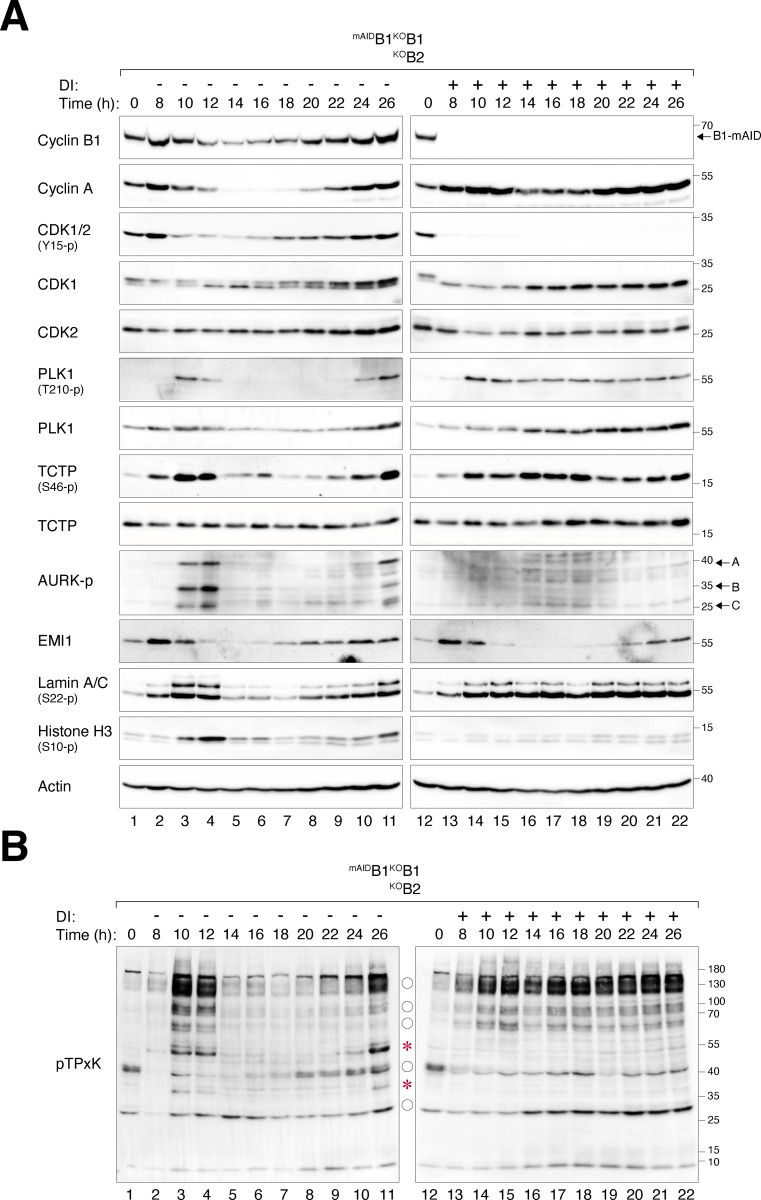
**Loss of cyclin B uncouples the normal regulation of the G**_**2**_ **-M kinase network. (A)** Dysregulated phosphorylation and expression of G_2_–M regulators in the absence of cyclin B. ^mAID^B1^KO^B1B2 cells synchronized using double thymidine block were released into a drug-free or DI-containing medium and harvested at different time points for immunoblotting. The positions of the three isoforms of Aurora kinases are indicated. **(B)** Dysregulation of CDK1 substrate phosphorylation in the absence of cyclin B. Samples prepared from A were immunoblotted with an antibody against CDK1 phosphorylation substrates (pTPxK). The positions of bands that are absent in DI-treated cells are indicated with asterisks. Bands present in both DI-treated and untreated cells but lacking cell cycle variation in DI-treated samples are indicated with circles. Source data are available for this figure: SourceData F4.

We next examined the expression of key G_2_–M players in synchronized cyclin B-deficient cells. Transient phosphorylation of histone H3^Ser10^, Aurora kinases, PLK1, and TCTP^S46^ (a PLK1 substrate) confirmed that mitotic entry and exit were executed normally in untreated ^mAID^B1^KO^B1B2 cells (Fig. 4 A). By contrast, DI-treated ^mAID^B1^KO^B1B2 cells showed impaired phosphorylation of histone H3^Ser10^ and Aurora kinases. Furthermore, the normal transient activation of PLK1 was replaced by a protracted activation in cyclin B-deficient cells. Consistently, sustained phosphorylation of TCTP^S46^ was detected in the absence of cyclin B. These results indicate a distinct phosphorylation environment during cyclin B-deficient mitosis.

In the absence of cyclin B, the normal transient accumulation and subsequent degradation of cyclin A were impaired, resulting in an overall accumulation of cyclin A (Fig. 4 A). The reduction in cyclin A at *t* = 14 h coincided with the time of pre-NEBD slippage (Fig. 2 A), suggesting a possibility for partial or transient APC/C activation. Despite the increased cyclin A levels, the phosphorylation of CDK1^Y15^ during G_2_–M was abolished.

In cells lacking cyclin B1 but still containing cyclin B2 (^mAID^B1^KO^B1), both the phosphorylation of histone H3^Ser10^ and the degradation of cyclin A and cyclin B2 were relatively normal (Fig. S2 D), consistent with the results obtained from live-cell imaging that cyclin B2 was sufficient for promoting mitosis (Fig. 2, A and B).

To assess the impact of cyclin B deficiency on CDK1 activity, we examined the phosphorylation of substrates including lamin A/C. Unlike the transient phosphorylation observed during normal mitosis, sustained lamin A/C^S22^ phosphorylation was detected after pre-NEBD slippage in cyclin B-deficient cells (Fig. 4 A). Using an antibody that recognizes the pTPxK motif in CDK1 substrates, we found that several proteins recognized by the antibody were strongly phosphorylated during normal mitosis. However, a subset of these proteins was absent in DI-treated ^mAID^B1^KO^B1B2 cells, suggesting that they may be specific substrates of cyclin B that cannot be phosphorylated by cyclin A–CDK1 (Fig. 4 B, asterisks). Other proteins recognized by the pTPxK antibody were present in both untreated and DI-treated samples. However, while their phosphorylation oscillated during normal mitosis, they became phosphorylated continuously in the absence of cyclin B (Fig. 4 B, circles). Finally, pTPxK proteins showed similar phosphorylation patterns in ^mAID^B1^KO^B1 cells with or without DI, suggesting that cyclin B2 (along with cyclin A) can phosphorylate these proteins in the absence of cyclin B1 (Fig. S2 E).

Collectively, these results indicate that cyclin B-deficient mitosis is associated with an anomalous G_2_–M kinase network, characterized by a suppression of Aurora kinase activity and prolonged activation of PLK1 and cyclin A–CDK1.

### Cyclin A is responsible for the residual mitotic activity in the absence of B-type cyclins

**Figure 5.**
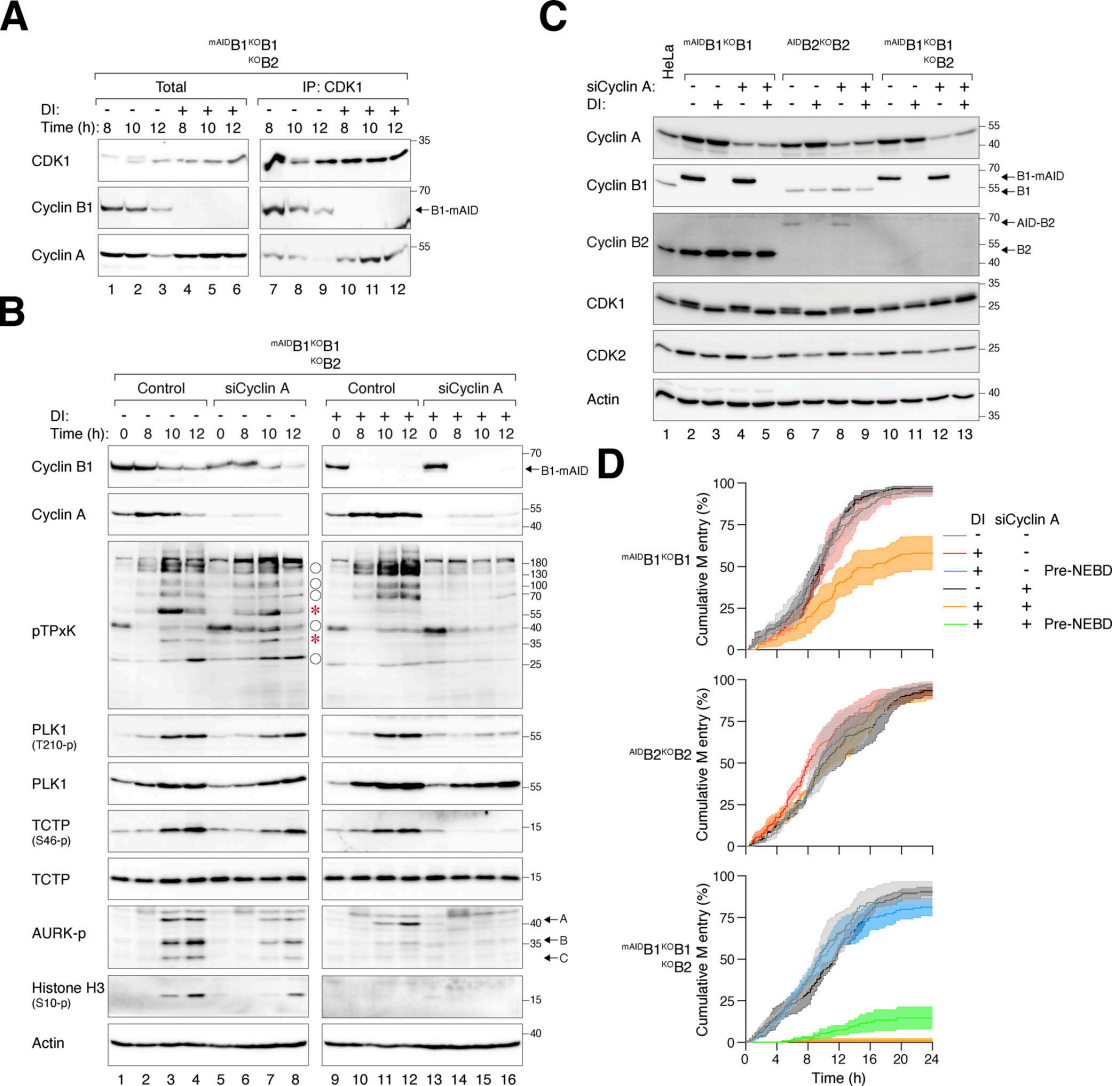
**Cyclin A drives residual mitotic activity in the absence of cyclin B. (A)** Enhanced formation of cyclin A–CDK1 complexes without cyclin B. ^mAID^B1^KO^B1B2 cells synchronized with double thymidine block were released into a drug-free or DI-containing medium and harvested at different time points. Lysates were prepared and subjected to immunoprecipitation with a CDK1 antibody. Both total lysates and immunoprecipitates (IP) were analyzed with immunoblotting. **(B)** Contribution of cyclin A to CDK1 substrate phosphorylation in the absence of cyclin B. ^mAID^B1^KO^B1B2 cells transfected with control siRNA (siControl) or siRNA targeting cyclin A (siCyclin A) were synchronized using double thymidine block. The cells were released into a drug-free or DI-containing medium and harvested at different time points for immunoblotting. The positions of pTPxK bands affected by cyclin B depletion are indicated as described in Fig. 4 B. **(C)** Depletion of cyclin A does not affect cyclin B expression. Cell lines transfected with control siRNA or siRNA targeting cyclin A were left untreated or treated with DI for 24 h. Lysates were prepared and analyzed with immunoblotting. **(D)** Suppression of mitotic entry in cyclin B-deficient cells upon cyclin A knockdown. Cell lines transfected with control siRNA or siRNA targeting cyclin A were analyzed using live-cell imaging after treatment with DI. The cumulative percentage of cells entering mitosis over time is shown. Note that DI-treated ^mAID^B1^KO^B1B2 cells exhibited pre-NEBD slippage (*) instead of normal mitosis. Source data are available for this figure: SourceData F5.

The increase in cyclin A–CDK1 complexes following the degradation of ^mAID^cyclin B1 in cells without cyclin B1 (Fig. S1 C) or without both cyclin B1 and B2 (Fig. 5 A) suggests that cyclin A–CDK1 complexes may phosphorylate some of the original cyclin B–CDK1 substrates. Depletion of cyclin A in cyclin B1-containing cells using siRNA resulted in a delay in mitotic entry (Fig. 5 B). Densitometry analysis revealed ∼80% depletion by the siRNA (Fig. 5 C). Downregulation of cyclin A resulted in a mild delay in mitotic entry in cells lacking cyclin B1 or B2 individually (Fig. 5 D). However, in the absence of both cyclin B1 and B2, depletion of cyclin A inhibited the phosphorylation of several pTPxK CDK1 substrates (Fig. 5 B), suggesting that they were phosphorylated by cyclin A–CDK1 under cyclin B-deficient conditions. Cyclin A depletion also prevented PLK1 activation (PLK1^T210^ and TCTP^S46^ phosphorylation) in cyclin B-deficient cells. Consistent with these molecular changes, live-cell imaging revealed that cyclin A-depleted cyclin B-deficient cells were unable to enter mitosis, including pre-NEBD cell rounding (Fig. 5 D).

These data highlight the role of cyclin A in driving entry into pre-NEBD mitosis in the absence of cyclin B.

### Cyclin A can quantitatively overcome B-type cyclin deficiency

**Figure S3.**
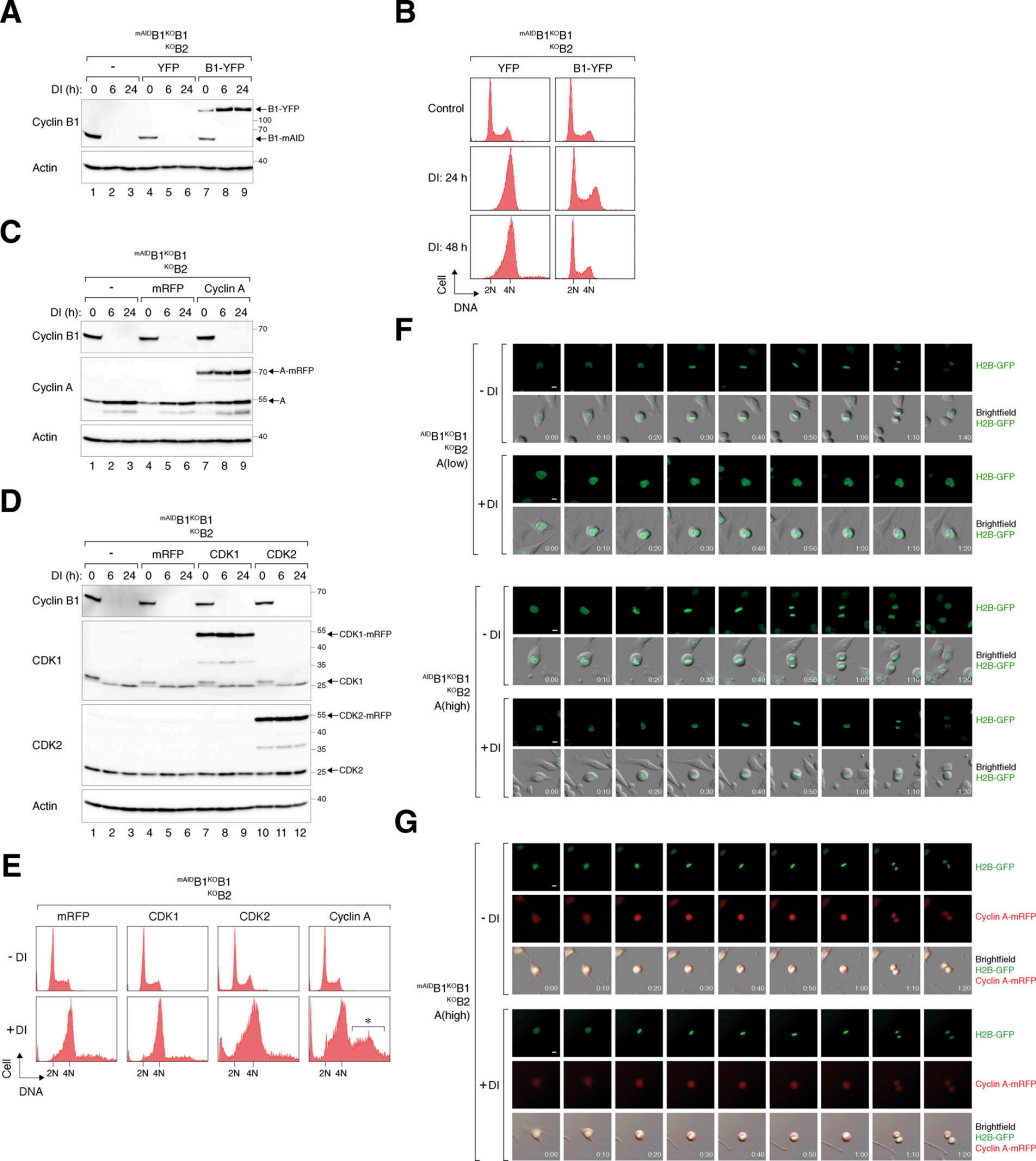
**Rescue of cell cycle defects caused by cyclin B deficiency with cyclin B1 and cyclin A. (A)** Expression of cyclin B1-YFP in cyclin B-deficient cells. ^mAID^B1^KO^B1B2 cells were transfected with a control plasmid or plasmids expressing YFP or cyclin B1-YFP. At 16 h after transfection, the cells were treated with DI and harvested at different time points. Lysates were prepared and analyzed with immunoblotting. **(B)** Ectopic expression of cyclin B1 rescues cell cycle defects induced by cyclin B deficiency. ^mAID^B1^KO^B1B2 cells transfected with plasmids expressing YFP or cyclin B1-YFP were treated with DI and harvested at the indicated time points for flow cytometry analysis. The DNA profiles of transfected YFP-positive cells are shown. **(C)** Overexpression of cyclin A in cyclin B-deficient cells. ^mAID^B1^KO^B1B2 cells expressing either mRFP or cyclin A-mRFP were treated with DI and harvested at specific time points. Lysates were prepared and analyzed with immunoblotting. **(D)** Overexpression of CDK1 and CDK2 in cyclin B-deficient cells. ^mAID^B1^KO^B1B2 cells expressing mRFP, CDK1-mRFP, or CDK2-mRFP were treated with DI and harvested at specific time points for immunoblotting analysis. **(E)** Ectopic expression of cyclin A promotes DNA re-replication in cyclin B-deficient cells. ^mAID^B1^KO^B1B2 cells were transfected with plasmids expressing mRFP or mRFP-tagged CDK1, CDK2, or cyclin A, followed by treatment with buffer or DI for 48 h. The cells were harvested and analyzed with flow cytometry. The DNA profiles of transfected mRFP-positive cells are shown. The asterisk indicates the population containing >4N DNA content. **(F)** Doubling cyclin A expression is insufficient to restore normal mitosis in cyclin B-deficient cells. ^mAID^B1^KO^B1B2 cells expressing “low” level of cyclin A and “high” level of cyclin A (see Fig. 6 A) were treated and imaged as described in Fig. 6 D. Representative images show mitosis in the presence and absence of DI. Time: h:min. Scale bar: 10 µm. **(G)** Localization of cyclin A-mRFP to both nucleus and cytoplasm during interphase. ^mAID^B1^KO^B1B2 cells expressing “high” level of cyclin A were treated and imaged as described in Fig. 6 D. Representative images show mitosis in the presence and absence of DI. Time: h:min. Scale bar: 10 µm. Source data are available for this figure: SourceData FS3.

**Figure 6.**
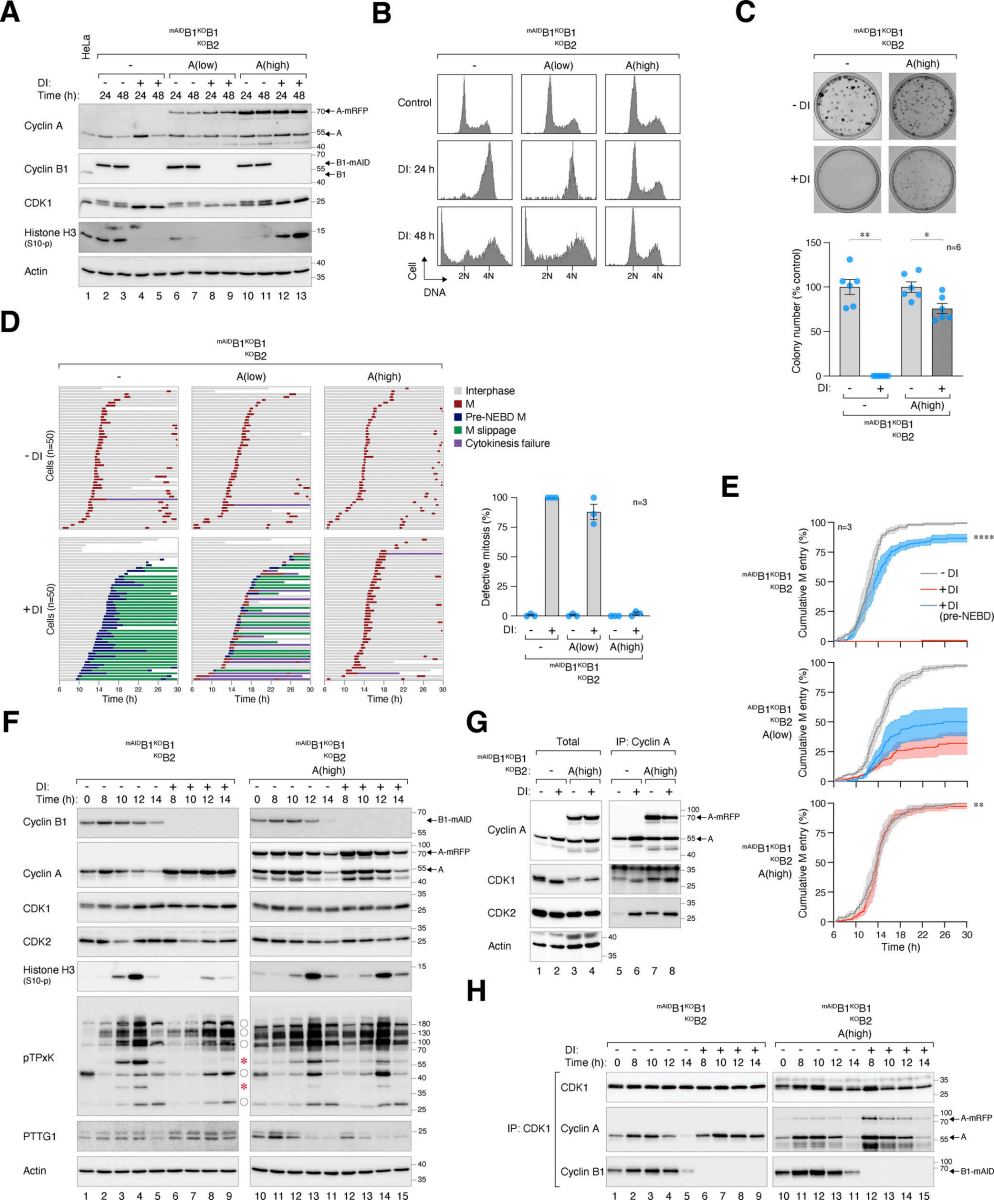
**Mitotic defects caused by cyclin B deficiency can be compensated by cyclin A overexpression. (A)** Ectopic expression of cyclin A in cyclin B-deficient cells. ^mAID^B1^KO^B1B2 cells were transfected with ^mRFP^cyclin A expression plasmids. Cells with varying levels of ^mRFP^cyclin A were sorted by flow cytometry. The cell lines were left untreated or treated with DI for the indicated time before analyzed with immunoblotting. **(B)** Rescue of cyclin B deficiency-induced G_2_/M arrest by cyclin A overexpression. Parental and ^mAID^B1^KO^B1B2 cells expressing low or high levels of ^mRFP^cyclin A were treated with DI to turn off ^mAID^cyclin B1. The cells were harvested at the indicated time points and analyzed using flow cytometry. **(C)** Cyclin A rescues clonogenic survival in cyclin B-deficient cells. ^mAID^B1^KO^B1B2 cells expressing high levels of ^mRFP^cyclin A were cultured with or without DI for 2 wk. Colonies were fixed and stained. Mean ± SEM from six independent experiments. Mann–Whitney test: **P < 0.01; *P < 0.05. Note that the same data for the ^mAID^B1^KO^B1B2 control cells as in Fig. 1 F were used for comparison. **(D)** Cyclin B deficiency-induced pre-NEBD slippage can be overcome by cyclin A overexpression. Cells expressing histone H2B-GFP were synchronized using a double thymidine block and released into a drug-free or DI-containing medium. After 6 h, individual cells were tracked using live-cell imaging. Time indicates the duration after thymidine release. Key: interphase (grey); mitosis (red); cell death (truncated bars); interphase after cytokinesis failure (purple); pre-NEBD mitosis (blue), and interphase after pre-NEBD slippage (green). The plot shows the percentage of defective mitosis (mitotic slippage and cytokinesis failure). Mean ± SEM from three independent experiments. **(E)** Overexpression of cyclin A overcomes mitotic entry delay in cyclin B-deficient cells. Cell lines were synchronized, released into drug-free or DI-containing medium, and analyzed with live-cell imaging as described in D. The cumulative percentage of cells entering mitosis (both normal and pre-NEBD mitosis) over time is shown. Mean ± SEM from three independent experiments. Mann–Whitney test: ****P < 0.0001; ***P < 0.001; *ns* P > 0.05. Note that the same graph from Fig. 2 C is included for clarity for control ^mAID^B1^KO^B1B2 cells. **(F)** Ectopic expression of cyclin A restores mitosis in cyclin B-deficient cells. Parental and cyclin A-overexpressing ^mAID^B1^KO^B1B2 cells were synchronized using a double thymidine block. The cells were released into a drug-free or DI-containing medium and harvested at different time points. Protein expression was analyzed with immunoblotting. The positions of pTPxK bands affected by cyclin B depletion are indicated as described in Fig. 4 B. **(G)** Increased cyclin A–CDK1/2 complexes in the absence of cyclin B. Parental and cyclin A-overexpressing ^mAID^B1^KO^B1B2 cells were grown in drug-free or DI-containing medium for 24 h. Lysates were prepared and subjected to immunoprecipitation using an antibody against cyclin A. Both total lysates and immunoprecipitates (IP) were analyzed with immunoblotting. **(H)** Increased binding of both endogenous and ^mRFP^cyclin A to CDK1 upon the loss of cyclin B. Parental and cyclin A-overexpressing ^mAID^B1^KO^B1B2 cells were synchronized using double thymidine block as described in F. Lysates were prepared and subjected to immunoprecipitation using an antibody against CDK1. Source data are available for this figure: SourceData F6.

Since endogenous cyclin A alone cannot initiate NEBD without B-type cyclins, we interrogated whether increasing the expression of cyclin A could compensate for the absence of cyclin B. As a control, transiently transfected cyclin B1-YFP successfully reversed the cell cycle defects caused by cyclin B silencing (Fig. S3, A and B). We next performed similar experiments using cyclin A, CDK1, and CDK2 (Fig. S3, C and D). Notably, cyclin A overexpression promoted DNA re-replication in cyclin B-silenced cells (Fig. S3 E). To selectively increase cyclin A expression without perturbing the normal S phase control, we used flow cytometry to select cells stably expressing ^mRFP^cyclin A at similar or higher levels compared with endogenous cyclin A (Fig. 6 A). Densitometry analysis revealed that the two clones expressed cyclin A (^mRFP^cyclin A and endogenous cyclin A together) at two- and threefold above endogenous cyclin A, respectively. While moderate levels of cyclin A did not correct the increase in 4N DNA content upon cyclin B depletion, higher levels of cyclin A restored the normal cell cycle profile (Fig. 6 B). Moreover, exogenous cyclin A was able to rescue clonogenic survival in cyclin B-deficient cells, albeit with smaller colony sizes (Fig. 6 C).

Synchronized ^mAID^B1^KO^B1B2 cells expressing different levels of ^mRFP^cyclin A were then tracked using live-cell imaging (Fig. 6 D). While a twofold increase in cyclin A only mildly corrected the defective mitosis in cyclin B-deficient cells, a further increase in cyclin A expression restored timely mitotic entry and exit (Fig. 6, D and E; examples of individual cells are shown in Fig. S3 F). During interphase, ^mRFP^cyclin A was detected in both the nucleus and cytoplasm (Fig. S3 G), consistent with the known shuttling of cyclin A between these compartments (Jackman et al., 2002). Cyclin A also restored the normal periodic phosphorylation of histone H3^S10^ and pTPxK, and accumulation and destruction of securin (PTTG1), implying that cells were able to progress through anaphase (Fig. 6 F). Cyclin A also reversed the accumulation of endogenous cyclin A, providing further evidence that an elevated threshold level of cyclin A was necessary for driving mitosis without cyclin B.

As shown above (Fig. 5 A), depletion of cyclin B led to an increase in cyclin A–CDK1/2 complexes. Cyclin A–CDK1/2 complexes were further increased in cells overexpressing cyclin A (Fig. 6 G). The reciprocal immunoprecipitation demonstrated that both endogenous cyclin A and ^mRFP^cyclin A exhibited enhanced association with CDK1 upon the loss of cyclin B (Fig. 6 H).

Collectively, our data indicate that the lack of proper mitotic entry in the absence of cyclin B1 and B2 can be compensated by simply elevating cyclin A to threefold above the endogenous level, allowing cells to survive without B-type cyclins.

### Non-canonical cyclin B1–CDK2 can replace cyclin A for mitosis

**Figure 7.**
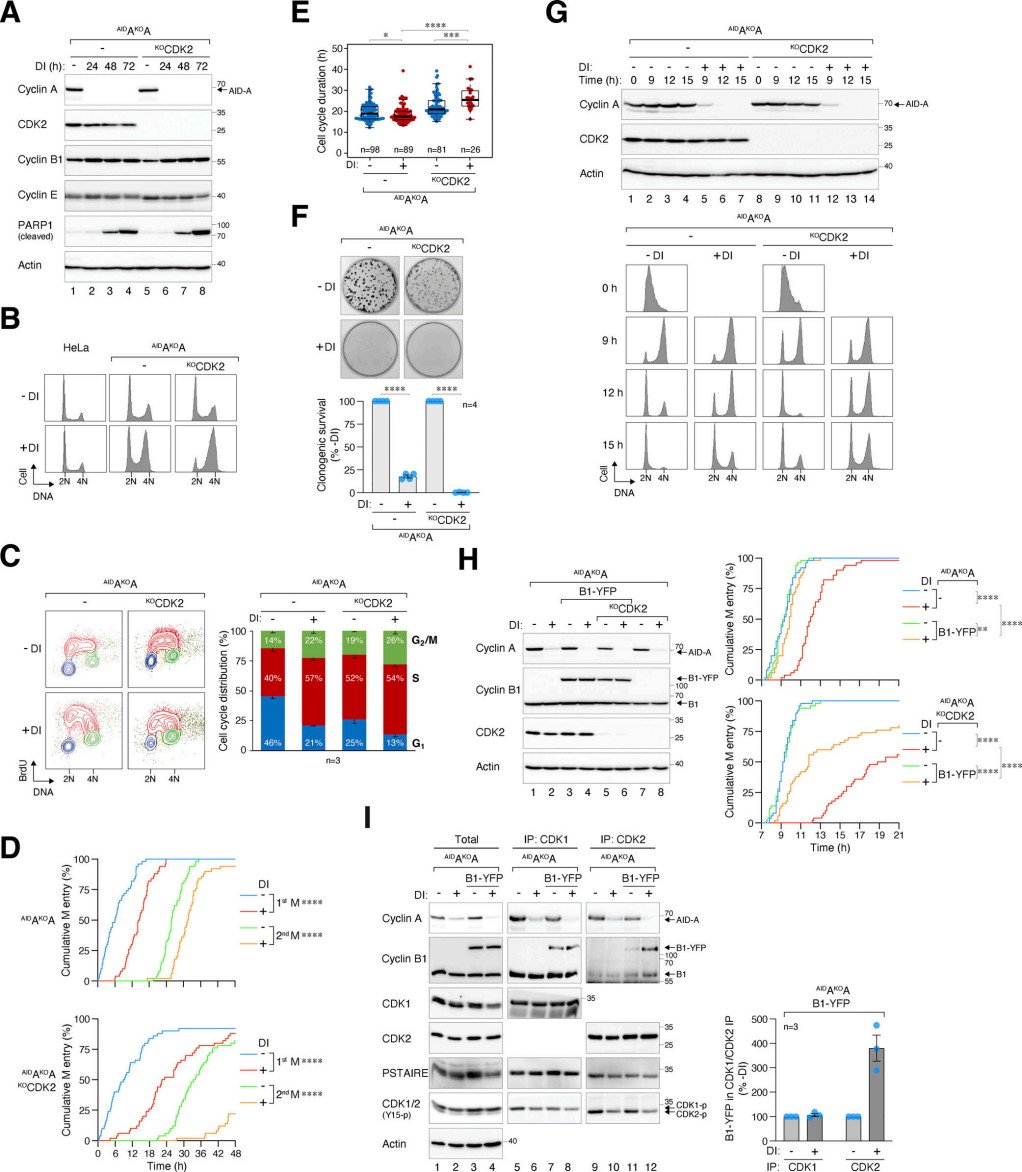
**Cyclin B1 overexpression overcomes cyclin A** ^**KO**^ **-mediated G**_**2**_ **-M delay. (A)** Conditional gene silencing of cyclin A. HeLa cells expressing ^AID^cyclin A without endogenous cyclin A were generated (^AID^A^KO^A). CDK2 was further disrupted in ^AID^A^KO^A cells using CRISPR-Cas9. The cells were either left untreated or treated with DI for the indicated time for immunoblotting analysis. **(B)** Codepletion of cyclin A and CDK2 leads to extensive G_2_/M delay. HeLa or ^AID^A^KO^A cells with or without CDK2 were either left untreated or treated with DI for 24 h. Cell cycle distribution was analyzed with flow cytometry. **(C)** Loss of cyclin A results in cell cycle delay in both S and G_2_/M. Cells were treated with DI for 24 h and pulsed with BrdU (30 min) before being analyzed with bivariate flow cytometry. Representative contour plots are shown (red: BrdU-positive; yellow: BrdU-negative S; blue: G_1_; green: G_2_/M). The percentage of cells at different cell cycle stages (excluding BrdU-negative S) was quantified (mean and SEM from three independent experiments). **(D)** Significant cell cycle delay in the absence of cyclin A and CDK2. Cells were preincubated with DI for 6 h to deplete cyclin A before individual cells were tracked using live-cell imaging for 48 h. The cumulative percentage of cells entering the first and second mitosis over time is shown (raw data for individual cells are presented in Fig. S4 A). ****P < 0.0001. **(E)** Loss of cyclin A–CDK2 delays cell cycle progression. Cells were subjected to live-cell imaging analysis as described in D. Box-and-whisker plots show the elapsed time between the end of the first mitosis to the end of the second mitosis. ****P < 0.0001; ***P < 0.001; *P < 0.05. **(F)** Cyclin A is an essential gene in HeLa cells. ^AID^A^KO^A and ^AID^A^KO^A^KO^CDK2 cells were cultured with or without DI. After 2 wk, the cells were fixed, stained with crystal violet, and the number of colonies was quantified. Representative images and Mean ± SEM from four independent experiments are shown. ****P < 0.0001. **(G)** Simultaneous cyclin A and CDK2 depletion during G_2_-M. Cells were synchronized using double thymidine block, released into drug-free or DI-containing medium, and harvested at different time points for immunoblotting analysis (upper panel). DNA content was analyzed using flow cytometry (lower panel). **(H)** Alleviation of cyclin A^KO^-induced G_2_-M delay by cyclin B1 requires CDK2. A stable cell line expressing YFP-tagged cyclin B1 (B1-YFP) was established from ^AID^A^KO^A cells. CDK2 was further disrupted to obtain ^AID^A^KO^A^KO^CDK2 cells expressing B1-YFP. Following double thymidine synchronization, cells were left untreated or treated with DI for 7 h to before analyzed using time-lapse imaging. Separate plates of cells were harvested at 3 h after the start of live-cell imaging for immunoblotting analysis to confirm protein expression. Raw data for individual cells are presented in Fig. S4 C. ****P < 0.0001; **P < 0.01. **(I)** Enhanced interaction between cyclin B1 and CDK2 in the absence of cyclin A. ^AID^A^KO^A cells, with or without ectopically expressed cyclin B1, were synchronized in G_2_ using a double thymidine block. Lysates were prepared and subjected to immunoprecipitation using antibodies against CDK1 or CDK2. The total lysates and immunoprecipitates (IP) were analyzed using immunoblotting. Note that the PSTAIRE antibody recognizes both CDK1 and CDK2. The band intensities of cyclin B1-YFP in the IP were quantified and normalized to -DI (mean ± SEM from three independent experiments). Source data are available for this figure: SourceData F7.

Given the plasticity between cyclin A and B as revealed using cyclin B-deficient cells, we conducted reciprocal experiments using cyclin A KO cell lines expressing ^AID^cyclin A (^AID^A^KO^A) (Fig. 7 A). BrdU incorporation and cell cycle analyses revealed that cyclin A-deficient cells were enriched in late S and G_2_/M (Fig. 7, B and C). Notably, the cell cycle delay upon cyclin A silencing was relatively modest compared with cyclin B depletion. Since cyclin A is known to bind to CDK2 in interphase, the lack of cell cycle delay could potentially be explained by a compensatory mechanism involving cyclin B-CDK2 in these cells. To test this hypothesis, we knocked out CDK2 and found that the additional deletion of CDK2 further delayed G_2_–M in cyclin A-deficient cells (Fig. 7, A–C). It is important to highlight that CDK2 deletion alone does not impede G_2_–M progression in HeLa cells, although it leads to the formation of smaller colonies compared with wild-type cells (Lau et al., 2021).

Live-cell imaging revealed that mitotic entry was delayed in the absence of cyclin A: the time required for 50% of cells to enter the first mitosis was delayed from ∼6 to 12 h (Fig. 7 D). This was further delayed to ∼24 h in the absence of both cyclin A and CDK2. Interestingly, the overall cell cycle duration (measured from the first to the second mitosis) was not significantly extended following the depletion of cyclin A, likely due to the accelerated entry into the second mitosis (Fig. 7, D and E). This suggests the possibility of rapid adaptive changes in the cell cycle machinery following the loss of cyclin A. Despite the compensation, long-term colony survival was significantly reduced in the absence of cyclin A (Fig. 7 F). This aligns with the accumulation of cleaved PARP1 after DI treatment (Fig. 7 A).

**Figure S4.**
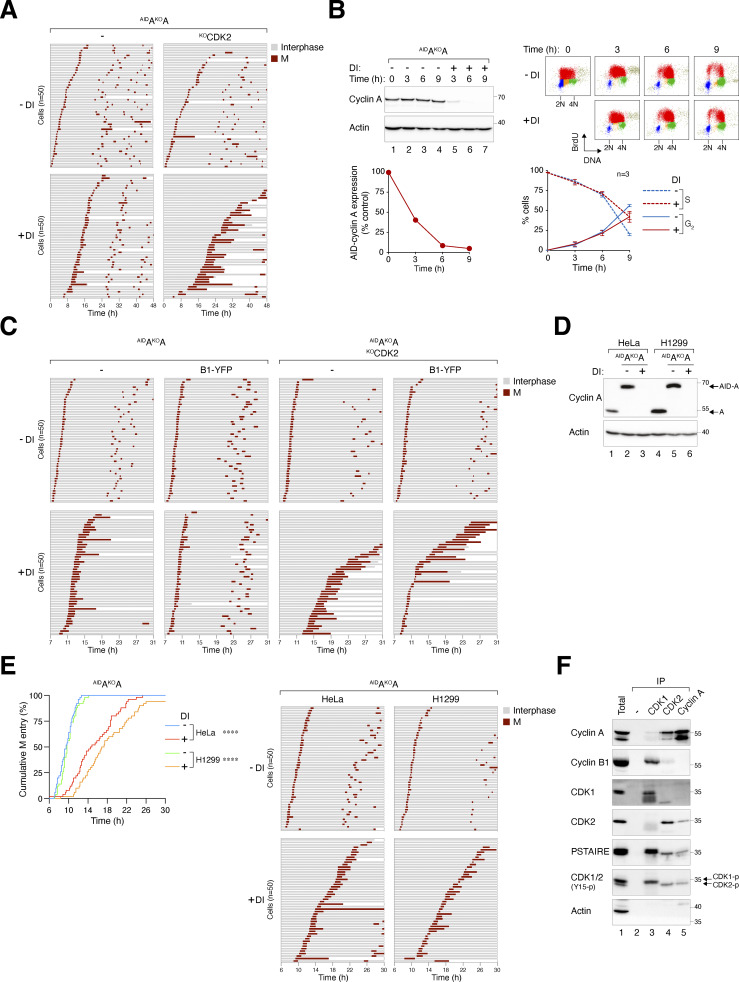
**Delayed mitotic entry in the absence of cyclin A and CDK2. (A)** Mitotic entry is significantly delayed in ^AID^A^KO^A and ^AID^A^KO^A^KO^CDK2 cells. Cells were pre-incubated with DI for 6 h to deplete cyclin A, followed by live-cell imaging for 48 h (*n* = 50). Key: interphase (grey); mitosis (red); and cell death (truncated bars). **(B)** Minimal delay in S phase progression upon cyclin A silencing. ^AID^A^KO^A cells were synchronized at S phase with double thymidine block and treated with DI at the second thymidine release to deplete ^AID^cyclin A. The cells were pulsed with BrdU for 30 min before harvested at each time points for immunoblotting analysis. The band intensity of ^AID^cyclin A was quantified and normalized to -DI control at *t* = 0 h. BrdU incorporation and DNA content were examined with bivariate flow cytometry (red: BrdU-positive; yellow: BrdU-negative S; blue: G_1_; green: G_2_/M). The percentages of S (BrdU-positive) and G_2_ cells were quantified (mean ± SEM from four independent experiments). **(C)** Alleviation of cyclin A^KO^-induced G_2_-M delay by cyclin B1. ^AID^A^KO^A and ^AID^A^KO^A^KO^CDK2 cells overexpressing cyclin B1 synchronized with double thymidine block were left untreated or treated with DI for 7 h to turn off cyclin A before time-lapse imaging. Time indicates the duration after thymidine release. Key: interphase (grey); mitosis (red); and cell death (truncated bars). **(D)** Conditional gene silencing of cyclin A in H1299 cells. HeLa and H1299 cells expressing ^AID^cyclin A without endogenous cyclin A were left untreated or treated with DI for 24 h before immunoblotting analysis. Lysates from HeLa and H1299 cells were included as controls for the relative expression of ^AID^cyclin A and endogenous cyclin A. **(E)** Cyclin A depletion induces G_2_-M delay in both HeLa and H1299 cells. ^AID^A^KO^A cells were synchronized using a double thymidine block and released into a drug-free or DI-containing medium. After 6 h, mitotic entry was analyzed using live-cell imaging (left panel; time indicates the duration after thymidine release). Raw data for individual cells are presented in the right panel. Key: interphase (grey); mitosis (red); and cell death (truncated bars). **(F)** Presence of cyclin B1–CDK2 complexes during normal G_2_. HeLa cells were synchronized at S phase with double thymidine block. After release into fresh medium for 5 h, NOC was added to prevent mitotic exit. After 4 h, mitotic cells were removed by washing, and the attached G_2_ cells were harvested for immunoprecipitation using antibodies against CDK1, CDK2, or cyclin A. Protein expression in the total lysates and immunoprecipitates (IP) was detected using immunoblotting. A negative control (no antibody was added) was included to assess the specificity of the immunoprecipitation. Source data are available for this figure: SourceData FS4.

To investigate G_2_–M without interference from cyclin A^KO^-induced replication stress, we synchronized cells using a double thymidine block before releasing them into a DI-containing medium. By 6 h after DI treatment, cyclin A was reduced to ∼5% compared with that during the S phase (Fig. S4 B). This approach ensured the presence of cyclin A throughout most of the S phase. Consistent with this, BrdU incorporation assays indicated that S phase completion was delayed in <20% of the cells upon cyclin A depletion (Fig. S4 B). Nevertheless, mitotic entry was significantly delayed, as indicated by a slower appearance of G_1_ cells (Fig. 7 G) and the delay in mean mitotic entry time (from 10 to 15 h after double thymidine release, Fig. 7 H). Disruption of CDK2 further enhanced the G_2_ delay caused by cyclin A deficiency, resulting in a delay of mean mitotic entry by 10 h. Essentially the same mitotic entry delay was obtained using ^AID^A^KO^A cell lines established from H1299 (Fig. S4, D and E), indicating that the delay in G_2_–M after cyclin A depletion is not specific to HeLa cells.

We further investigated whether additional cyclin B1 could compensate for the functions of cyclin A in mitotic entry. A YFP-tagged cyclin B1 was expressed in ^AID^A^KO^A and ^AID^A^KO^A^KO^CDK2 cells, resulting in roughly double the total cyclin B1 levels in these cells (Fig. 7 H). Notably, while ectopic cyclin B1 restored timely mitotic entry in cyclin A-deficient cells, it promoted mitotic entry relatively poorly in the absence of both cyclin A and CDK2, suggesting that cyclin B1 could promote mitosis by binding to CDK2 (Fig. 7 H).

While previous studies have reported that purified cyclin B1 can form non-canonical complexes with CDK2 in vitro (Brown et al., 2007; Desai et al., 1992; Pan and Hurwitz, 1993), cellular data are more contentious, as cyclin B1–CDK2 complexes were detected in normal cells but not in transformed cell lines including HeLa (Xiong et al., 1993). Our recent studies have indicated the formation of cyclin B1–CDK2 complexes in HeLa cells can be promoted by the depletion of CDK1 (Lau et al., 2021). We found that although CDK2 is primarily associated with cyclin A in G_2_ HeLa cells, a relatively small amount of cyclin B1 was detected in CDK2 immunoprecipitates (Fig. S4 F). Moreover, overexpressed cyclin B1 is associated with both CDK1 and CDK2 (Fig. 7 I). Furthermore, depletion of cyclin A resulted in an accumulation of cyclin B1–CDK2 complexes, while not affecting the abundance of cyclin B1–CDK1 complexes (Fig. 7 I). This suggests that the increased availability of CDK2 after cyclin A destruction enables the formation of more non-canonical cyclin B1–CDK2 complexes, particularly with overexpressed cyclin B1.

Collectively, these data indicate that G_2_–M is delayed in the absence of cyclin A and CDK2 independent of their S phase functions, and that cyclin B1 can overcome the effects of cyclin A deficiency in mitotic entry by forming non-canonical cyclin B1–CDK2 complexes.

### Plasticity between mitotic cyclins in different cell lines: A quantitative determination

It is intriguing that the critical roles of B-type cyclins, but not cyclin A, for mitosis in HeLa cells differ fundamentally from previous findings using RPE1 cells. Hégarat et al. observed that while depletion of cyclin B1 and B2 does not affect mitosis until after NEBD, the absence of cyclin A prevents mitotic entry in RPE1 cells (Hégarat et al., 2020). Given our findings that cyclin A and cyclin B are interchangeable in HeLa cells, provided sufficient levels are expressed, it is conceivable that the disparity between different studies may be reconciled by assessing the endogenous expression levels of mitotic cyclins in different cell lines.

**Figure S5.**
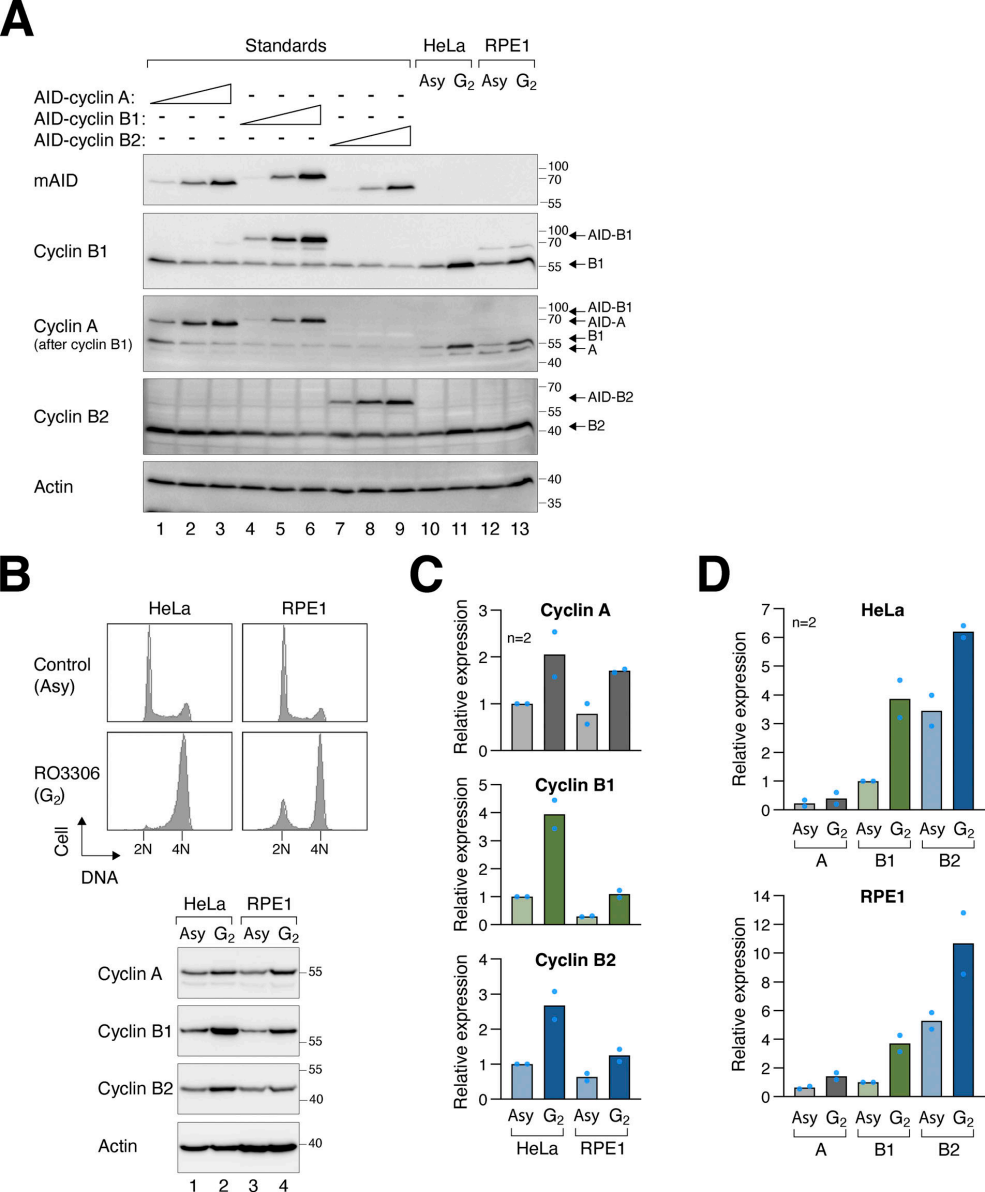
**Relative levels of intercellular and intracellular cyclins. (A)** A representative example of the immunoblotting analysis showing the relative levels of mitotic cyclins in HeLa and RPE1 cells. Different concentrations of plasmids expressing AID-cyclin A, B1, or B2 were transfected into HeLa cells to serve as standards. Lysates from asynchronous (Asy) and G_2_ HeLa and RPE1 cells were loaded. The samples were analyzed with antibodies against mAID or individual cyclins. **(B)** RPE1 cells contain relatively lower concentrations of B-type cyclins compared to HeLa cells. HeLa and RPE1 cells from asynchronous (Asy) and RO3306-treated (G_2_) populations were analyzed with flow cytometry or immunoblotting. **(C)** Comparison of the expression of mitotic cyclins in HeLa and RPE1 cells. The relative expression of cyclin A, B1, and B2 in asynchronous (Asy) and G_2_ lysates was determined using the respective AID-cyclin standards. The expression level of each cyclin was normalized to that cyclin in asynchronous HeLa cells. Mean of two independent experiments. **(D)** B-type cyclins are more abundant than cyclin A. The relative expression of cyclin A, B1, and B2 in asynchronous (Asy) and G_2_ lysates was determined using the respective AID-cyclin standards. The relative levels between each AID-cyclin were first determined using an antibody against AID. The expression level of endogenous cyclins was normalized to that of cyclin B1 in asynchronous HeLa (upper panel) or RPE1 (lower panel). Mean of two independent experiments.

To compare cyclin levels both within a cell line and between cell lines, we engineered recombinant cyclins with an identical N-terminal AID tag and loaded them side-by-side on immunoblots. The relative levels of different AID-cyclin standards were obtained using an antibody specific to mAID. Subsequently, the expression of endogenous cyclins in HeLa and RPE1 cells was assessed against the AID-cyclin standards using antibodies specific for each cyclin (Fig. S5 A). We analyzed mitotic cyclins in both asynchronous populations and in cells enriched in G_2_ using RO3306 (Fig. S5 B). These analyses revealed that while the expression of cyclin A was similar between HeLa and RPE1, both cyclin B1 and B2 were present at a higher level in HeLa than in RPE1 (Fig. S5 C). For example, at their peak levels in G_2_, cyclin B1 was fourfold higher in HeLa cells than in RPE1.

Using the AID-cyclin standards, we also determined the relative abundance of cyclin A, B1, and B2 within a particular cell line. We found that cyclin A was less abundant than cyclin B1, which in turn was less than cyclin B2 (Fig. S5 D). For example, in G_2_ HeLa cells, the level of cyclin B1 is 10-fold higher than cyclin A and 30% less than cyclin B2. These findings suggest that the apparent lack of function of cyclin B2 in mitotic entry was not due to its relatively low abundance, but likely due to a fundamental distinction between cyclin B2 and other mitotic cyclins. Moreover, the low abundance of cyclin A compared with B-type cyclins may explain the findings that endogenous cyclin A was insufficient in driving mitosis in the absence of cyclin B1 and B2 (Fig. 2). Increasing the expression of cyclin A, however, can overcome the lack of activities from B-type cyclins (Fig. 6).

Collectively, these results suggest that the relatively high abundance of B-type cyclins in HeLa cells compared with RPE1 may explain the higher reliance of HeLa cells on B-type cyclins for mitotic entry.

## Discussion

### Quantitative differences between A- and B-type cyclins as MPF

**Figure 8.**
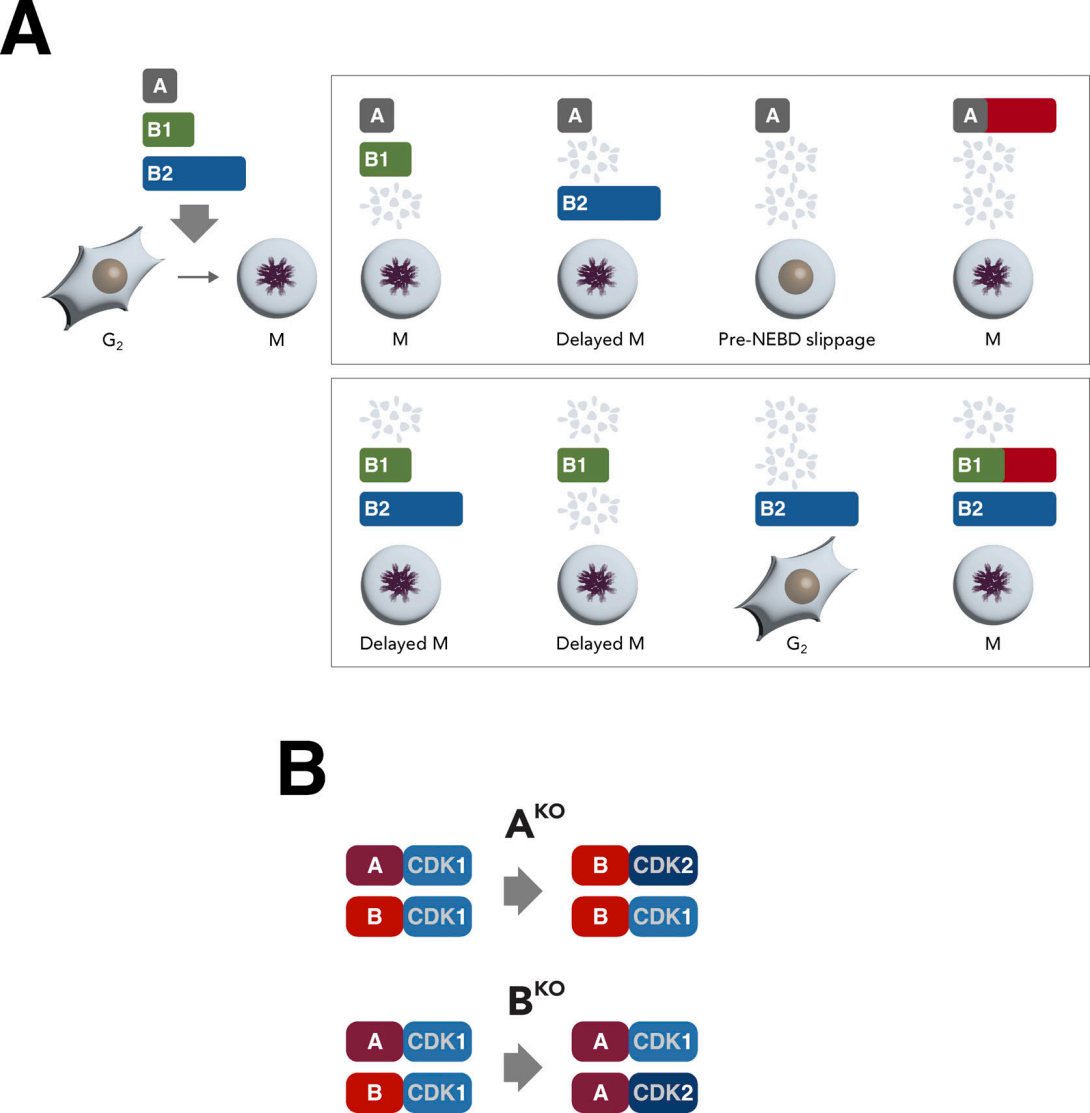
**Relative levels of intercellular and intracellular cyclins. (A)** Plasticity of mitotic cyclins in human cell lines. During G_2_, cyclin B2 is more abundant than cyclin B1 and cyclin A (represented in the diagram by the length of individual bars). The absence of cyclin B1 results in a delay but not inhibition of G_2_–M. By contrast, depletion of both cyclin B1 and B2 severely curtailed mitotic entry, leading to cell rounding followed by pre-NEBD slippage. The pre-NEBD G_2_ block can be rescued by overexpressing cyclin A (red bars). Similar to cyclin B1, cyclin A is rate-limiting for G_2_-M. The G_2_-M delay due to cyclin A deficiency can be rescued by overexpression of cyclin B1. Cells were blocked in G_2_ in the absence of cyclin A and cyclin B1. **(B)** Interplay between cyclin A and cyclin B. In the absence of cyclin A, G_2_–M is delayed with an accompanied accumulation of cyclin B1–CDK1 and cyclin B1–CDK2 complexes. This delay can be alleviated by overexpression of cyclin B1 (see A). Conversely, the absence of cyclin B1 and B2 hinders proper mitotic entry, leading to pre-NEBD slippage, accompanied by an enrichment of cyclin A-CDK1 and cyclin A-CDK2. Overexpression of cyclin A effectively overcome the pre-NEBD defects.

In this extensive examination of mitotic cyclins, we used a degron strategy for rapid cyclin removal in synchronized cell lines (see Fig. 8 A for a summary). In contrast with the effects of depleting cyclin B1 or cyclin B2 individually, the absence of both cyclin B1 and B2 prevented proper mitotic entry (Fig. 1 F and Fig. 2 A). However, cells deficient in cyclin B1 and B2 could initiate mitosis partially, as indicated by cell rounding (Fig. 2 D and Fig. 3), PLK1 activation (Fig. 4 A), and phosphorylation of specific CDK1 substrates (Fig. 4 B). Cell rounding was delayed for ∼2 h compared with cyclin B1-containing cells (Fig. 2 C). Moreover, mitotic progression did not proceed to NEBD (Fig. 3) or phosphorylation of histone H3^S10^ (Fig. 4 A). One mechanism that leads to cell rounding involves the disassembly of focal adhesions through cyclin B1–CDK1-dependent phosphorylation of kindlin (Chen et al., 2022). Hence, it is possible that phosphorylation of kindlin can still be carried out in the absence of cyclin B1 and B2.

It is noteworthy that, with a few exceptions, most proteins recognized by the pTPxK antibody were phosphorylated in both cyclin B-proficient and -deficient cells (Fig. 4 B). The pTPxK bands missing in cyclin B-silenced cells were unlikely to include ^mAID^cyclin B1 itself, as they were present in DI-treated ^mAID^B1^KO^B1 cells (Fig. S2 E) or ^mAID^B1^KO^B1B2 cells expressing cyclin A (Fig. 6 F). This suggests that cyclin A–CDK1 was sufficient in phosphorylating most cyclin B–CDK1 substrates, with the missing substrates likely specific to cyclin B–CDK1. Interestingly, the phosphorylation of these proteins remained elevated after pre-NEBD slippage (Fig. 4 B). Similar sustained phosphorylation was observed for the CDK1 substrate lamin A/C^S22^ and PLK1 substrate TCTP^S46^ (Fig. 4 A). An implication is that the phosphorylation of many CDK1 and PLK1 substrates may not be adequate and may not be directly linked to the mitotic state. For example, our results suggest that the phosphorylation of lamin A/C^S22^ is not sufficient to induce NEBD, as demonstrated by pre-NEBD slippage in ^mAID^B1^KO^B1B2 cells.

Loss of cyclin B is accompanied by an enrichment of both cyclin A–CDK1 and cyclin A–CDK2 complexes (see Fig. 8 B for a summary). Despite the upsurge of cyclin A–CDK1/2 complexes, endogenous cyclin A can only partially compensate for cyclin B’s functions (Fig. 5 A; and Fig. 6, G and H). It is plausible that CDK inhibitors such as p21 and p27 could suppress cyclin A–CDK1/2 activity under these conditions. However, we do not favor the hypothesis, as we found that deletion of p21 or p27 with siRNAs does not promote mitotic entry in the absence of cyclin B (our unpublished data).

Surprisingly, a several-fold increase in cyclin A fully compensated for cyclin B’s functions, restoring timely mitotic onset (Fig. 6 E), proper mitotic progression (Fig. 6 D), and largely rescued long-term survival (Fig. 6 C) in the absence of B-type cyclins. The normal phosphorylation pattern of histone H3^S10^ and pTPxK CDK1 substrates was also re-established (Fig. 6 F). As expected, overexpression of cyclin A resulted in an increase in cyclin A–CDK1/2 complexes (Fig. 6, G and H). The effects of cyclin A were dose-dependent, as reducing the amount of exogenous cyclin A failed to fully restore mitosis (Fig. 6). Notably, the CDK1 substrates that were unphosphorylated in cyclin B-deficient cells became phosphorylated in cells overexpressing cyclin A, indicating that their absence was not due to fundamental differences between cyclin A and cyclin B (Fig. 6 F). Overall, these results demonstrate that elevated cyclin A levels can effectively substitute for the absence of B-type cyclins in facilitating mitotic entry. It is noteworthy that previous studies have demonstrated that ectopically expressed cyclin A can overcome the cell cycle block associated with cyclin A and cyclin B double mutants in *Drosophila* embryos (Knoblich and Lehner, 1993).

The pre-NEBD mitosis in the absence of B-type cyclins was prematurely terminated by a process we termed pre-NEBD slippage (Fig. 2 D and Fig. S3 F). Given the absence of DNA re-replication observed with flow cytometry (Fig. 1 E), it is plausible that cyclin B-deficient cells did not progress into the S phase following pre-NEBD slippage. In normal cells, mitotic slippage following mitotic arrest is induced by gradual degradation of cyclin B (Brito and Rieder, 2006) and activation of APC/C (Lok et al., 2020). By contrast, APC/C was not activated in cyclin B-silenced cells, as indicated by data from an APC/C reporter (Fig. S2 C) and stabilized APC/C substrates (Fig. 4 A and Fig. 6 F). However, as there was a transient reduction of cyclin A (Fig. 4 A), we cannot rule out the possibility that there was a partial activation of APC/C. Given that APC/C activation relies on cyclin B-dependent phosphorylation (Yamano, 2019), it is possible that endogenous cyclin A could drive early mitotic events such as cell rounding but not full APC/C phosphorylation. However, APC/C was activated normally in cyclin B-deficient cells overexpressing cyclin A, suggesting that the effect may not be due to a fundamental difference between cyclin A and cyclin B (Fig. 6 F).

At first glance, it seems unusual for cyclin B-deficient cells to exit mitosis without APC/C-dependent degradation of its targets such as cyclin A (Fig. 4 A). A reasonable surmise is that APC/C-dependent degradation may not be necessary for pre-NEBD slippage. If endogenous cyclin A indeed plays a role in initiating partial mitosis in the absence of cyclin B, an interesting question arises regarding how cyclin A–CDK is inactivated during pre-NEBD slippage. EMI1, an APC/C inhibitor, is unlikely to be responsible, as its expression was similarly regulated in the presence or absence of cyclin B (Fig. 4 A). Given the transient reduction of cyclin A around the time of pre-NEBD slippage (Fig. 4 A), one possibility is that a partial decrease of cyclin A is sufficient to impede its ability to sustain mitosis without cyclin B. Another potential reason for mitotic exit in the presence of sustained cyclin A could involve the Greatwall kinase pathway (Castro and Lorca, 2018). Modulating the inhibition of PP2A-B55, which counters CDK1-dependent phosphorylation, could potentially facilitate mitotic exit.

The absence of NEBD in cyclin B-deficient cells contrasts with previous RNAi studies that showed no impact on NEBD when cyclin B1 and B2 were knocked down in HeLa cells (Chen et al., 2008; Gong et al., 2007). The different results obtained from RNAi studies could simply be due to the more rapid and thorough cyclin B depletion achieved through CRISPR- and degron-based approaches. However, Hégarat et al., using a degron strategy, observed that unlike in HeLa cells, depletion of cyclin B1 and B2 did not affect mitotic entry in RPE1 cells. Only a minor fraction of cyclin B-depleted RPE1 cells (<10%) underwent pre-NEBD slippage-like behavior (Hégarat et al., 2020). We believe a potential explanation is that cyclin B1 and B2 were present at a lower level in RPE1 in comparison to HeLa (Fig. S5 C). The comparable levels of cyclin A in HeLa and RPE1, along with cyclin A’s ability to substitute for B-type cyclins functions, suggest a potentially lower reliance on B-type cyclins for mitotic entry in RPE1 cells.

Our findings suggest that in mitosis, cyclin A and cyclin B exhibit functional equivalence, with their differences being primarily quantitative rather than fundamental (Fig. 8 A). This idea parallels our previous observations on the roles of CDK1 and CDK2 in human cells, where overexpression of one CDK can effectively replace the functions of other CDKs throughout the cell cycle (Lau et al., 2021). It is conceivable that cyclin and CDK pathways are highly adaptable and easily reconfigured. Exploring whether the rewiring of mitotic cyclin levels is a common feature in cancer cells by examining additional normal and cancer cell lines could yield valuable insights.

### Comprehensive compensation of cyclin B2 function by other mitotic cyclins

Disruption of cyclin B2 had minimal effects on proliferation in HeLa cells (Fig. 1, E and F; and Fig. 2, A and B). The dispensable nature of cyclin B2 may explain our observation of the decline in exogenous ^AID^cyclin B2 expression during prolonged culturing (Fig. 1 D). The lack of specific functions attributable to cyclin B2 from knockout studies is interesting, given that cyclin B1 and B2 exhibit distinct localization patterns during G_2_–M (Bentley et al., 2007; Bentley et al., 2007; Chen et al., 2008; Hagting et al., 1998; Jackman et al., 1995; Pines and Hunter, 1991; Pines and Hunter, 1994; Toyoshima et al., 1998). Nonetheless, cyclin B2’s mitotic-promoting functions can clearly be seen in the cyclin B1-deficient background. While depletion of cyclin B1 alone delayed mitotic entry, depletion of cyclin B1 and B2 together prevented mitotic progression beyond cell rounding (Fig. 2).

An interesting question arises regarding cyclin B2’s role in promoting mitosis without cyclin B1. While this scenario is not observed in mice (Brandeis et al., 1998), findings in human cell lines have been more contentious (see Introduction). In our study, the depletion of cyclin B1 resulted in a slight but significant extension of interphase (Fig. 2 C and Fig. S2 D) and mitosis (Fig. 1 A), along with compromised long-term survival (Fig. 1 F). These observations suggest that cyclin B2 may partially compensate for cyclin B1’s functions. Nonetheless, this interpretation is likely too simplistic a view considering the additional compensation provided by cyclin A.

If we hypothesize that cyclin B1 and B2 are functionally equivalent, then the distinct results obtained from their depletion imply that cyclin B1 is either more abundant and/or more active than cyclin B2. However, our analyses indicate that cyclin B2 is in fact more abundant than cyclin B1 in cells (Fig. S5 D), suggesting that cyclin B2 cannot fully substitute for cyclin B1’s functions (see Fig. 8 A for a summary).

### Cyclin A contributes to but is not essential for mitotic entry in cancer cell lines

Using a similar degron strategy to rapidly deplete cyclin A in HeLa and H1299 cells, our overall findings demonstrate that cyclin A plays a rate-limiting but not indispensable role in G_2_-M. Silencing of cyclin A in asynchronously growing cells was cytotoxic, as indicated by the increase in S and G_2_/M population (Fig. 7, B and C), prolonged cell cycle duration (Fig. 7 D), increased apoptosis (Fig. 7 A), and reduced clonogenic survival (Fig. 7 F). Targeting cyclin A specifically during G_2_ revealed that it has distinct rate-limiting functions in G_2_-M, independent of its role in DNA replication, causing a delay in mitotic entry by ∼5 h (Fig. 7, G and H).

The G_2_–M delay resulting from cyclin A depletion doubled with concurrent deletion of CDK2. CDK2 itself is a non-essential gene in HeLa due to compensation by CDK1 (Lau et al., 2021), and its disruption did not influence the G_2_–M timing (Fig. 7 H). The extensive G_2_–M delay observed in the absence of both cyclin A and CDK2 suggests that cyclin B1–CDK1 was less capable of compensating for cyclin A–CDK2 than cyclin B1–CDK2, which normally is present as a minor species (Fig. S4 F). The involvement of non-canonical cyclin B1–CDK2 is further revealed by the restoration of timely mitotic entry by cyclin B1 in a cyclin A-deficient background, which was less effective in cells lacking both cyclin A and CDK2 (Fig. 7 H). In HeLa cells, a twofold increase in cyclin B1 was sufficient to restore timely mitotic entry in the absence of cyclin A (Fig. 7 H), highlighting the plasticity between the A-and B-type cyclins in G_2_–M. Moreover, the levels of cyclin B1–CDK2 increased upon cyclin A depletion (Fig. 7 I), likely because overexpressed cyclin B1 was saturating CDK1. In RPE1 cells, S and G_2_ defects from cyclin A depletion can also be partially compensated by cyclin B1 overexpression (Hégarat et al., 2020; Moore et al., 2003). Notably, nuclear-targeting cyclin B1 was more effective than normal cyclin B1 in rescuing cyclin A’s functions.

Similar to B-type cyclins, the significance of cyclin A in mitosis appears to vary between different cell types. While cyclin A is dispensable for mitosis in HeLa (this study) and mouse fibroblasts (Kalaszczynska et al., 2009), it plays a vital role in G_2_–M progression in RPE1 cells (Hégarat et al., 2020). The disparities across cell types may stem from intra- and intercell line variations in mitotic cyclin expression (Fig. S5, C and D). It is possible that the relatively high levels of cyclin A and low levels of B-type cyclins in normal cells (RPE1) may give greater weight to cyclin A compared with cancer cell lines like HeLa. As cyclin A is frequently overexpressed in cancer cells (reviewed in Yam et al. [2002]), it is possible that the overexpressed cyclin A can promote unscheduled entry into mitosis even in cells with normal regulation of cyclin B–CDK1. Collectively, the ability of previously thought distinct mitotic cyclins to replace each other highlights their plasticity and quantitative differences.

## Materials and methods

### Plasmids

CRISPR-Cas9 plasmids were generated by annealing the indicated pairs of oligonucleotides followed by ligation into BbsI-cut pX330 (a gift from Feng Zhang; obtained from Addgene; #42230; Addgene): cyclin A (5′-CAC​CGC​AGT​ATG​AGA​GCT​ATC​CTC​G-3′ and 5′-AAA​CCG​AGG​ATA​GCT​CTC​ATA​CTG​C-3′); cyclin B1 (5′-CAC​CGC​CTA​ATT​GAC​TGG​CTA​GTA​C-3′ and 5′-AAA​CGT​ACT​AGC​CAG​TCA​ATT​AGG​C-3′); and cyclin B2 (5′-CAC​CGA​GAC​TCT​GTA​CAT​GTG​CGT-3′ and 5′-AAA​CAC​GCA​CAT​GTA​CAG​AGT​CTC-3′). CDK2 CRISPR-Cas9 in pX330 was generated as described previously (Ng et al., 2019).

The vector pUHD-SB-C-mAID/Hyg was generated by inserting a PCR product (template: pUHD-SB-mAID/Hyg [Yeung et al., 2021]; primers: 5′-GAG​CTC​GGT​ACC​CGG​GGA​TCC​AAG​GAG​AAA-3′ and 5′-TCT​ATC​GAT​CTT​ATC​ATG​TCT​ACT​TAT​ACA​TCC​TC-3′) into BamHI-cut pUHD-SB/Hyg (Yeung et al., 2021) using the SLiCE cloning method (Motohashi, 2015). FLAG-cyclin B1 in pUHD-P3 was generated by ligating NcoI-BamHI-cut FLAG-cyclin B1 in pUHD-P1/Pur (Yeung et al., 2023) into NcoI-BamHI-cut pUHD-P3 (Ma et al., 2009) (previously called pUHD-P1-3C). Cyclin B1-mAID in pUHD-SB-C-mAID/Hyg (containing CRISPR-resistant silent mutations) was generated by inserting a PCR product obtained using a double PCR method (template: FLAG-cyclin B1 in pUHD-P3; primers: 5′-TCT​GTT​TCA​GGG​GCC​CAT​GGC​GCT​CCG-3′ and 5′-TTT​GAA​CCT​GAA​CCA​ACC​AGT​CAA​T-3′; 5′-ATT​GAC​TGG​TTG​GTT​CAG​GTT​CAA​A-3′ and 5′-CGG​GTA​CCG​AGC​TCG​AAT​TCC​ACC​TTT​GCC​ACA-3′) into NcoI-EcoRI-cut pUHD-SB-C-mAID/Hyg using SLiCE cloning. AID-cyclin B1 in pRevTRE-AID/Hyg was generated by inserting a PCR product obtained using a double PCR method (template: FLAG-cyclin B1 in pUHD-P3; primers: 5′-CTG​GTT​GGT​CCA​AGT​TCA​AAT​G-3′ and 5′-TAT​CTT​ATC​ATG​TCT​GGA​TCC-3′; 5′-AGC​TCG​TTT​AGT​GAA​CCG​TCA​GAT​CG-3′ and 5′-CTT​GGA​CCA​ACC​AGT​CAA​TTA​GG-3′) cut with NcoI-BamHI into NcoI-BamHI-cut pRevTRE-AID/Hyg (Ng et al., 2023).

Cyclin B1-YFP-SNAP in pLNCX2 (Schnerch et al., 2013) was a gift from Ralph Wäsch (University Medical Center Freiburg, Freiburg, Germany). Cyclin B1 was removed from the construct by inserting the PCR product (template: cyclin B1-YFP-SNAP in pLNCX2; primers: 5′-TAC​CGG​ACT​CAG​ATC​TCA​TGG​TGA​GCA​AG-3′ and 5′-CAT​TAA​GGC​CTG​TCG​ACA​AG-3′) into BgIII-NotI-cut cyclin B1-YFP-SNAP in pLNCX2 using SLiCE cloning to generate YFP-SNAP in pLNCX2.

The cDNA of cyclin B2 was obtained from Geneservice (IMAGE clone#8144089) and amplified with 5′-AAC​CAT​GGC​GCT​GCT​CCG​ACG​CC-3′ and 5′-AGG​ATC​CCT​AGG​ACC​TTC​CTA​TCA​GT-3′. The PCR product was digested with NcoI and BamHI and ligated into NcoI-BamHI-cut pUHD-P1 (Yam et al., 1999) to generate FLAG-cyclin B2 in pUHD-P1. CRISPR-resistant silent mutations were introduced into cyclin B2 with a double PCR method using primers: 5′-AGC​TCG​TTT​AGT​GAA​CCG​TCA​GAT​CG-3′ and 5′-CAC​ACA​TAT​ATA​ACG​TTT​CCT​GCA​GAA​G-3′; 5′-AAC​GTT​ATA​TAT​GTG​TGT​TGG​CAT​TAT​GG-3′ and 5′-CCG​ATC​AAT​AGA​TCT​TAT​CAT​GTC​TG-3′. The PCR product was digested with NcoI and BamHI and ligated into NcoI-BamHI-cut pUHD-SB-AID/Hyg (Yeung et al., 2021) to generate AID-cyclin B2 in pUHD-SB-AID/Hyg.

Cyclin A-mRFP in pLNCX2 was generated by inserting a PCR product (template: FLAG-cyclin A in pUHD-P1 [Yam et al., 2000]; a double PCR method using primers: 5′-AGC​TCG​TTT​AGT​GAA​CCG​TCA​GAT​CG-3′ and 5′-TCG​ATG​TTA​GGC​CAT​TAT​CAT​GTC​TGG​A-3′; 5′-CCA​CTC​CAC​CGG​CGC​CTC​TAA​AGC​CAT-3′ and 5′-TCG​ATG​TTA​GGC​CAT​TAT​CAT​GTC​TGG​A-3′) into SaII-cut mRFP in pLNCX2 (Lau et al., 2021) using SLiCE cloning. AID-cyclin A in pUHD-SB-AID/Hyg was described previously (Ng et al., 2023).

CDK1-mRFP was generated by inserting a PCR product (template: AID-CDK1 in pUHD-SB-AID/Hyg (Yeung et al., 2021); primers: 5′-TCC​TTC​TCT​AGG​CGC​CGG​CCA​TGG​AAG​ATT​AT-3′ and 5′-GAT​CTC​CTG​ATC​CTC​CTG​ATC​CCA​TCT​TCT​TAA​T-3′) into BamHI-EcoRI-cut IMS-mRFP (a gift from Jade Shi, Hong Kong Baptist University) using SLiCE cloning. CDK1-mRFP in pLNCX2 was generated by inserting a PCR product (template: CDK1-mRFP in IMS-mRFP; primers: 5′-TAA​GGC​CTG​TCG​ACA​AGC​GGC​GAA​TTC​TTA​G-3′ and 5′-TAG​CGC​TAC​CGG​ACT​CAG​ATC​CTT​CTC​TAG​GC-3′) into BglII-NotI-cut cyclin B1-YFP-SNAP in pLNCX2 using SLiCE cloning. CDK2-mRFP in pLNCX2 and mRFP in pLNCX2 were described previously (Lau et al., 2021).

APC/C reporter (mRFP-cyclin B1[CΔ62] in pSBbi-TIR1/Bla) was described previously (Lau et al., 2021). pCMV(CAT)T7-SB100 expressing Sleeping Beauty transposase was a gift from Zsuzsanna Izsvak (#34879; Addgene).

RFP-NLS-myc in pSB-TIR1/Bsd was generated using a double PCR method (first PCR template: pUHD-P3T [Ma et al., 2009]; primers: 5′-TCC​CAT​GGG​AGT​CGA​CGG​ATC​CGA​GGA​CG-3′ and 5′-TTC​TCG​AGG​CCG​GTG​GAG​T-3′; second PCR template: pSB-LacI-VP48-NLS-3xmyc-IKZF3/Bla (Yeung et al., 2023); primers: 5′-CAC​CGG​CCT​CGA​GAA​CAA​ACG​GG-3′ and 5′-CCA​AGC​TTT​TTA​GCT​AGT​GGA​TCC​G-3′). The PCR product was digested with NcoI and HindIII and ligated into NcoI-HindIII-cut pSBbi-TIR1/Bla (Lau et al., 2021).

mRFP-lamin A in pIRESpuro3 was generated by ligating the XhoI-BamHI fragment of GFP-lamin A in pEGFP (a gift from Zhongjun Zhou, the University of Hong Kong) and the NheI-XhoI fragment of mRFP1 in pUHD-P3T (Ma et al., 2009) with NheI-BamHI-cut pIRESpuro3.

EGFP-BAF was a gift from Daniel Gerlich (#101772; Addgene). Histone H2B-GFP in pEF/Bsd was a gift from Tim Hunt (Cancer Research UK). Histone H2B-mRFP in pRevTRE2 was described previously (Marxer et al., 2012).

### siRNA

Stealth siRNA targeting cyclin A (5′-GCU​AUG​CUG​UUA​GCC​UCA​AAG​UUU​G-3′) and control siRNA were manufactured by Thermo Fisher Scientific. Transfection of siRNA (10 nM) was carried out using Lipofectamine RNAiMAX (Thermo Fisher Scientific) according to the manufacturer’s instructions.

### Cell lines

HeLa used in this study was a clone that expresses the tTA tetracycline transactivator (Yam et al., 2000). H1299 and RPE1 were obtained from the American Type Culture Collection.

Conditional cyclin B cells were generated by transfecting HeLa cells with cyclin B1-mAID in pUHD-SB-C-mAID/Hyg (for ^mAID^B1^KO^B1 and ^mAID^B1^KO^B1B2), cyclin B1 CRISPR-Cas9 in pX330 (for ^mAID^B1^KO^B1 and ^mAID^B1^KO^B1B2), AID-cyclin B2 in pUHD-SB-AID/Hyg (for ^AID^B2^KO^B2), cyclin B2 CRISPR-Cas9 in pX330 (for ^AID^B2^KO^B2 and ^mAID^B1^KO^B1B2), pSBbi-TIR1/Pur, and transposase (pCMV(CAT)T7-SB100), followed by selection with hygromycin and puromycin for ∼2 wk. Cyclin B2-disrupted cells were generated by transfecting HeLa cells with cyclin B2 CRISPR-Cas9 in pX330 and a blasticidin-expressing construct. The transfected cells were enriched by selection with blasticidin for 48 h before single colony isolation. For cells expressing mRFP, CDK1-mRFP, and CDK2-mRFP, ^mAID^B1^KO^B1B2 cells were transfected with mRFP in pLNCX2, CDK1-mRFP in pLNCX2, and CDK2-mRFP in pLNCX2, respectively. A mixed population of mRFP-positive cells was sorted using flow cytometry. For cells expressing cyclin A, ^mAID^B1^KO^B1B2 cells were transfected with cyclin A-mRFP in pLNCX2 and sorted with flow cytometry based on gating the upper 25% (high levels of overexpression) and lower 25% (low levels of overexpression) of mRFP.

Cell lines ^AID^A^KO^A and ^AID^A^KO^A^KO^CDK2 were generated as previously described (Ng et al., 2023). ^AID^A^KO^A cells from H1299 were established by transfecting H1299 cells with AID-cyclin A in pUHD-SB-AID/Hyg, pSBbi-TIR1-tTA/Pur (Yeung et al., 2021), cyclin A CRISPR-Cas9, and Sleeping Beauty transposase (pCMV(CAT)T7-SB100) followed by selection with hygromycin and puromycin for 2 wk.

Single-cell–derived colonies were obtained by limiting dilution in 96-well plates. Cells stably expressing histone H2B–GFP were generated by infecting the cells with retroviruses created by cotransfecting histone H2B–GFP–expressing retrovirus construct (a gift from George Tsao, the University of Hong Kong), VSV-G, and pCL-Ampl plasmids into Phoenix-gp cells (Pear et al., 1993) in the presence of 5 µg/ml of polybrene (Sigma-Aldrich). The infected cells were enriched by sorting GFP-positive cells using flow cytometry (FACSAria III; BD Biosciences). ^AID^A^KO^A cells expressing cyclin B1-YFP were generated similarly by using a cyclin B1-YFP retrovirus construct (a gift from Ralph Wäsch, University Medical Center Freiburg, Freiburg, Germany).

### Cell culture

Cells were propagated in Dulbecco’s modified Eagle’s medium (DMEM) supplemented with 10% (vol/vol) calf serum (for HeLa) or fetal bovine serum (for H1299) and 50 U/ml of penicillin–streptomycin (Thermo Fisher Scientific). Unless specified, cells were treated with the following reagents at the indicated final concentrations: AZ3146 (Selleck Chemicals; 2 µM), blasticidin (2.5 µg/ml; Thermo Fisher Scientific), doxycycline (Dox) (2 µg/ml; Sigma-Aldrich), hygromycin B (0.25 mg/ml; Thermo Fisher Scientific), IAA (50 µg/ml; Sigma-Aldrich), NOC (100 ng/ml; Sigma-Aldrich), puromycin (0.3 µg/ml; Sigma-Aldrich), RO3306 (10 µM for HeLa cells and 5 µM for RPE1 cells; Santa Cruz Biotechnology), and thymidine (2 mM; Santa Cruz Biotechnology). Transfection was performed using a calcium phosphate precipitation method (Kingston et al., 2003). Cell-free extracts were prepared as described previously (Yeung et al., 2023).

### Synchronization

Synchronization using double thymidine was conducted following established protocols (Ma and Poon, 2017). Briefly, cells were grown in a medium containing 2 mM of thymidine for 14 h. The cells were then washed twice with PBS and cultured in a fresh medium. After 9 h, the cells were incubated with a second round of 2 mM of thymidine for 14 h. G_2_ cells were harvested at 9 h after release from the double thymidine block. Enrichment of G_2_-arrested cells was achieved by exposing cells to a medium containing RO3306 for 16 h.

### Clonogenic survival assay

Cells were seeded at a density of 300 cells per 60-mm plate and treated with DMSO or DI. After 2 wk, the colonies were fixed with methanol:acetic acid (2:1) and stained with 2% crystal violet. The number of colonies was quantified by eye and normalized to the mock-treated group.

### Sequencing and indel analysis

Purified genomic DNA from ^mAID^B1^KO^B1B2 cells were amplified with PCR using primers 5′-AAT​GCT​GCA​GAC​ATC​ACA​GCC-3′ and 5′-CTT​GGT​CAG​CAG​CAG​TGT​TCC-3′; 5′-AGC​AGG​AGA​TGG​GTC​AGC​GA-3′ and 5′-GCT​CCT​AGT​CAC​CCT​TCC​CAC-3′, which covers cyclin B1 and B2 CRISPR–Cas9 edited locus, respectively. The cyclin B1 and B2 PCR products were sequenced using 5′-CCT​AAT​TGA​CTG​GCT​AGT​AC-3′ and 5′-GAG​ACT​CTG​TAC​ATG​TGC​GT-3′, respectively. Indel analysis was conducted using the ICE Analysis Tool (Synthego Performance Analysis, ICE Analysis. 2019. v2.0).

### Flow cytometry

Flow cytometry analysis after propidium iodide staining was performed as described previously (Mak et al., 2020). Briefly, cells were trypsinized, washed with PBS, and fixed with ice-cold 80% ethanol. The cells were then stained with a solution containing 40 µg/ml of propidium iodide and 40 µg/ml of RNaseA at 37°C for 30 min. The DNA content of 10,000 cells was analyzed using FACSAria III flow cytometer (BD Biosciences).

For BrdU incorporation analysis, cells were pulsed with 10 µM of BrdU (Sigma-Aldrich) for 30 min before harvesting. The cells were then fixed with ice-cold 80% ethanol. After centrifugation at 930 *g* for 5 min, the pellet was washed twice with PBS, followed by incubation with freshly made 2 M HCl at 25°C for 20 min with gentle mixing. To neutralize the HCl, the cells were incubated with 0.1 M sodium borate buffer (pH 8.5) at 25°C for 5 min. The cells were collected by centrifugation after each wash step, being washed twice with PBS and once with PBST (PBS with 0.5% Tween 20 and 0.05% wt/vol BSA). The cell pellet was resuspended in residue buffer and incubated with 2 μl of anti-BrdU antibody (sc-20045; Santa Cruz Biotechnology) at 25°C for 1.5 h. The cells were then washed twice with PBST before incubating with 2 μl of Alexa Fluor-488 goat anti-mouse IgG antibody (Thermo Fisher Scientific) at 25°C for 1 h in the dark. After two washes with PBST, the cells were stained with propidium iodide and subjected to flow cytometry analysis.

### Antibodies and immunological methods

The following antibodies were obtained from the indicated sources: β-actin (Cat# A5316; RRID:AB_476743; Sigma-Aldrich), phospho-AURKA^T288^/AURKB^T232^/AURKC^T198^ (2914; Cell Signaling Technology; Cat# 2914; RRID:AB_2061631), CDK1 (Cat# sc-54; RRID:AB_627224; Santa Cruz Biotechnology), CDK1 (for immunoprecipitation; a custom-made polyclonal antibody raised against the peptide CHPYFNDLDNQIKKM; Genscript), CDK2 (Cat# sc-6248; RRID:AB_627238; Santa Cruz Biotechnology), phospho-CDK1/2^Y15^ (Cat# 612307; RRID:AB_399622; BD Biosciences or Cat# ab76146; RRID:AB_1310069; Abcam), cyclin A2 (AT10; a gift from Tim Hunt, Cancer Research UK); cyclin B1 (Cat# sc-245; RRID:AB_627338; Santa Cruz Biotechnology or V152; a gift from Julian Gannon, Cancer Research UK), cyclin B2 (Cat# sc-22776; RRID:AB_2072392; Santa Cruz Biotechnology), cyclin E1 (Cat# sc-247; RRID:AB_627357; Santa Cruz Biotechnology), mAID (Cat# M214-3; MBL International; RRID:AB_2890014), phospho-CDK substrates (TPXK motif) (Cat# 14371; Cell Signaling Technology; RRID:AB_2798466), EMI1 (Cat# 37-6600; Zymed Laboratories; RRID:AB_2533333), phosphoryated histone H3^S10^ (Cat# sc-8656R; Santa Cruz Biotechnology; RRID:AB_653256), phospho-lamin A/C^S22^ (S22-p) (Cat# 2026; Cell Signaling Technology; RRID:AB_2136155), cleaved PARP1 (Cat# 552597; BD Biosciences; RRID:AB_394438), PLK1 (Cat# sc-17783; Santa Cruz Biotechnology; RRID:AB_628157), phospho-PLK1^T210^ (Cat# 9062; Cell Signaling Technology; RRID:AB_11127447), PSTAIRE (a gift from Masakane Yamashita, Hokkaido University, Sappoo, Japan), PTTG1 (Cat# sc-56207; Santa Cruz Biotechnology; RRID:AB_785382), TCTP (Cat# 5128; Santa Cruz Biotechnology; RRID:AB_11220419), and phospho-TCTP^S46^ (Cat# 5251; Cell Signaling Technology; RRID:AB_10547143).

Immunoblotting was performed as described previously (Ng et al., 2019). The positions of molecular size standards (in kDa) are indicated in the Figures. Quantification of signals on immunoblotting was conducted using Image Lab software (version 5.2.1 build 11; Bio-Rad Laboratories). Band intensities were quantified using standard curves generated from serially diluted lysates and normalized with actin signals.

Immunoprecipitation was performed by incubating 500 µg of cell-free extracts with 1 µg of antiserum or 1.5 µg of purified antibodies at 4°C for 1 h. The antibody–antigen complex was precipitated by diluting the reaction with 400 μl of bead buffer (50 mM Tris-Cl pH 7.4, 5 mM NaF, 250 mM NaCl, 5 mM EDTA, 5 mM EGTA, 0.1% Nonidet P-40) and 10 μl of protein A/G PLUS-Agarose (Santa Cruz Biotechnology). After incubation at 4°C for 1 h with end-over-end rotation, the beads were washed three times with bead buffer (1 ml each). The beads were then transferred to a new tube and washed three times. The beads were then mixed with 55 μl of 6× sample buffer and boiled for 5 min before being used for immunoblotting analysis.

### Immunostaining

Samples for immunofluorescence analysis were prepared as described previously (Lau et al., 2021). Briefly, cells were cultured on 0.1% poly-L-lysine-coated coverslips and fixed with ice-cold methanol at −20°C for 10 min. Permeabilization was carried out with 0.4% Triton X-100 in PBS at room temperature for 30 min. Primary antibodies against lamin A were applied at room temperature for 1 h, followed by incubation with secondary antibody Alexa-Fluor-647 goat anti-rabbit IgG at room temperature for 1 h. For immunolabeling of microtubules, samples were incubated with Alexa-Fluor-488-conjugated alpha-tubulin antibodies overnight at 4°C. Nuclei were counterstained using 200 ng/ml of Hoechst 33258 at room temperature for 10 min. Samples were washed with 0.1% Triton X-100 in PBS three times for 5 min each between each labeling step. After the final wash, cells were mounted onto coverslips using 2% N-propyl gallate (Sigma-Aldrich) in glycerol. Data acquisition was carried out using an LSM 980 confocal microscope with AiryScan 2 for super-resolution imaging. Z-stack images were acquired using a 63×/1.4 oil-immersion objective using an Airyscan detector to cover 5-µm thickness with a step size of 0.5 µm. Representative images shown are maximal projections of captured Z-stack images.

### Live-cell imaging

Cells were seeded onto 24-well cell culture plates and placed into an automated microscopy system equipped with a temperature, humidity, and CO_2_ control chamber (Zeiss Celldiscoverer 7). Images were captured every 5 or 10 min. Data acquisition was carried out using Zeiss ZEN 2.3 (blue edition), and subsequent analysis was performed using ImageJ (National Institutes of Health, Bethesda, MD, USA). Mitosis was defined as from cell rounding and/or DNA condensation to anaphase onset or mitotic slippage. Following mitosis, one daughter cell was randomly selected and tracked. Apoptosis was determined based on morphological changes.

Live-cell imaging using a confocal microscope was performed using LSM 980 confocal microscope with AiryScan 2 for super-resolution imaging. Z-stack images were captured using 63×/1.4 oil-immersion objective and Airyscan detector, covering 19-µm thickness with a step size of 1.2 µm.

### Statistical analysis

Statistical significance was determined using the Mann–Whitney test (two-tailed). SuperPlots (Lord et al., 2020) were generated using Prism (version 10.1.1(270); GraphPad Software). Box-and-whisker plots were created, with center lines showing the medians; box limits indicating the interquartile range; and whiskers extending to the most extreme data points that were no >1.5 times the interquartile range from the 25th and 75th percentiles.

### Online supplemental material

Fig. S1 shows gene silencing of cyclin B1 and cyclin B2. Fig. S2 shows that conditional depletion of cyclin B induces defective mitotic entry and mitotic slippage. Fig. S3 shows rescue of cell cycle defects caused by cyclin B deficiency with cyclin B1 and cyclin A. Fig. S4 shows delayed mitotic entry in the absence of cyclin A and CDK2. Fig. S5 shows relative levels of intercellular and intracellular cyclins. Video 1 shows normal mitosis in control cells. Video 2 shows pre-NEBD slippage in cyclin B-deficient cells. Video 3 shows NEBD during normal mitosis. Video 4 shows the absence of NEBD in cyclin B-depleted cells.

## Supplementary Material

SourceData F1 is the source file for Fig. 1.

SourceData F4 is the source file for Fig. 4.

SourceData F5 is the source file for Fig. 5.

SourceData F6 is the source file for Fig. 6.

SourceData F7 is the source file for Fig. 7.

SourceData FS1 is the source file for Fig. S1.

SourceData FS2 is the source file for Fig. S2.

SourceData FS3 is the source file for Fig. S3.

SourceData FS4 is the source file for Fig. S4.

## Data Availability

All primary data are available upon request.
