# Supplementary figures and images for: Plasticity of mitotic cyclins in promoting the G2–M transition
Source: J Cell Biol. 2025 Apr 9;224(6):e202409219. doi: 10.1083/jcb.202409219 (PMC11980681; doi:10.1083/jcb.202409219)

Figure 1A

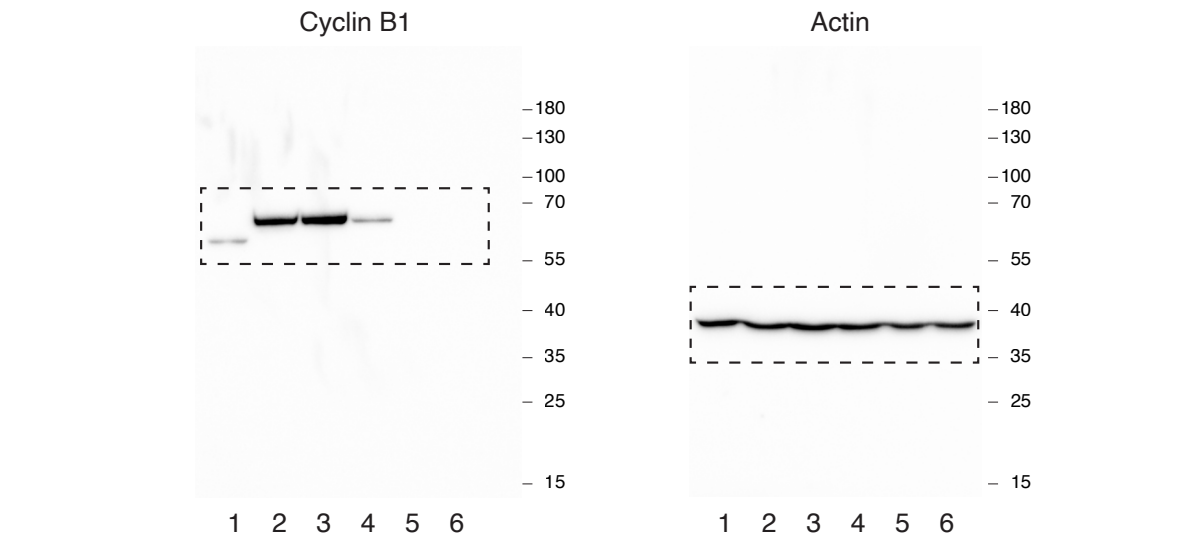

Figure 1B

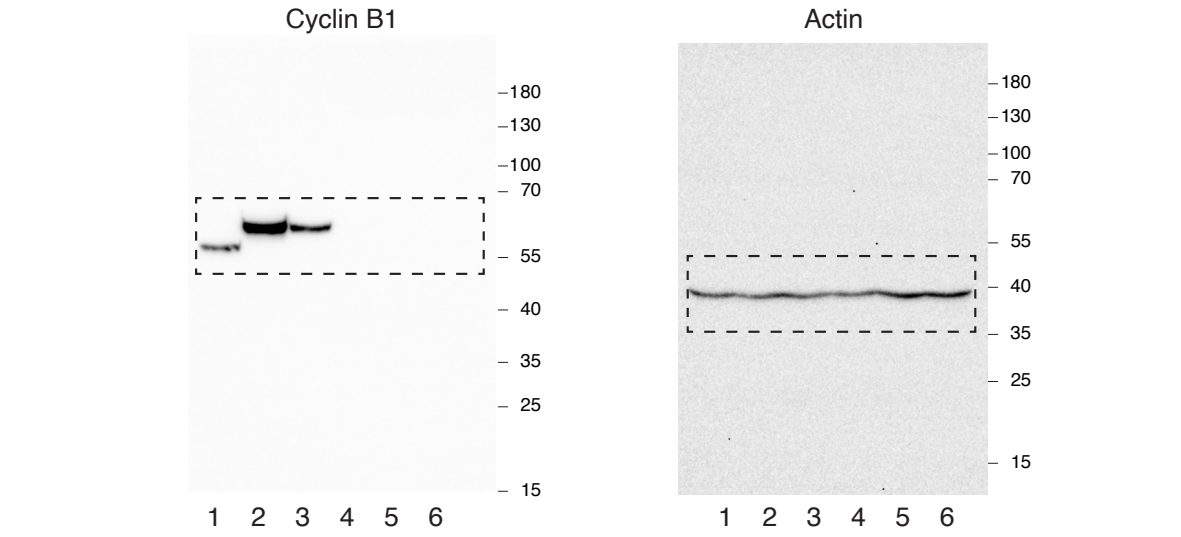

Figure 1C

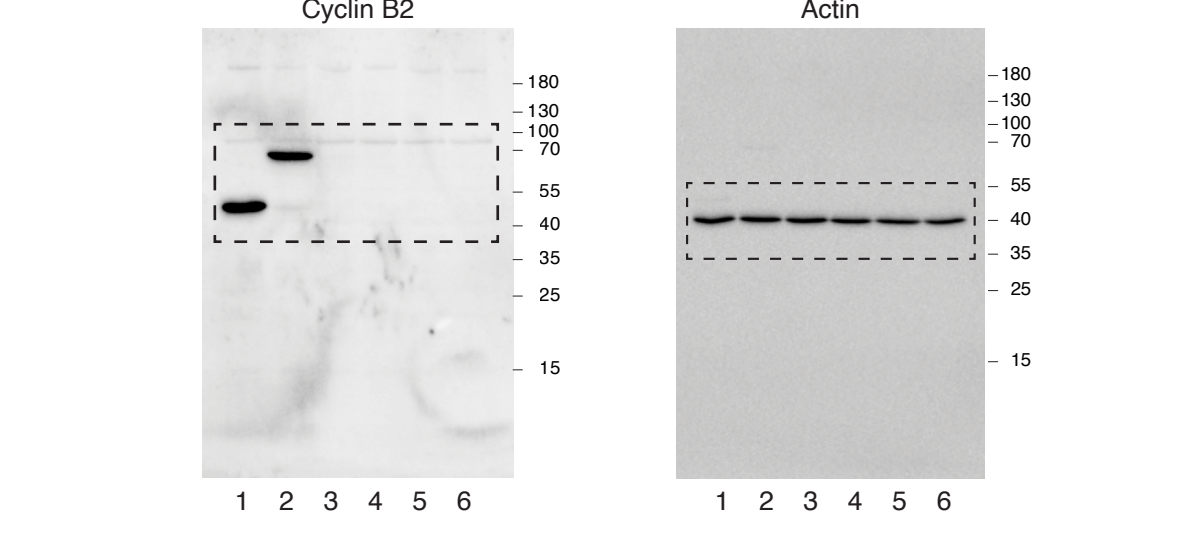

Figure 1D

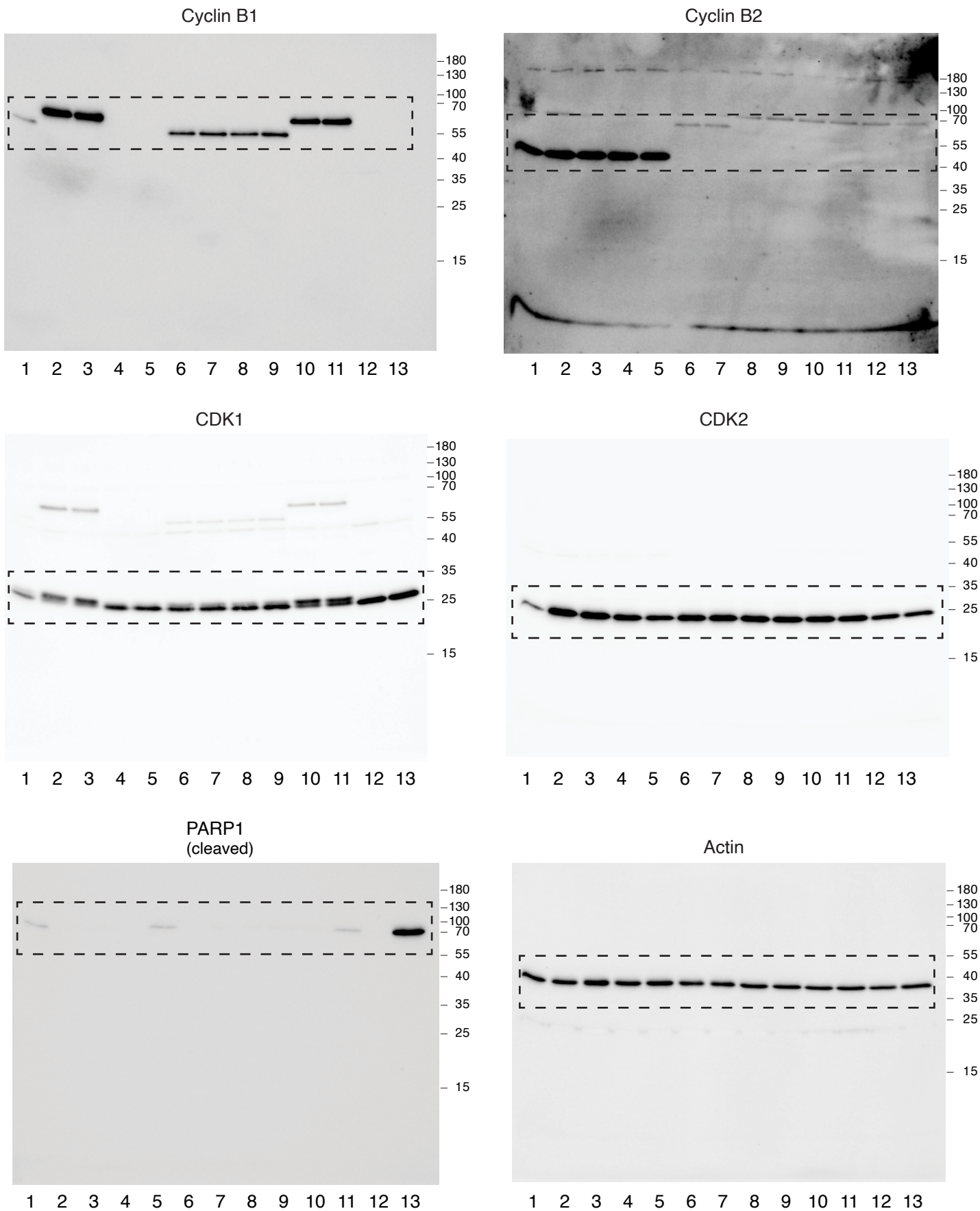

Supplement: SourceData F1 — is the source file for Fig. 1. [file jcb_202409219_sourcedataf1.pdf]

Figure S4B

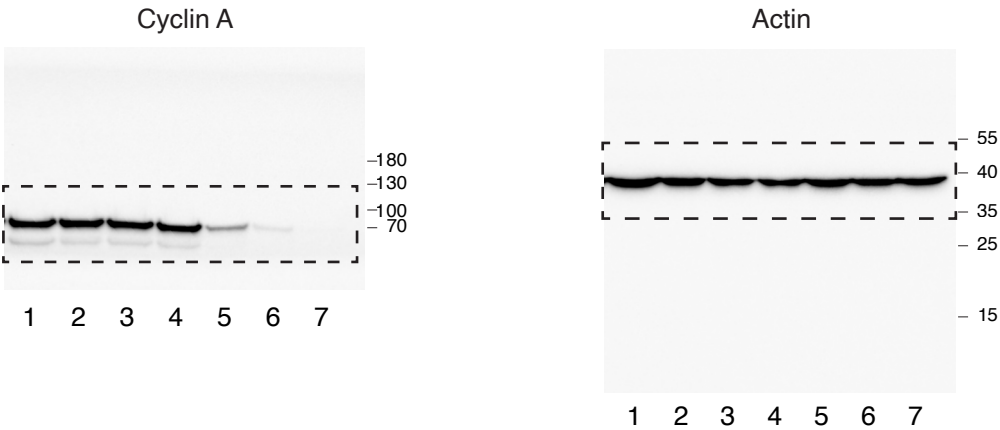

Figure S4D

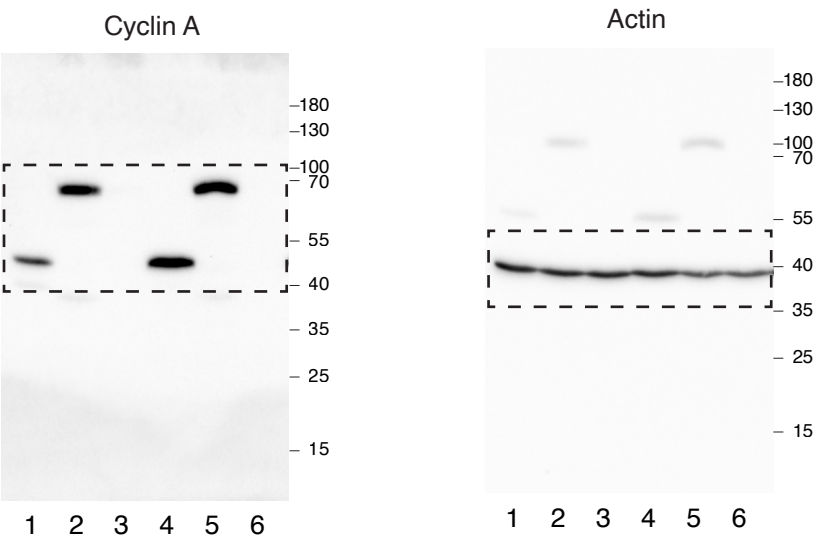

Figure S4F

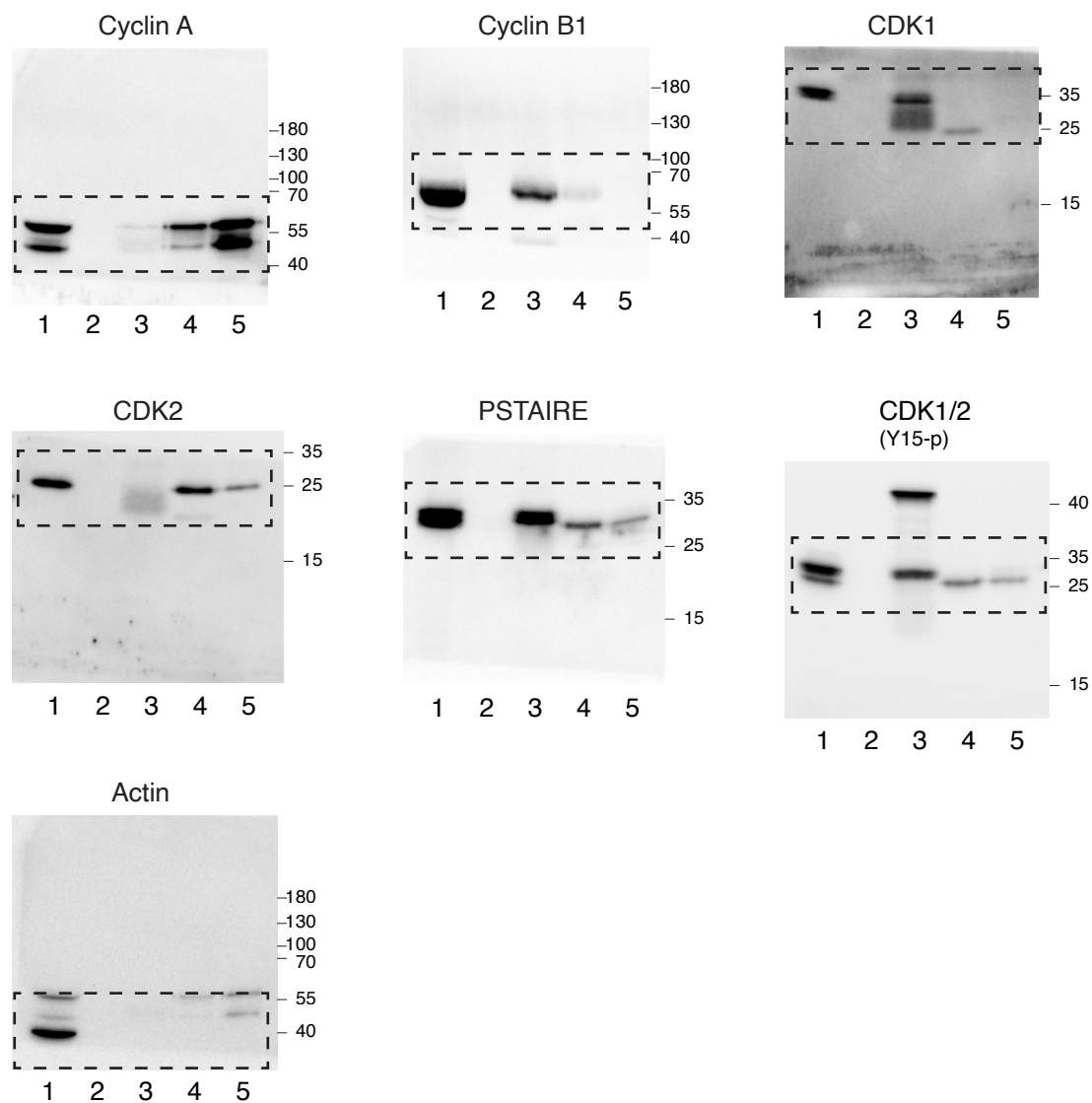

Supplement: SourceData F4 — is the source file for Fig. 4. [file jcb_202409219_sourcedataf4.pdf]

Figure S5A

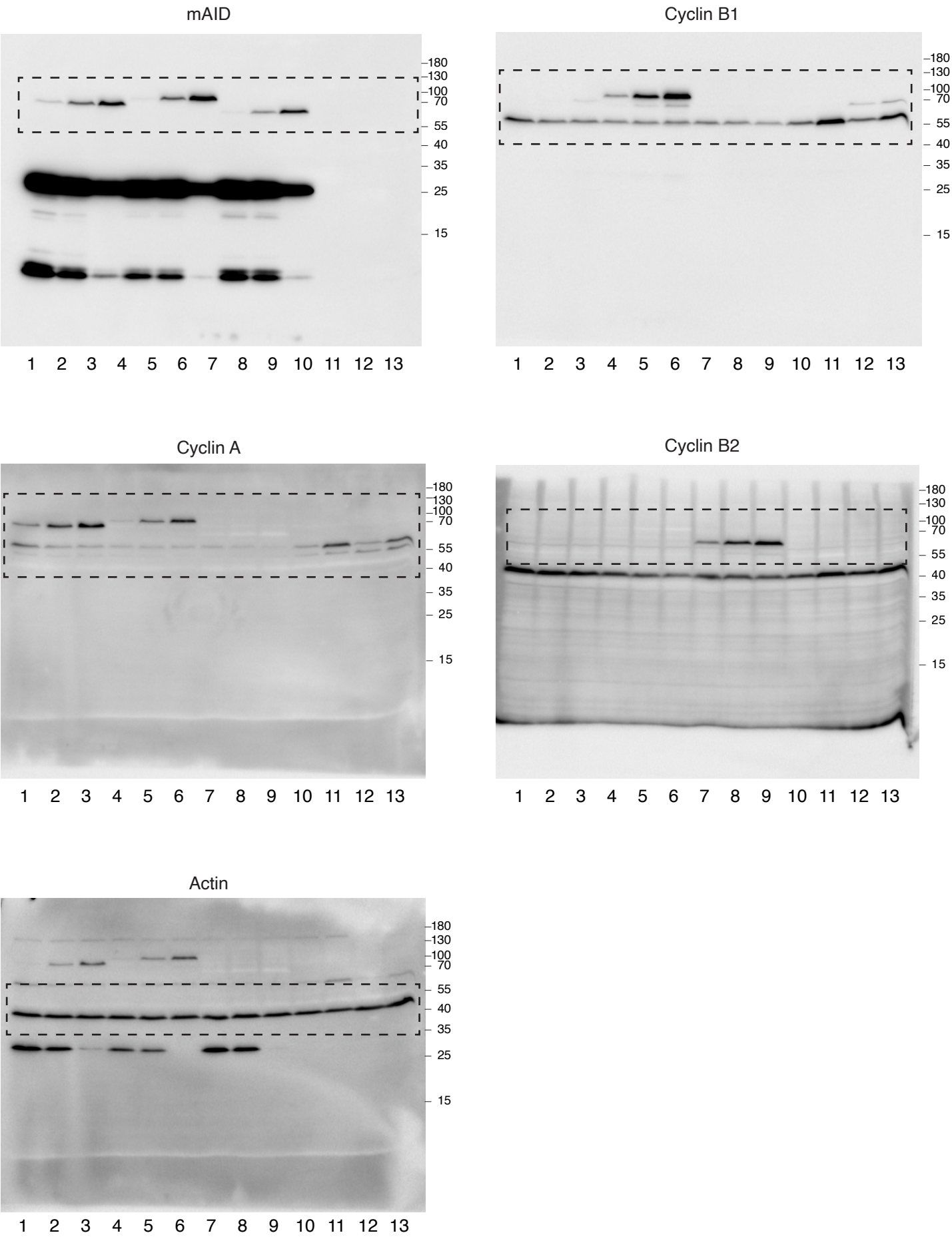

Figure S5B

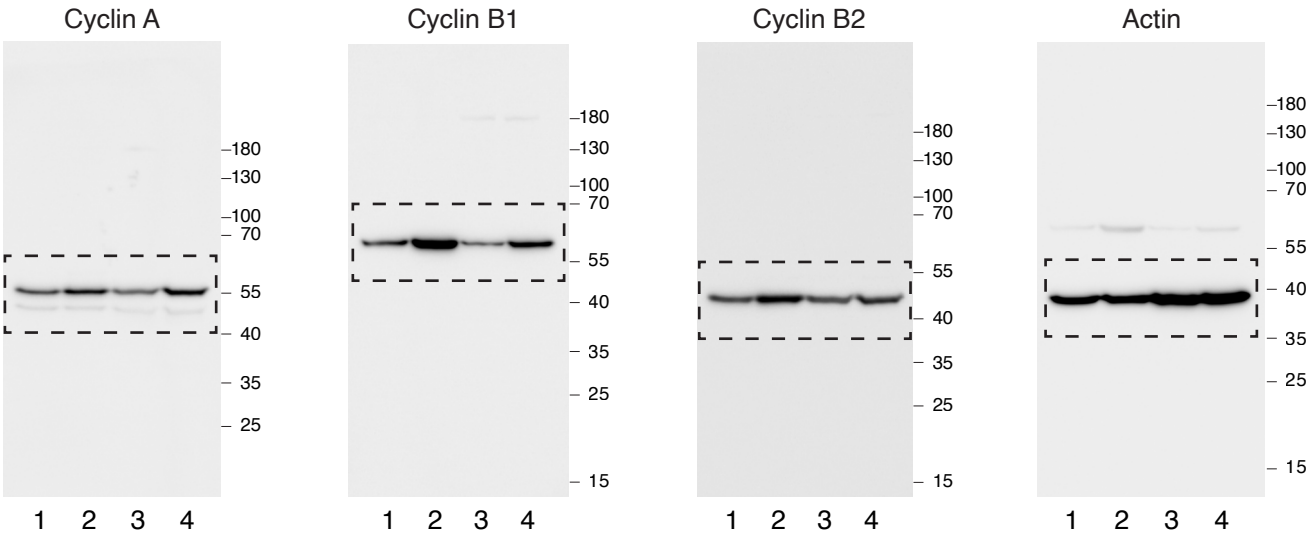

Supplement: SourceData F5 — is the source file for Fig. 5. [file jcb_202409219_sourcedataf5.pdf]

Figure 6 Original Source Data

Figure 6A

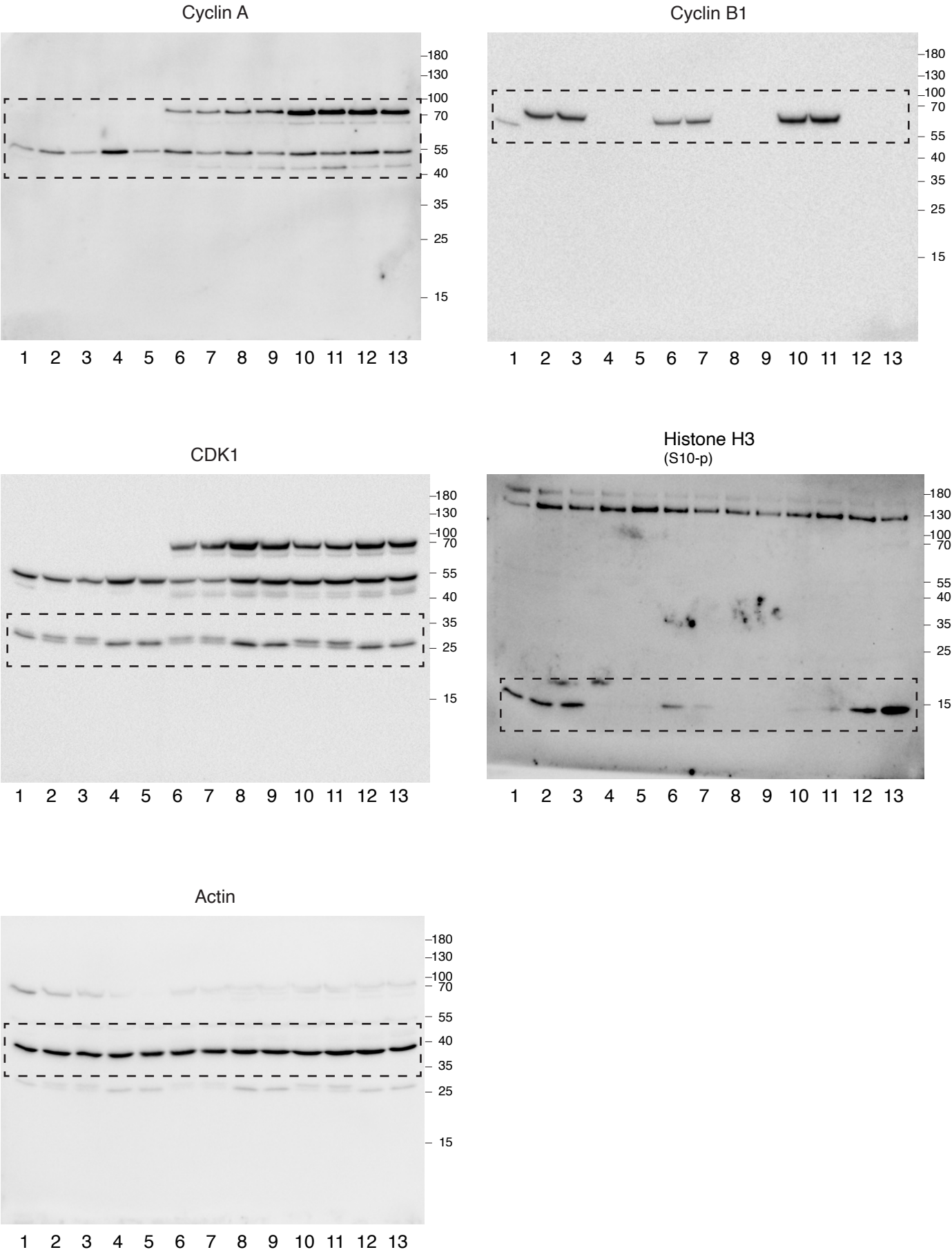

Figure 6F

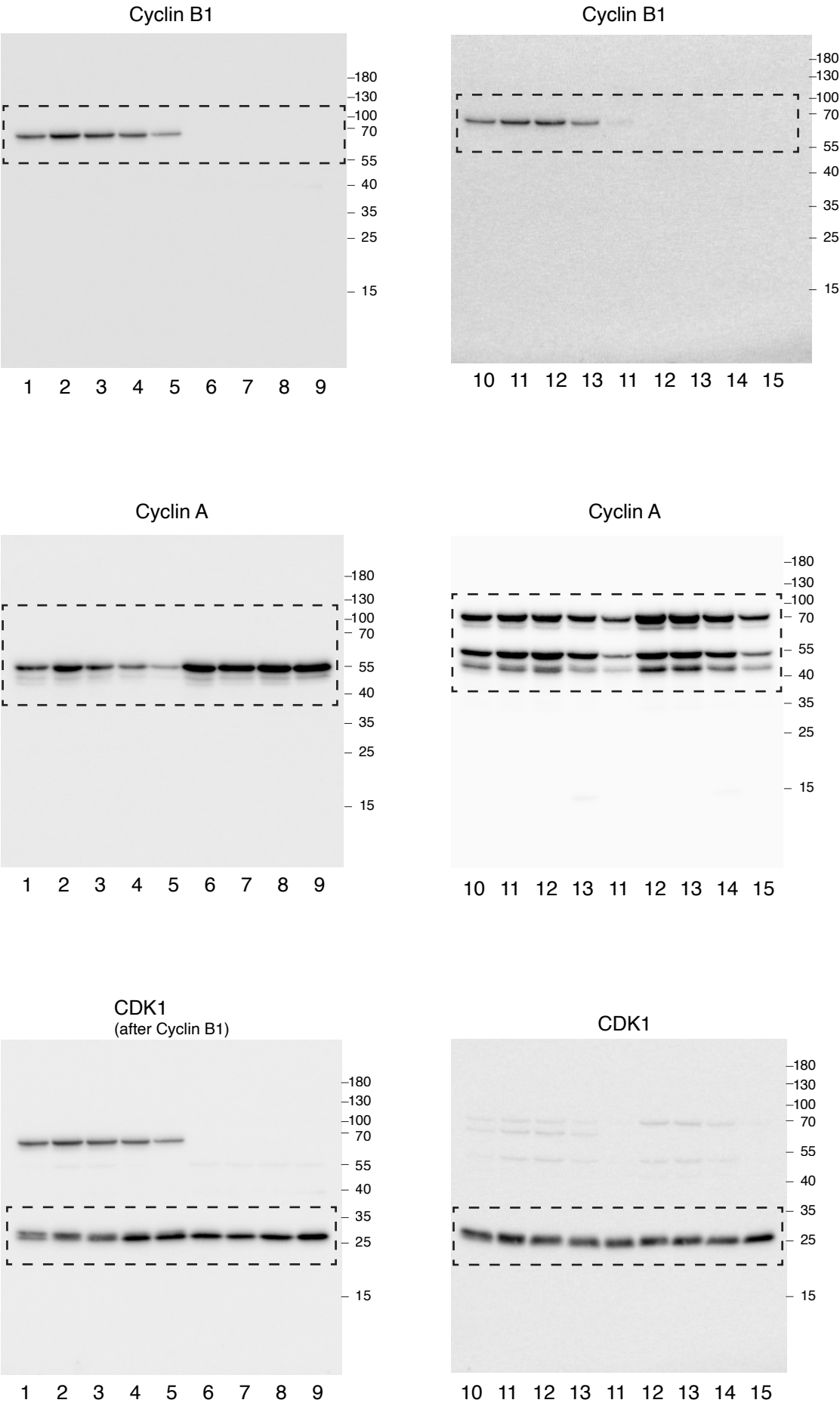

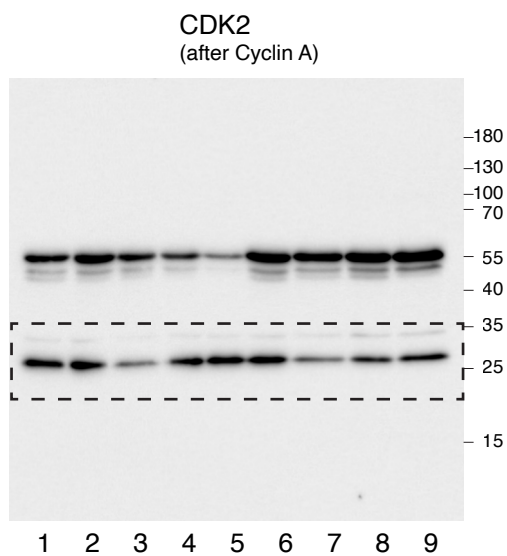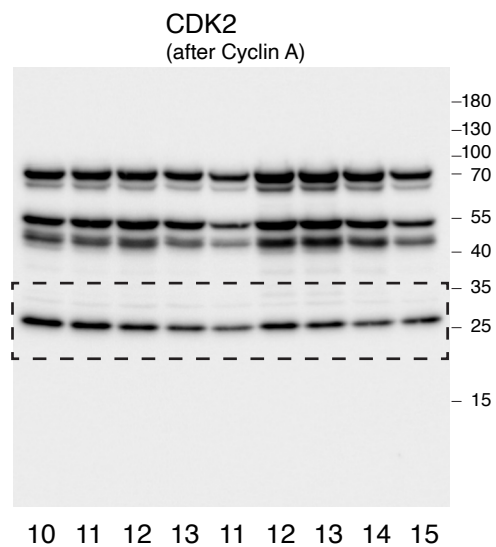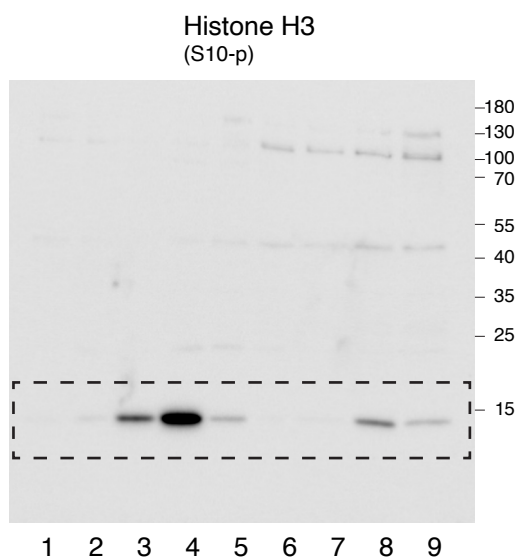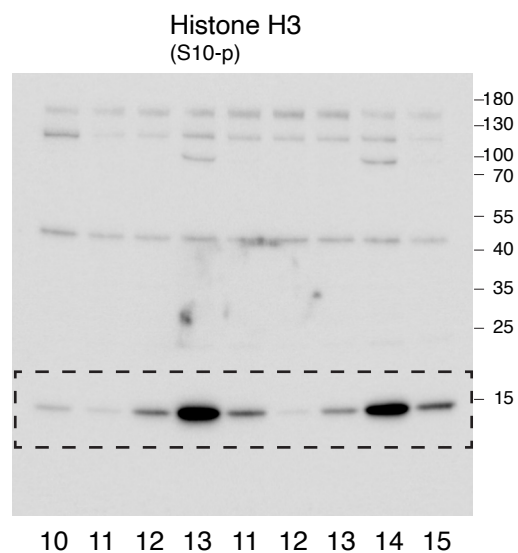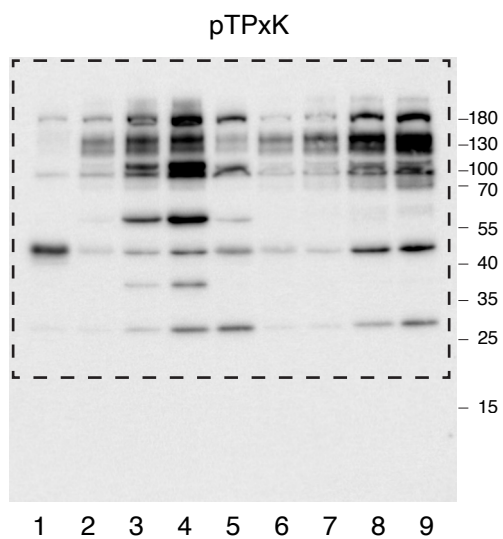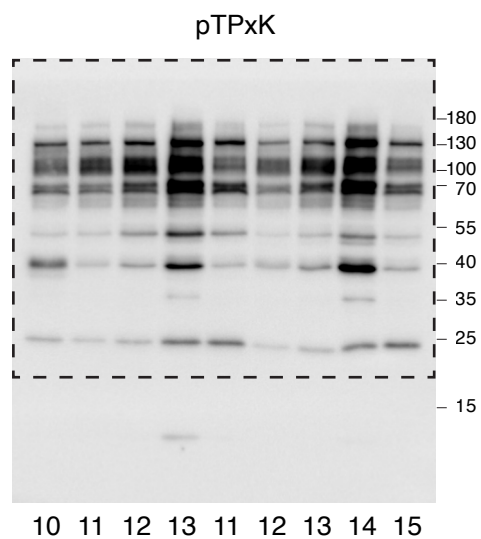

PTTG1

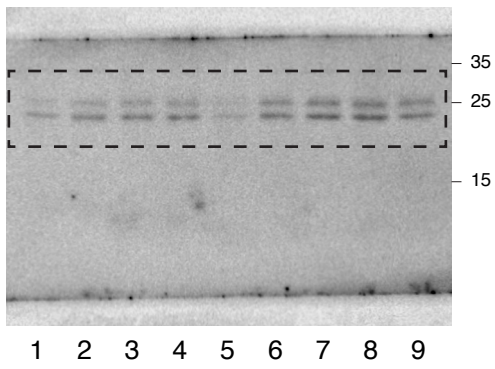

PTTG1

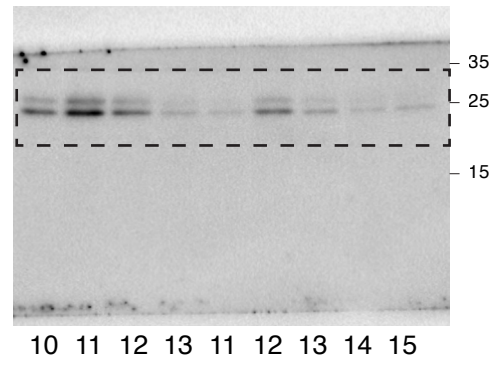

Actin

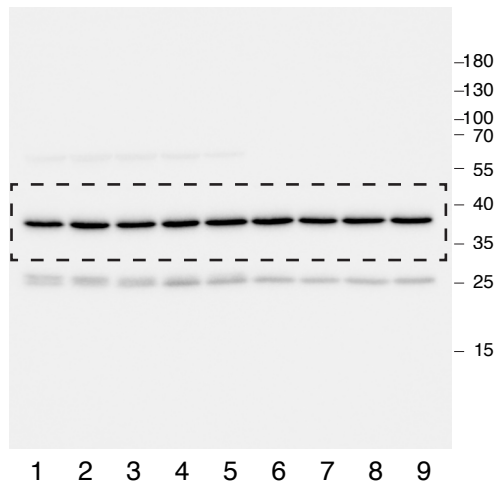

Actin

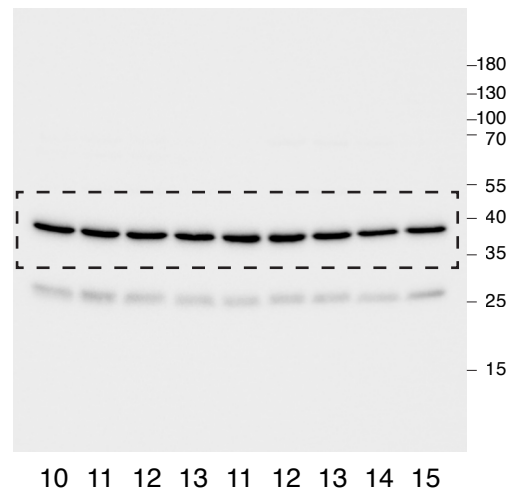

Figure 6G

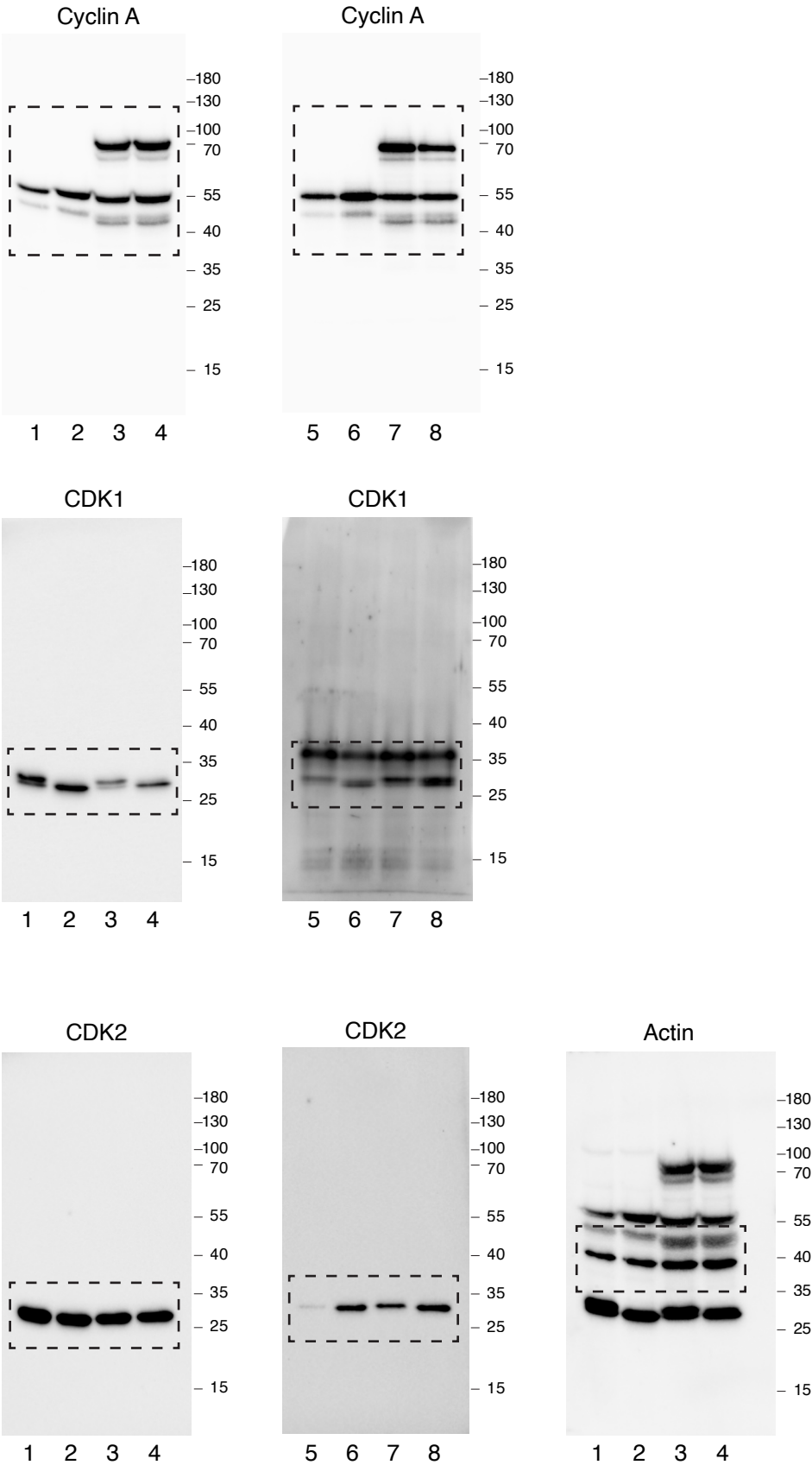

Figure 6H

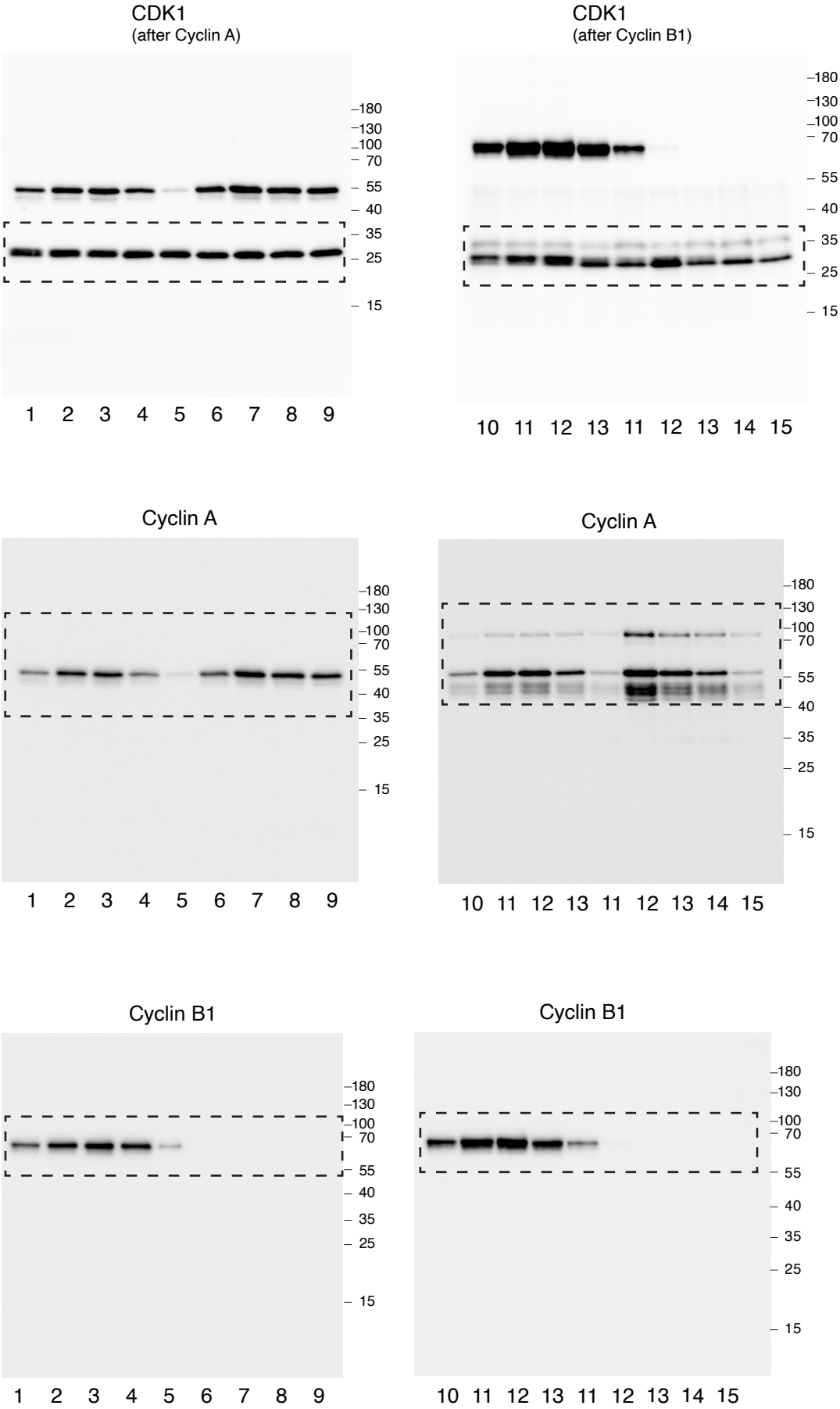

Supplement: SourceData F6 — is the source file for Fig. 6. [file jcb_202409219_sourcedataf6.pdf]

Figure 7A

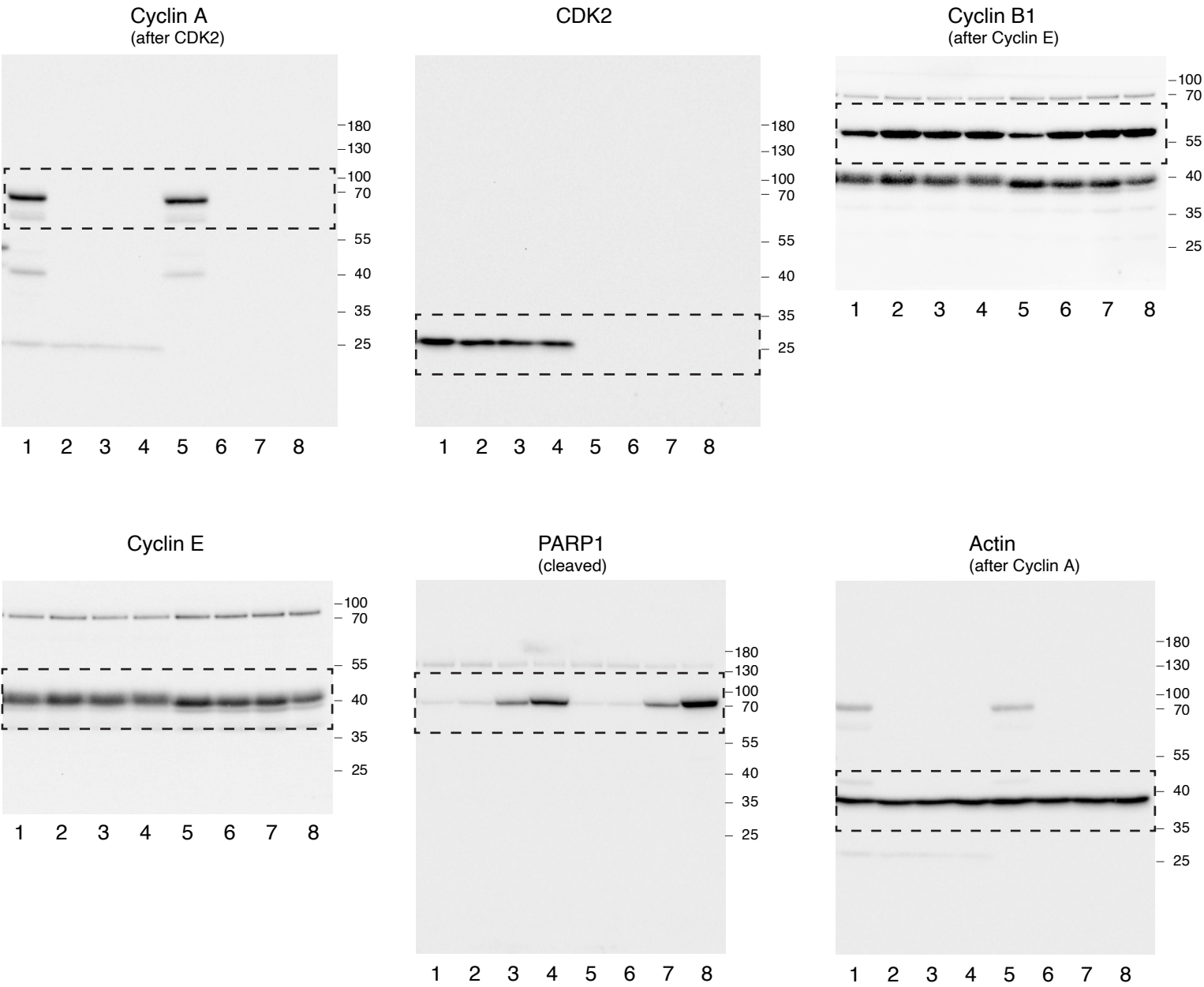

Figure 7G

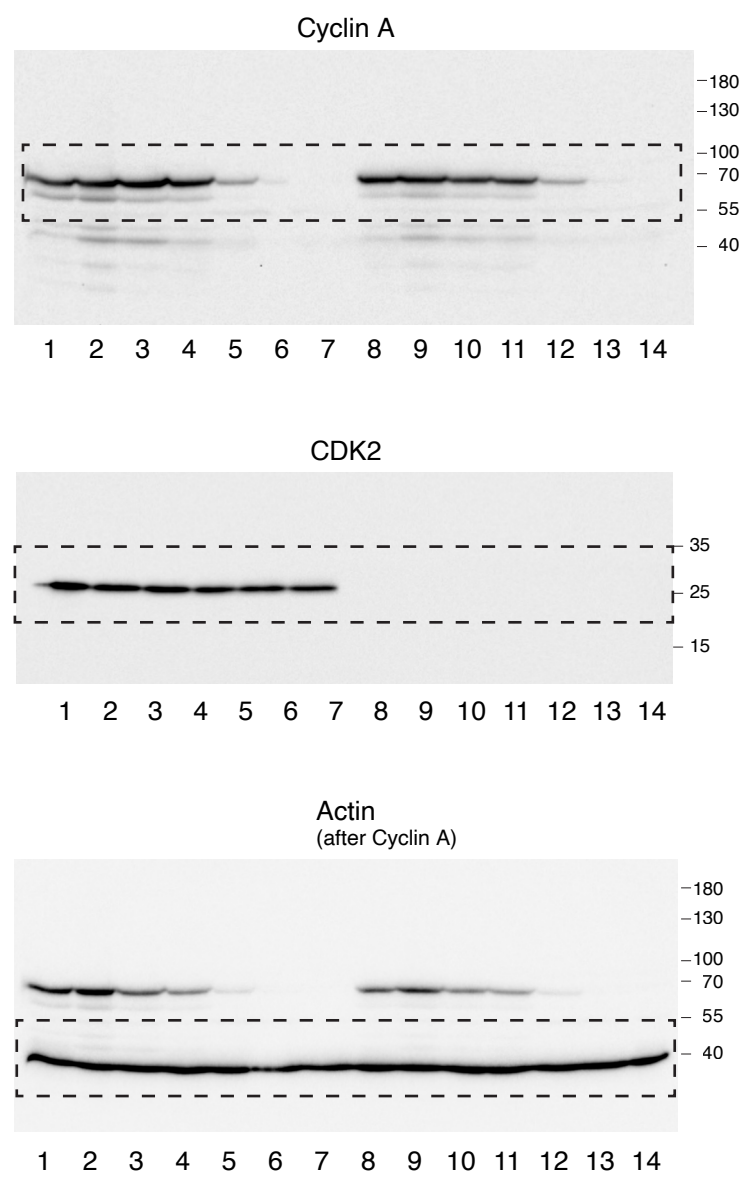

### Figure 7H

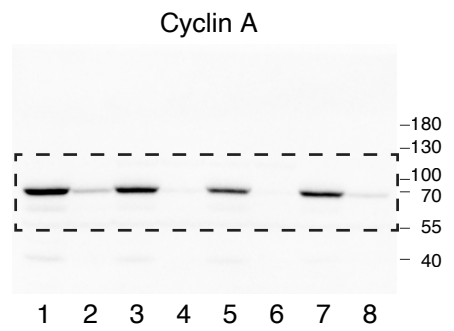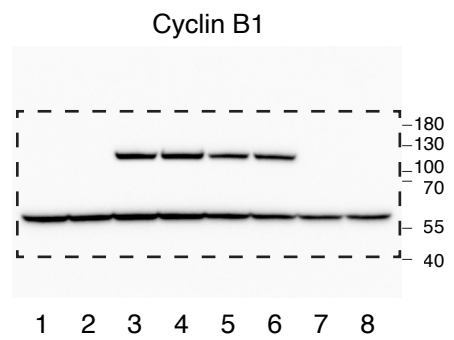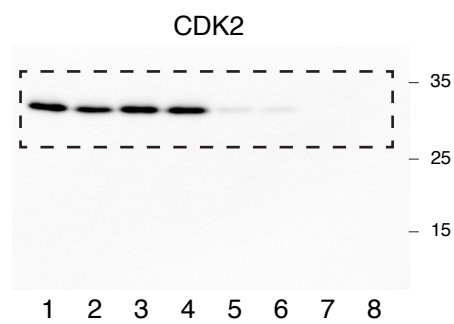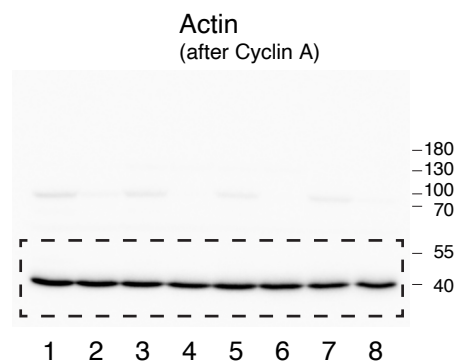

Figure 7I

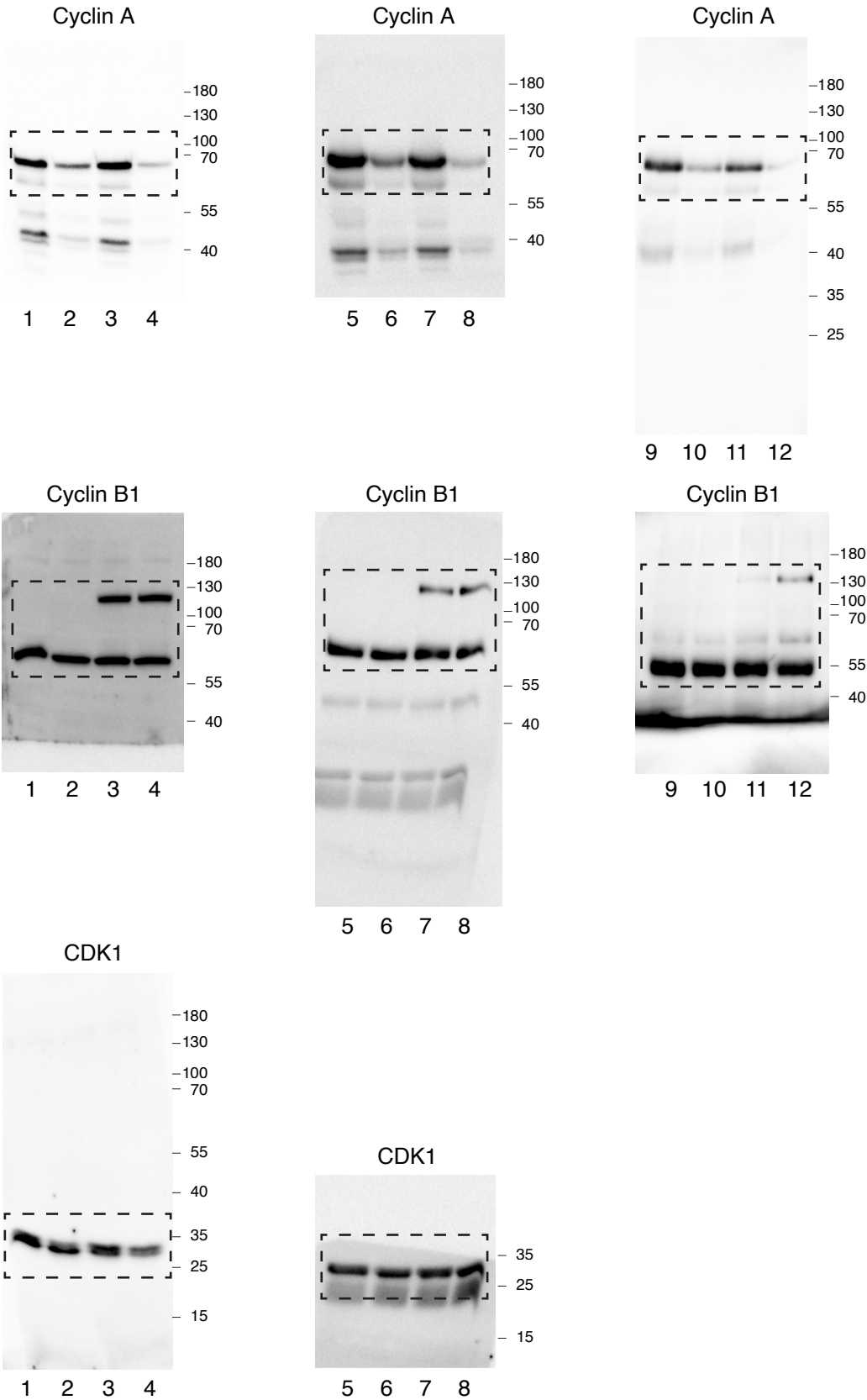

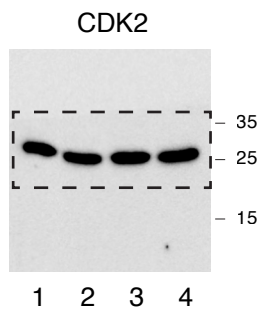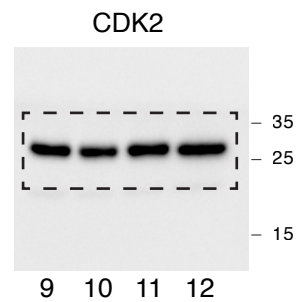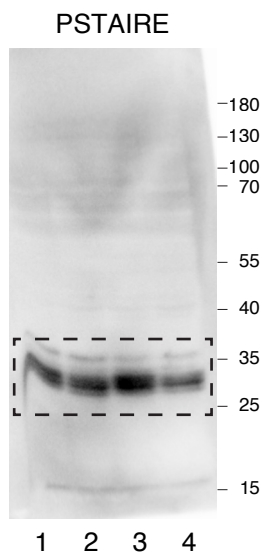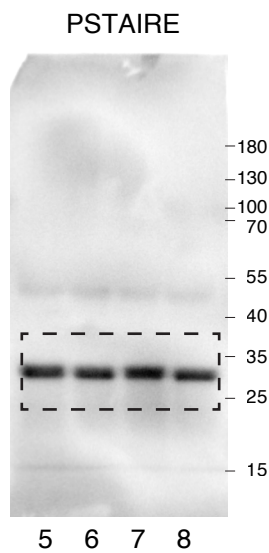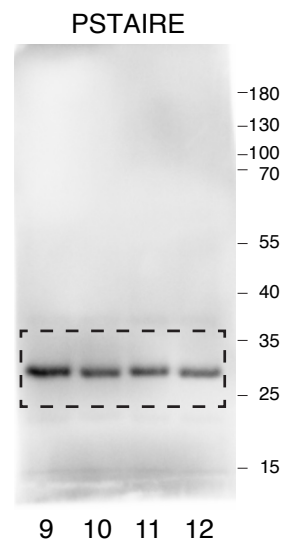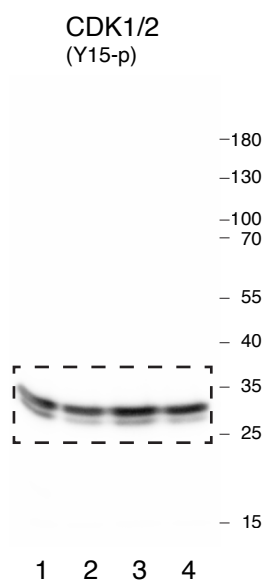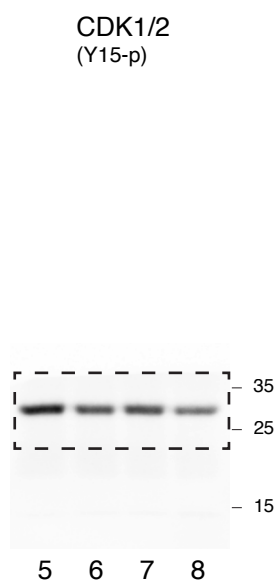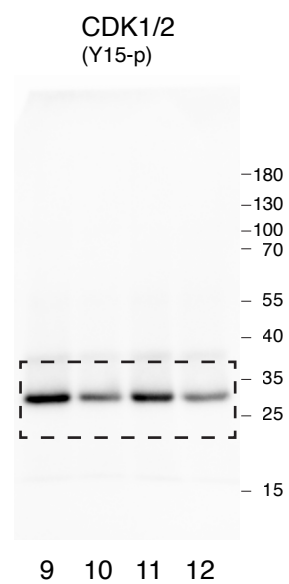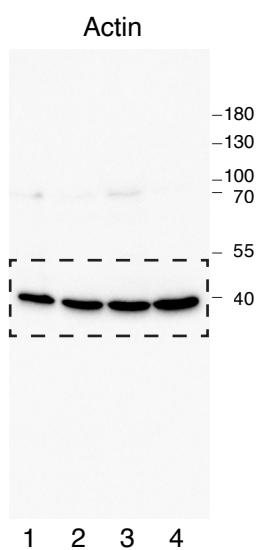

Supplement: SourceData F7 — is the source file for Fig. 7. [file jcb_202409219_sourcedataf7.pdf]

Figure S1B

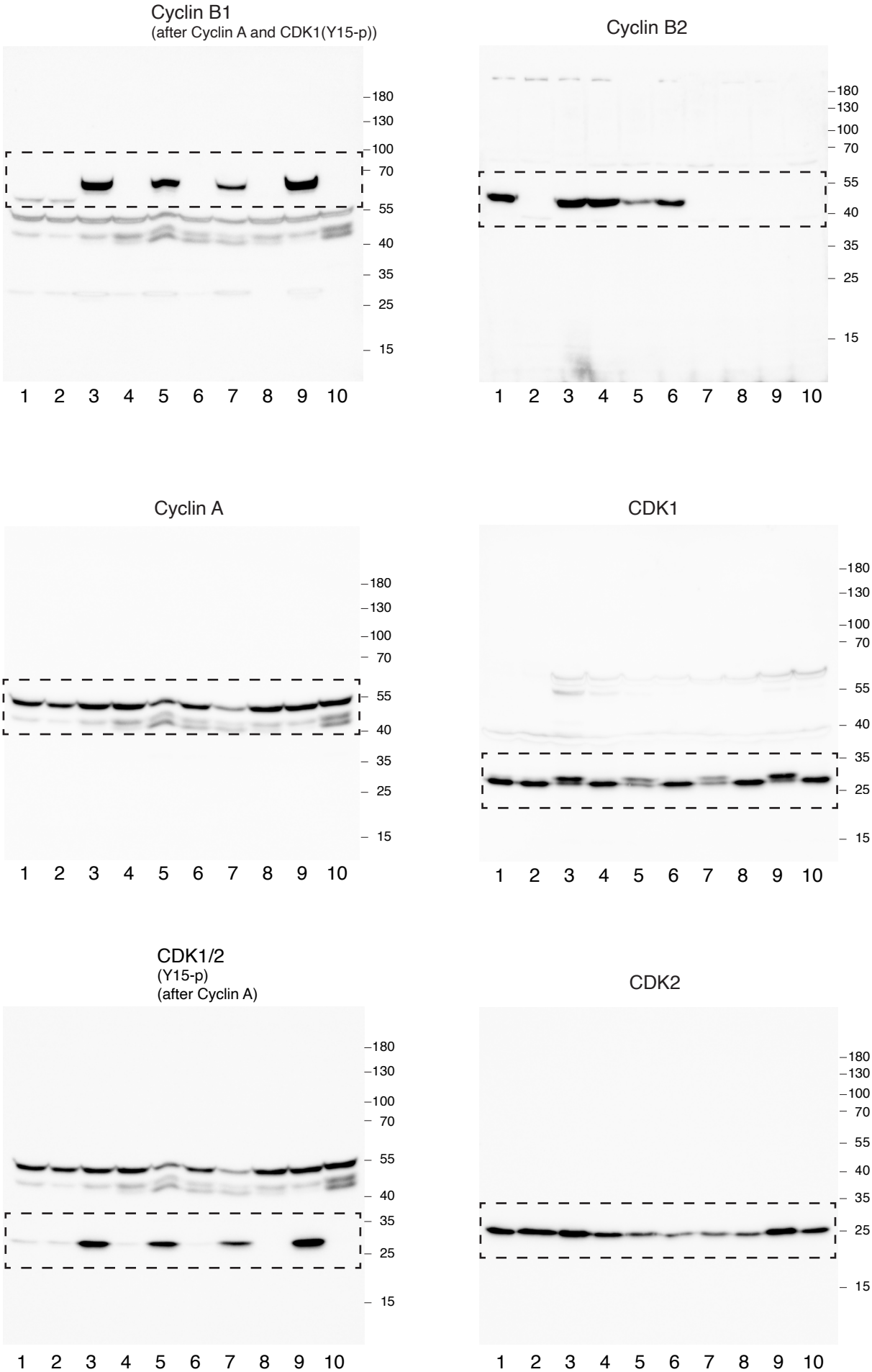

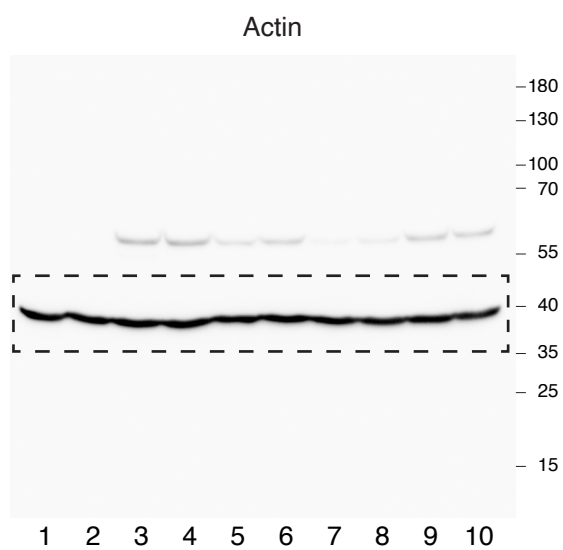

**Figure S1C**

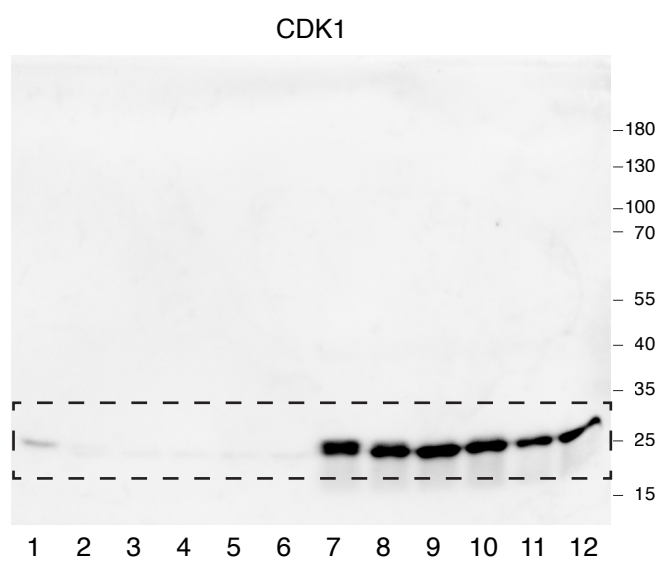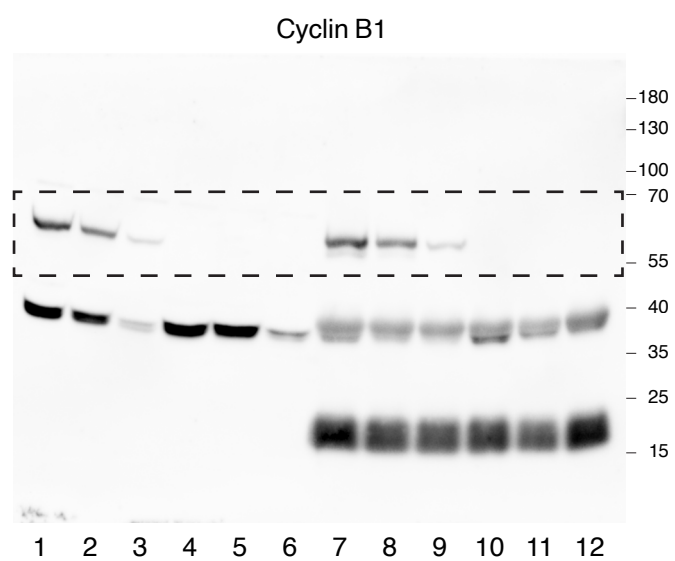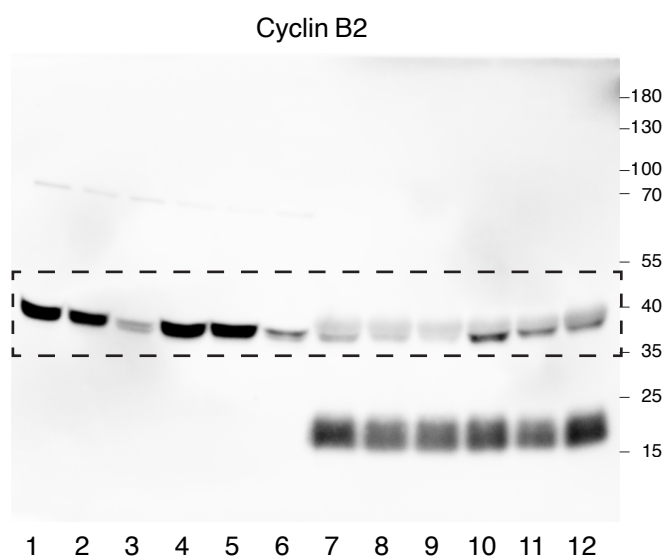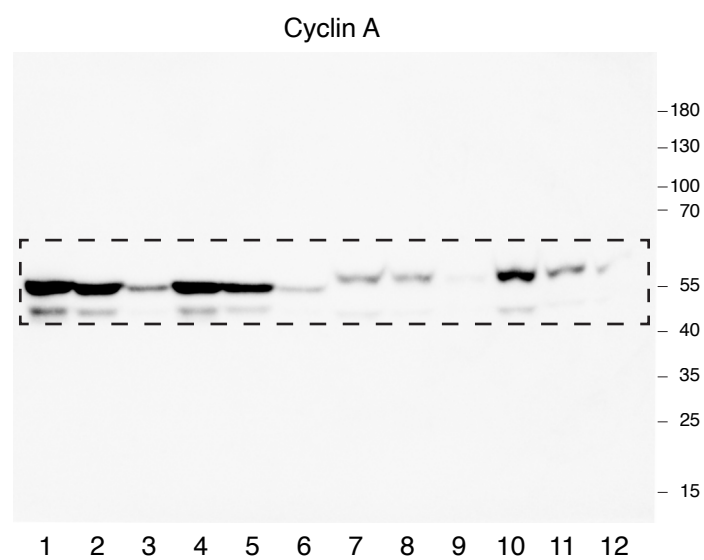

Figure S1E

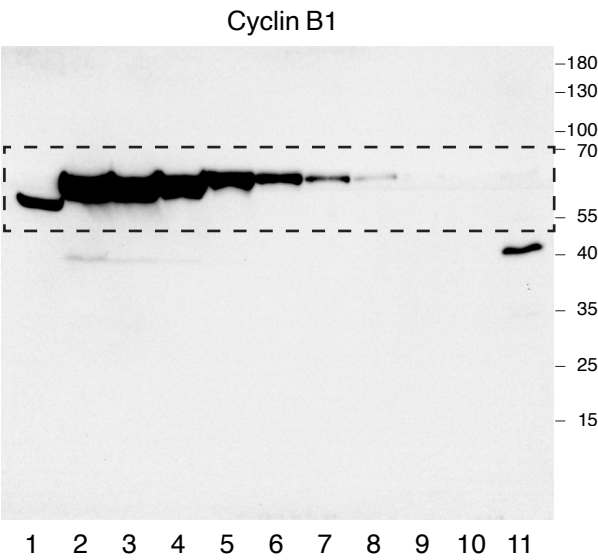

Supplement: SourceData FS1 — is the source file for Fig. S1. [file jcb_202409219_sourcedatafs1.pdf]

Figure S2D

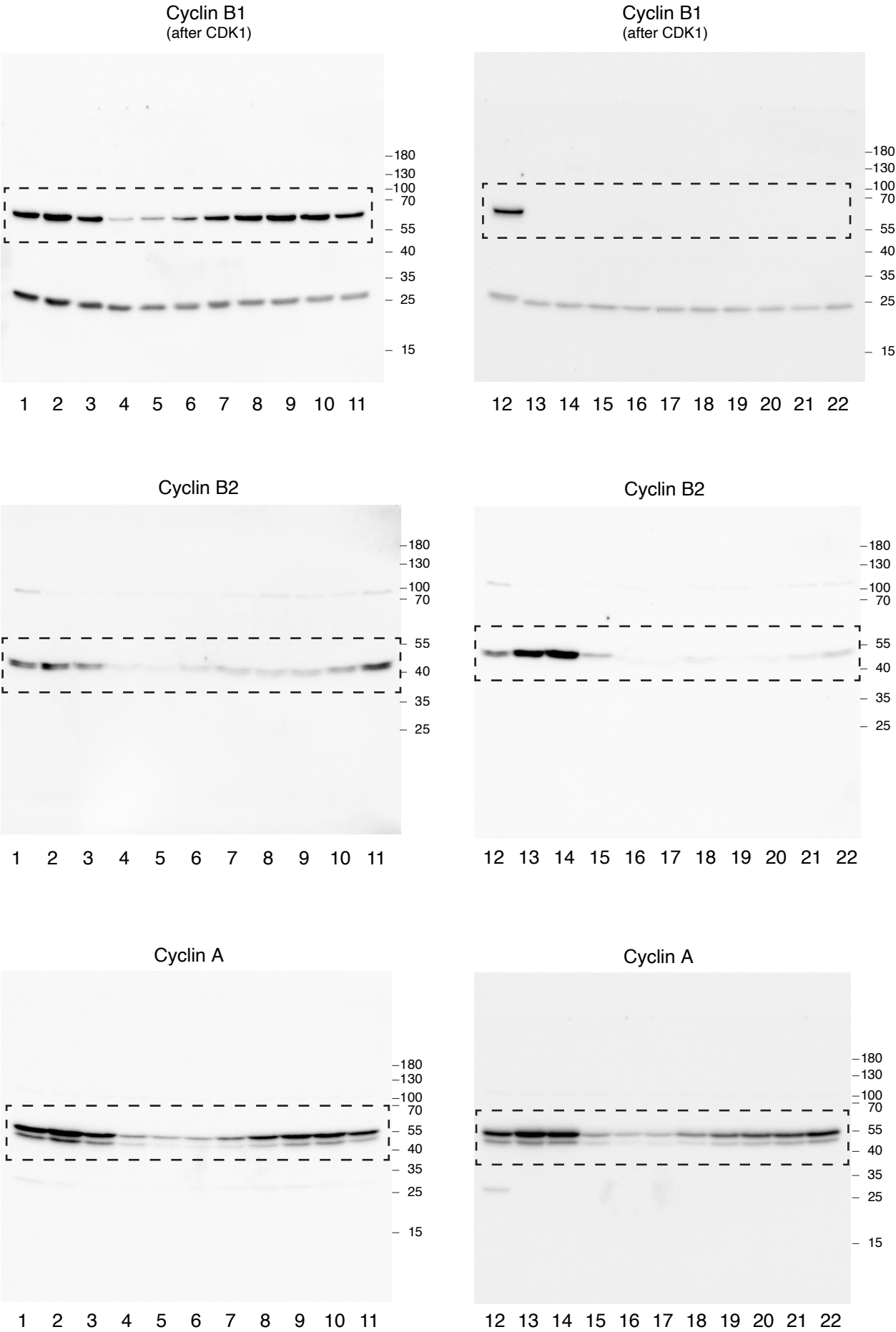

CDK1

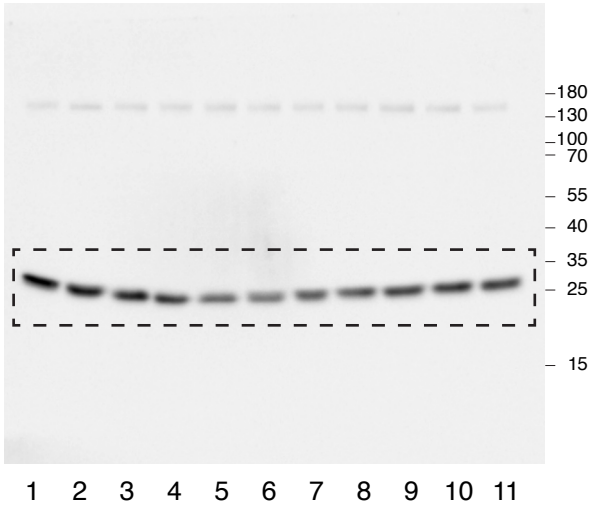

CDK1

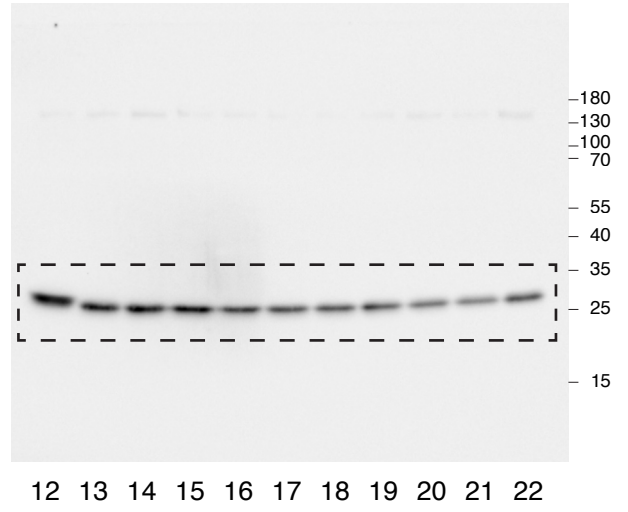

Histone H3  
(S10-p)

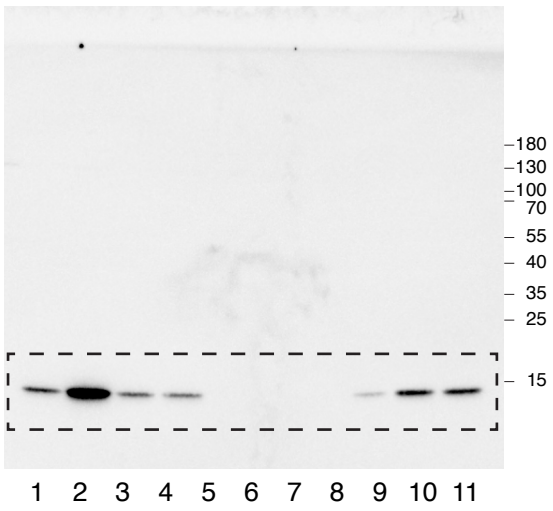

Histone H3  
(S10-p)

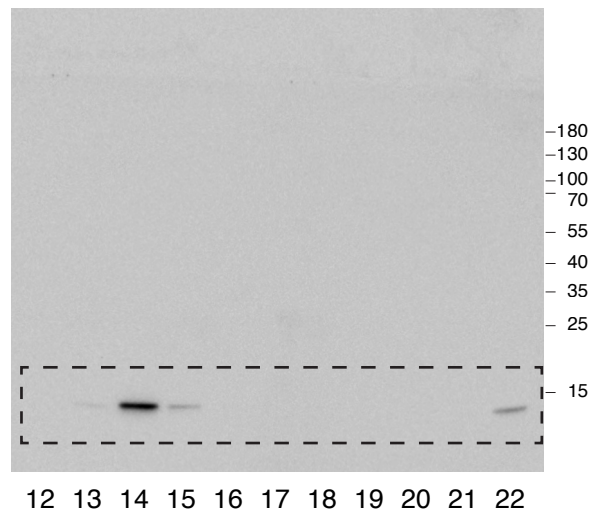

Actin

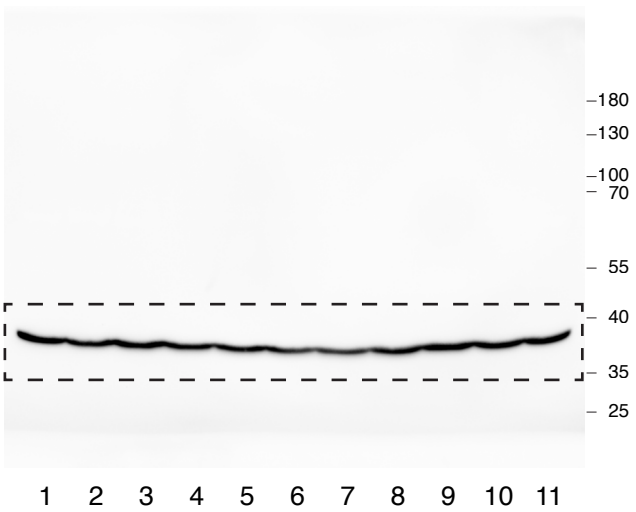

Actin

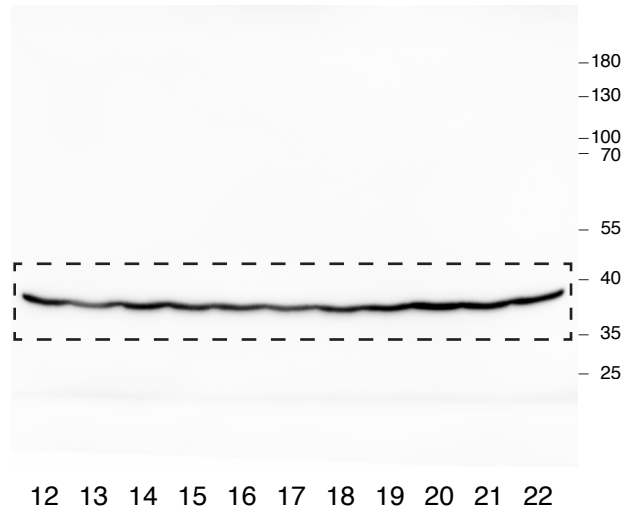

Figure S2E

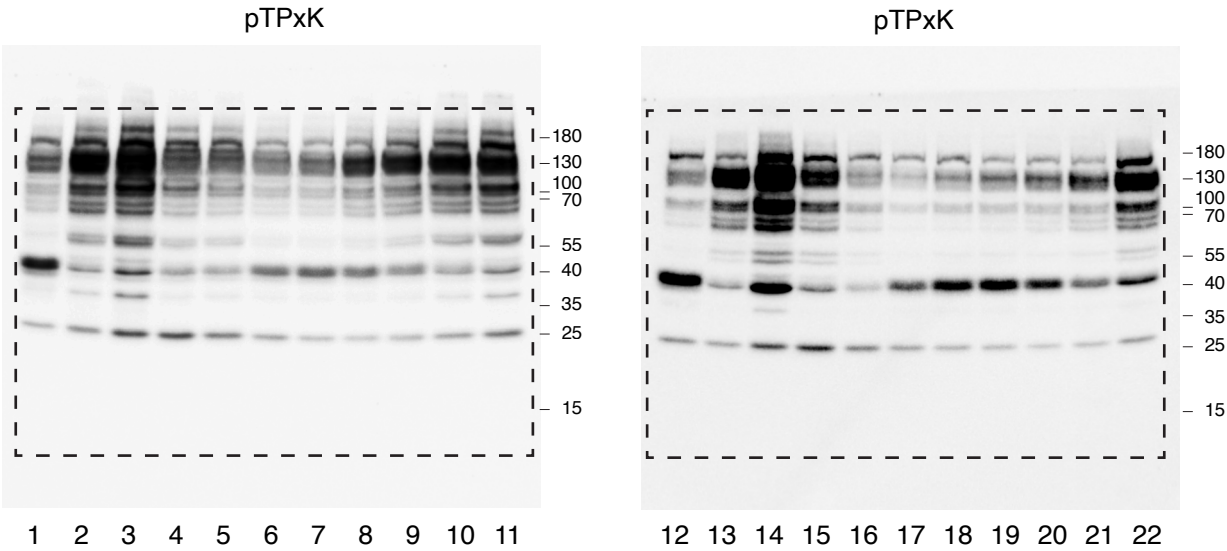

Supplement: SourceData FS2 — is the source file for Fig. S2. [file jcb_202409219_sourcedatafs2.pdf]

Figure S3A

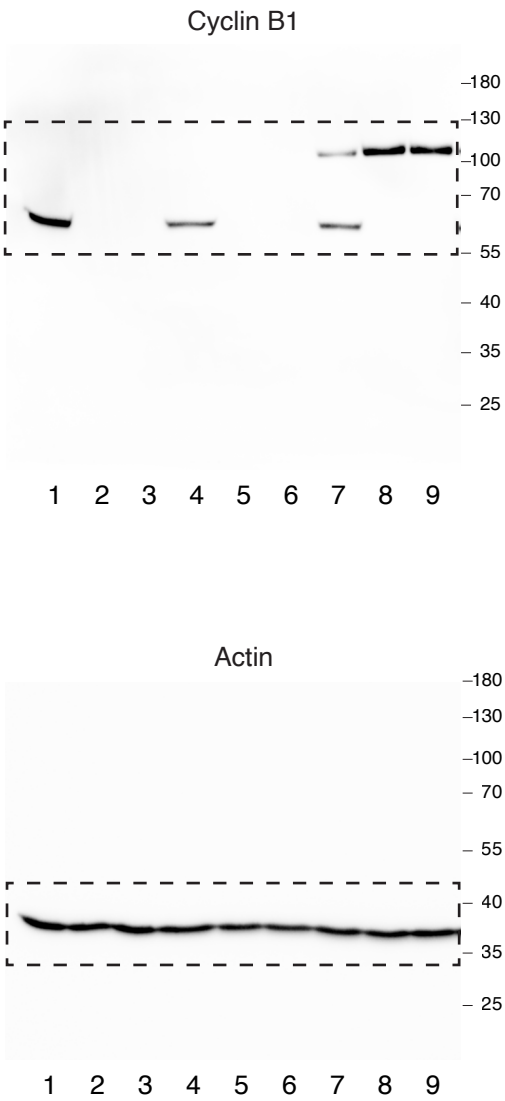

Figure S3C

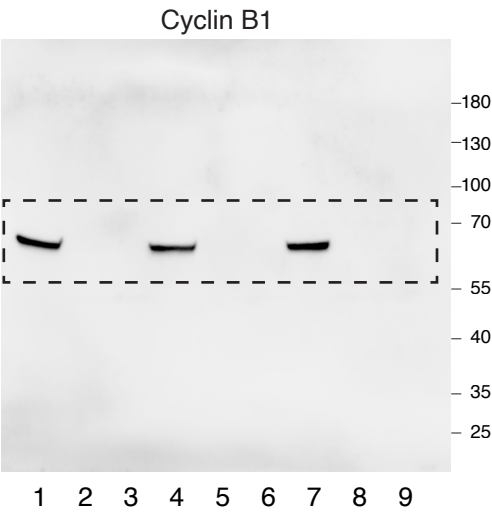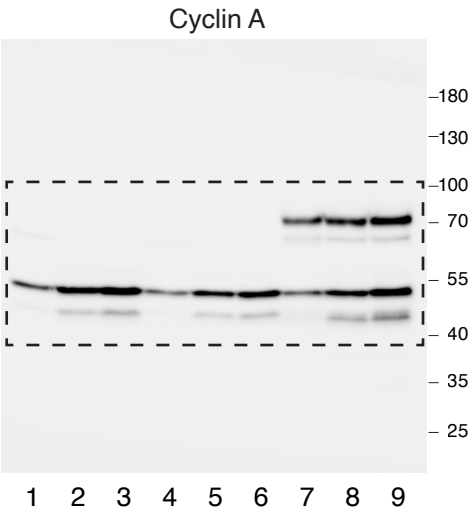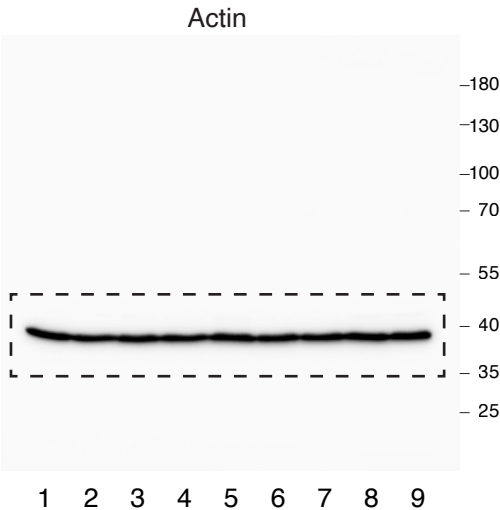

Figure S3D

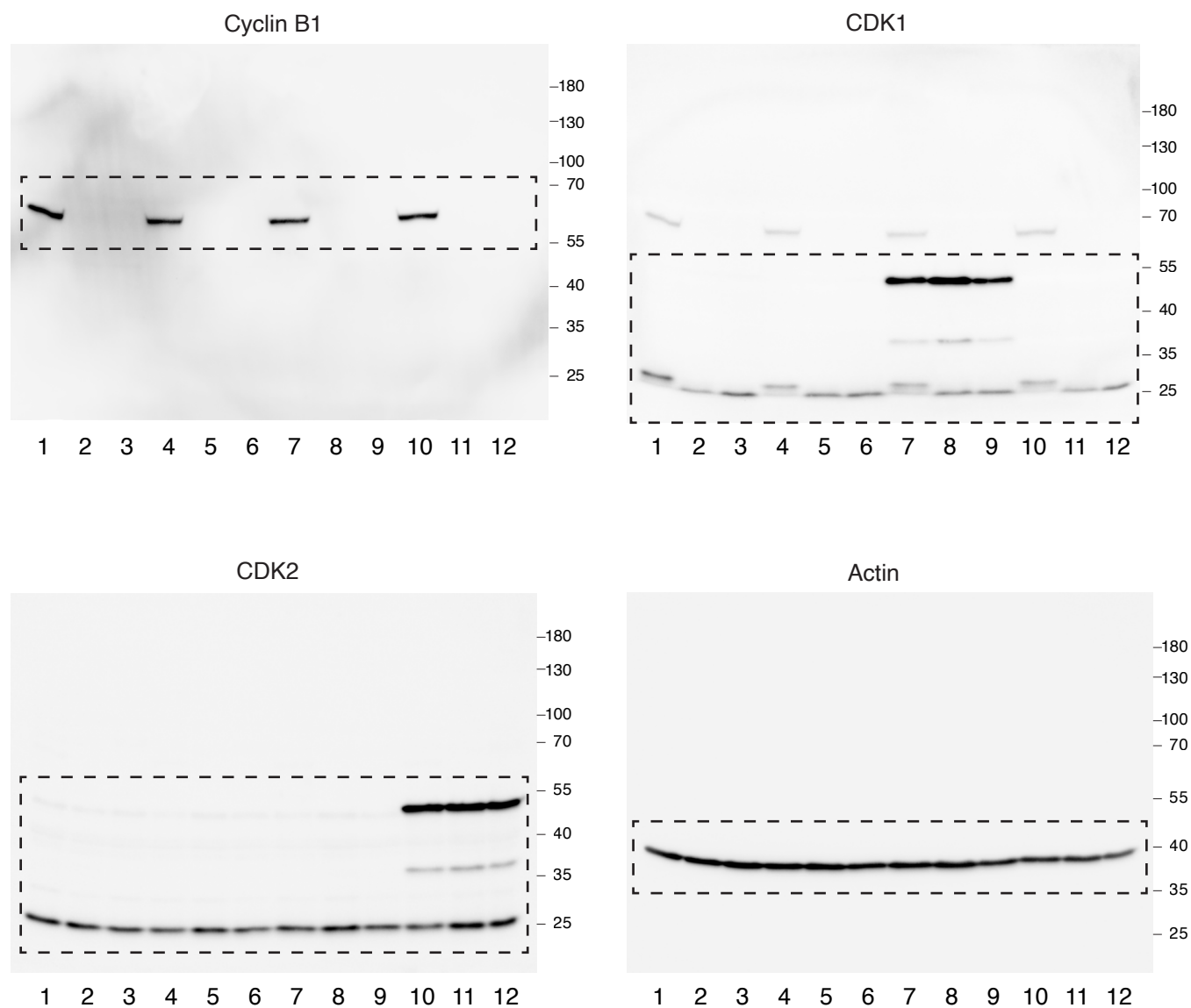

Supplement: SourceData FS3 — is the source file for Fig. S3. [file jcb_202409219_sourcedatafs3.pdf]

Figure S4D

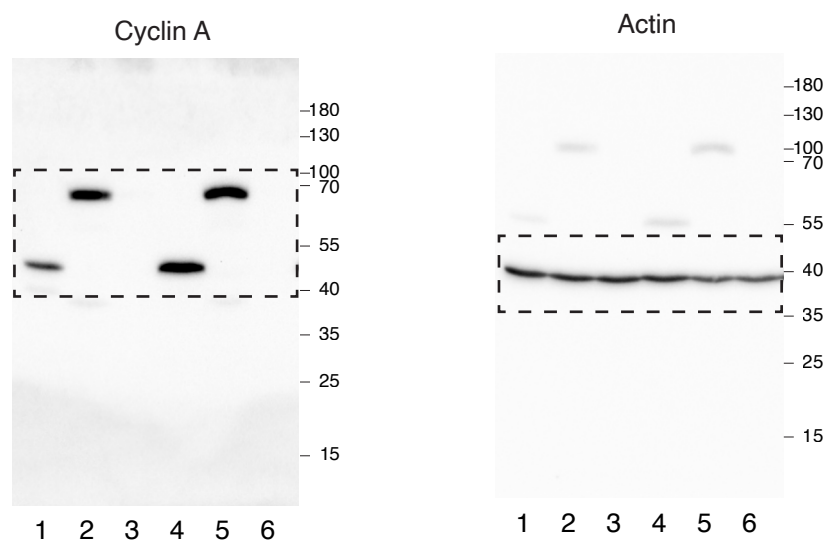

Figure S4F

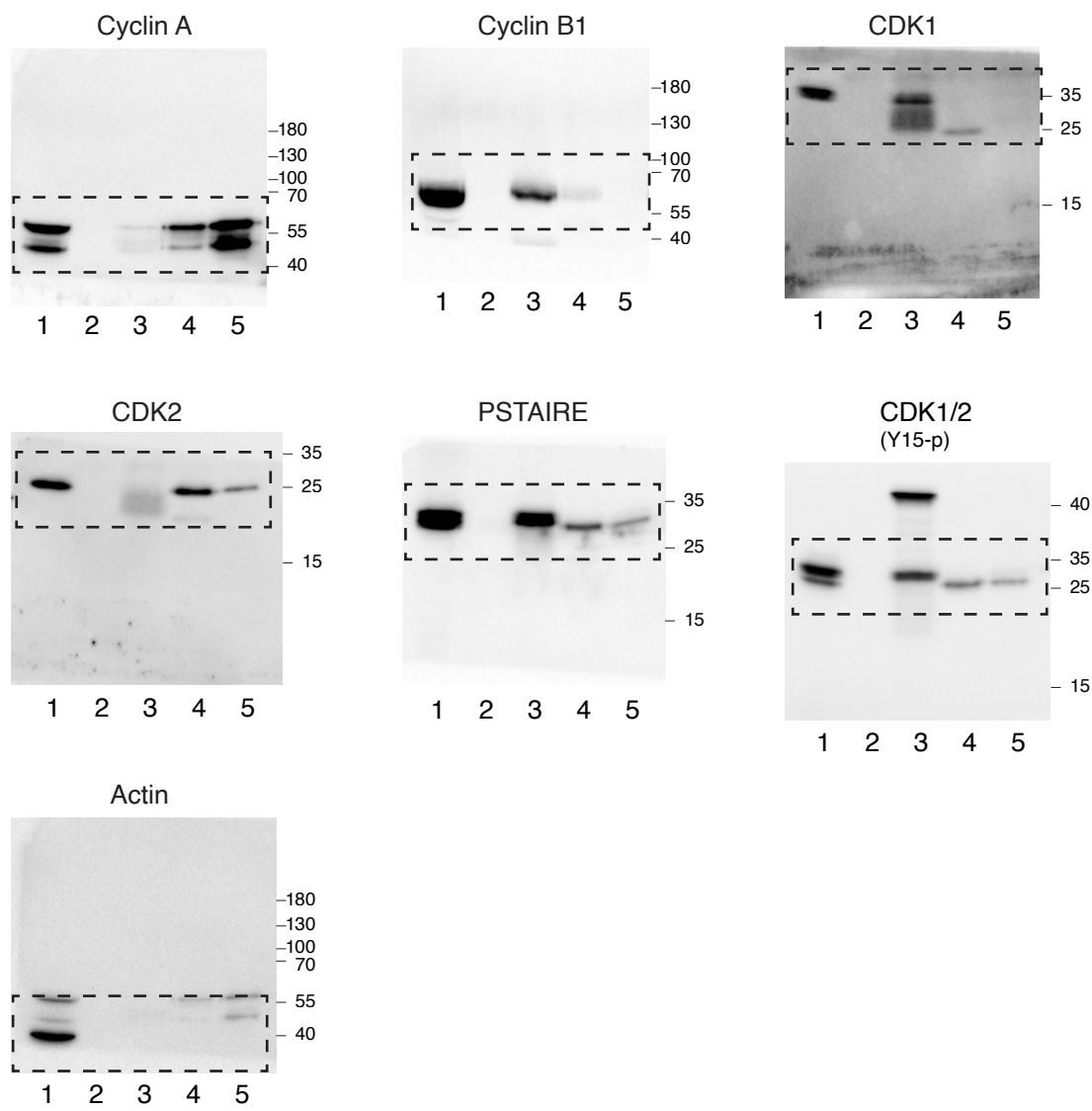

Supplement: SourceData FS4 — is the source file for Fig. S4. [file jcb_202409219_sourcedatafs4.pdf]
